# Proteomic analysis links truncated tau to lysosome motility, autophagy, and endo‐lysosomal dysfunction

**DOI:** 10.1002/alz.70977

**Published:** 2025-12-15

**Authors:** Despoina Goniotaki, Maximilian Hausherr, Steven Lynham, Ayushin Ale, George Chennell, Stefania Marcotti, Katrin Marcus, Wendy Noble, Diane P. Hanger, Graham Fraser, Deepak P. Srivastava

**Affiliations:** ^1^ Department of Basic and Clinical Neuroscience, Institute of Psychiatry, Psychology & Neuroscience Maurice Wohl Clinical Neuroscience Institute King's College London London UK; ^2^ Medizinisches Proteom‐Center Medical Faculty Ruhr University Bochum Bochum Germany; ^3^ Medical Proteome Analysis, Center for Proteindiagnostics (PRODI) Ruhr University Bochum Bochum Germany; ^4^ Proteomics Core Facility, The James Black Centre King's College London, London London UK; ^5^ Microscopy Innovation Centre, Research Management & Innovation Directorate (RMID) King's College London, Guys Campus London UK; ^6^ Department of Clinical and Biomedical Sciences University of Exeter Exeter UK; ^7^ AstraZeneca, Discovery Centre (DISC) Cambridge Biomedical Campus Cambridge UK; ^8^ MRC Centre for Neurodevelopmental Disorders, New Hunt's House King's College London, Guy's Campus London UK

**Keywords:** autophagy‐lysosomal pathway, endocytosis, live‐cell imaging, LysoTracker, mice, proteolysis, proteomics, SH‐SY5Y cells, Tau35, tauopathies

## Abstract

**INTRODUCTION:**

Tauopathies involve progressive accumulation of abnormal tau species that disrupt the autophagy‐lysosomal pathway (ALP), critical for degrading intracellular macromolecules and aggregates, leading to toxicity and cell death. This study examines how overexpression of the N‐terminally truncated Tau35 protein affects proteolytic pathways, including autophagy and endo‐lysosomal processes.

**METHODS:**

Using the Tau35 mouse model and SH‐SY5Y human neuroblastoma cells stably expressing Tau35 or full‐length tau, we assessed protein degradation and lysosomal function via Western blotting, proteomics of lysosome‐enriched brain fractions, cathepsin activity assays, endocytosis/proteolysis assays, and live‐cell imaging using LysoTracker.

**RESULTS:**

We identified early endo‐lysosomal alterations associated with Tau35 expression, including increased endocytosis, disrupted autophagic flux, proteolytic impairment, and lysosomal motility defects.

**DISCUSSION:**

These findings extend previous research by elucidating Tau35‐induced dysfunction in intracellular degradation systems and offer mechanistic insight into tauopathy progression. This work provides a foundation for developing targeted therapies to restore acidification, proteostasis, and lysosomal function in tauopathies.

**Highlights:**

- **Tau35, an N‐terminally truncated tau fragment, disrupts proteolytic pathways**: We show that Tau35 overexpression leads to significant alterations in autophagy and endo‐lysosomal function.
- **Endo‐lysosomal dysfunction is an early pathological event**: Our findings demonstrate early‐stage increases in endocytosis, impaired proteolytic activity, altered autophagic flux, and disrupted lysosomal motility in Tau35‐expressing models.
- ***In vivo* and *in vitro* models confirm consistent pathogenic signatures**: Parallel studies in a Tau35 mouse model and SH‐SY5Y cells reveal converging cellular and molecular dysfunctions.
- **Lysosome‐enriched proteomics reveals novel pathway alterations**: Proteomic profiling of lysosomal fractions identifies Tau35‐specific protein dysregulation contributing to disease pathology.
- **Mechanistic insights into tauopathy progression**: These results provide a mechanistic understanding of how truncated tau species contribute to neuronal dysfunction, offering a rationale for targeting endo‐lysosomal pathways in therapeutic development.

## 1 BACKGROUND

Tauopathies are progressive neurodegenerative disorders characterized by the intracellular deposition of abnormal tau protein aggregates and age‐related neuronal loss.^1^ The accumulation of tau within cells reflects disruptions in protein homeostasis and impaired clearance mechanisms mediated by the endo‐lysosomal and autophagic pathways.^2^ Growing genetic and molecular evidence suggests that dysfunction in these systems extends beyond the degradation of aggregation‐prone proteins, implicating their broader roles in the pathophysiology of tauopathies and other neurodegenerative diseases.^2^, ^3^ Carriers of lysosomal gene mutations, such as GBA in Gaucher disease^4^ or GRN in neuronal ceroid lipofuscinosis,^5^ show elevated risks for Parkinson's and frontotemporal dementia, suggesting that lysosomal haploinsufficiency contributes to age‐related neurological decline. In Alzheimer's disease (AD), early endo‐lysosomal disruptions, such as enlarged endosomes, lysosome‐filled dystrophic neurites, and intracellular amyloid beta (Aβ) buildup, reflect lysosomal enzyme deficiencies, impaired autophagy, and proteostasis.^3^, ^6^ Genetic variants in lysosomal enzymes, including cathepsin D (CTSD),^7^ hexosaminidase B (HEXB),^8^ cathepsin B (CTSB), cathepsin H (CTSH), and glucocerebrosidase (GBA),^9^ modestly increase AD risk. These changes also impact tau: Lysosomal and endosomal dysfunction impedes tau clearance, enables tau fragmentation into aggregation‐prone forms via cathepsin L (CTSL)/cathepsin D (CTSD) cleavage, and supports tau propagation through exosomal and endosomal pathways.^10^, ^11^, ^12^ Taken together, these studies highlight how subtle disruptions in the lysosomal–autophagy network promote protein accumulation and neuronal vulnerability, revealing a shared pathogenic convergence between lysosomal storage disorders and neurodegenerative diseases.^13^

**RESEARCH IN CONTEXT**

1. **Systematic review**: Tauopathies are associated with disruptions in the ALP, a system crucial for protein homeostasis. While full‐length tau accumulation is known to impair ALP function, the specific impact of tau fragments, such as Tau35, remains less well understood.
2. **Interpretation**: This study demonstrates that Tau35 overexpression induces early lysosomal dysfunction, increasing endocytosis while impairing proteolysis, autophagic flux, and lysosomal motility. These findings suggest that even modest reductions in lysosomal function can permit pathogenic protein accumulation and cellular destabilization in tauopathies, underscoring shared mechanisms across neurodegenerative diseases and lysosomal storage disorders.
3. **Future directions**: By pinpointing distinct lysosomal defects associated with Tau35, our work provides a foundation for mechanistic exploration and supports the development of lysosome‐targeted therapeutic strategies. Future studies should assess whether similar endo‐lysosomal alterations occur in human tauopathy tissue and evaluate approaches to restore lysosomal efficiency in disease models.

While aberrant post‐translational modifications of tau, especially hyperphosphorylation, have traditionally been considered key drivers of tau aggregation and neurotoxicity,^14^ recent findings highlight the critical role of tau truncation in neurodegeneration.^15^ The process of tau proteolysis has attracted growing interest for its potential role in driving disease progression through mechanisms specific to individual fragments, such as aggregation and cell‐to‐cell propagation.^15^, ^16^ Among these, the Tau35 fragment, initially identified in human brain tissue and associated with primary human tauopathies,^17^, ^18^ has been shown to disrupt kinase activity, as well as lysosomal and synaptic functions in the Tau35 mouse model. Notably, these mice express the Tau35 fragment at relatively low levels compared to most other overexpressing animal models.^19^

The effect of Tau35 overexpression in Chinese hamster ovary (CHO) cells, primary cortical neurons, and its sub‐endogenous expression in Tau35 mice was recently reported, demonstrating a progressive accumulation of abnormally phosphorylated tau species across all models,^19^, ^20^, ^21^ accompanied by structural and functional synaptic alterations,^20^, ^22^ behavioral abnormalities,^19^ and disruptions involving critical elements of the three primary cellular protein degradation pathways: the proteasome, lysosomes, and autophagy.^19^, ^23^ In 14‐month‐old Tau35 mice, representing late stages of the disease, notable elevations in p62 and LC3‐I/II markers have been observed, accompanied by a decrease in active cathepsin D (CTSD), a critical lysosomal enzyme responsible for protein degradation (summarized in Table 1).^19^

**TABLE 1:** Summary of autophagy‐lysosomal changes reported in Tau35 mouse model at late disease stages (14 M).^9^

Autophagy	Tau35 14 months	↑p62, ↑LC3I, and ↑LC3II
Lysosome‐mediated degradation		↓Mature CTSD ↓Acetyl α‐tubulin

This study investigated the impact of Tau35 overexpression on physiological proteolytic pathways, with a focus on early alterations in autophagy and endo‐lysosomal processes. To achieve this, the Tau35 mouse model was utilized alongside the newly generated human neuroblastoma (SH‐SY5Y) cell lines stably expressing either the N‐terminally truncated 35 kDa human tau (187–441) with a C‐terminal HA tag (Tau35) or full‐length (2N4R) human tau with a C‐terminal Avi tag. Western blotting, cathepsin activity assays, and proteomic analysis of lysosome‐enriched brain fractions from Tau35 mice in early (4 month) and advanced (10 month) disease stages, as well as proteolysis and endocytosis assays, cathepsin activity assays, and LysoTracker‐based live‐cell imaging in differentiated SH‐SY5Y human neuroblastoma cells, were employed to assess the effects of Tau35 fragment overexpression on protein degradation pathways. This work extends prior research by uncovering early pathological events and highlighting the role of altered endocytosis, proteolytic dysfunction, impaired autophagic flux, and lysosomal motility disruptions in the presence of disease‐associated human tau fragments. Insights into the downstream neurotoxic mechanisms of tau alterations may inform the development of targeted therapeutic strategies.

## 2 METHODS

### 2.1 Mice

#### 2.1.1 Ethics statement

All experimental procedures adhered to the 1986 Animals (Scientific Procedures) Act and received approval from the local ethical review committee. The study was conducted in compliance with ARRIVE guidelines 2.0.^24^ Mice were generated via targeted knock‐in of the Tau35 cDNA construct at the Hprt locus on the X chromosome, under the regulation of the human tau promoter, as previously described.^19^ The construct encodes an N‐terminally truncated fragment of wild‐type (WT) human tau protein (amino acids 187–441) with a haemagglutinin (HA) tag appended to the C‐terminus. Male hemizygous transgenic and WT mice were used exclusively in this study to circumvent potential complications arising from incomplete X chromosome inactivation in female mice.

#### 2.1.2 Preparation of mouse brain homogenates for Western blots

Mice were euthanized by cervical dislocation, and brains were promptly extracted, snap‐frozen on dry ice, and stored at −80°C. Brain tissue was lysed through ultrasonication (parameters: 40% amplitude, 4‐s pulses, and 30‐s duration) using a Vibra‐Cell ultrasonic liquid processor (Model No. VCX 130, Sonics and Materials, Newton, CT, USA) in ice‐cold RIPA buffer (150 mM NaCl, 1 mM ethylenediaminetetraacetic acid [EDTA], 50 mM Tris‐HCl, 1% [v/v] NP‐40, 0.5% [w/v] sodium deoxycholate, 0.1% [w/v] sodium dodecyl sulfate [SDS]) supplemented with protease (cOmplete, EDTA‐free, Merck Millipore, Catalog No.: 11873580001) and phosphatase (PhosSTOP, Sigma‐Aldrich, Catalog No.: 4906845001) inhibitors. This process was performed in a refrigerated chamber and repeated three times, with incubation on ice for 3 min between cycles. The lysates were centrifuged at 10,000 × g for 15 min at 4°C, and the resulting supernatants were stored at −80°C for further analysis.

#### 2.1.3 Isolation of lysosome enriched fractions from mouse brain tissue

Mice were sacrificed by cervical dislocation, and their brains were promptly extracted, snap‐frozen on dry ice, and stored at −80°C. Lysosome‐enriched fractions were isolated from Tau35 and WT control mouse brain tissue using the Lysosome Enrichment Kit for Tissue and Cultured Cells (Thermo Fisher Scientific, Catalog No.: 89839) following the manufacturer's instructions. Briefly, 500 mg of brain tissue per sample was washed with 1× phosphate‐buffered saline (PBS), minced, and homogenized in 1 mL of Lysosome Enrichment Reagent A using a glass Dounce homogenizer. Next, 1 mL of Lysosome Enrichment Reagent B was added, and the mixture was inverted five to six times for thorough mixing. All steps were conducted in a refrigerated chamber and performed on ice. Samples were then centrifuged at 500 × g for 10 min at 4°C, and the supernatants were collected and kept on ice. In a 6.3‐mL quick‐seal polypropylene ultracentrifuge tube (Beckman Coulter Inc., Catalog No.: 345830), OptiPrep (Iodixanol) gradients were prepared in descending concentrations: 30%, 27%, 23%, 20%, and 17%. The sample was diluted in 15% OptiPrep medium and overlaid on top of the density gradients. After ultracentrifugation at 145,000 × g for 2 h at 4°C, the lysosome pellet was washed in two to three volumes of 1xPBS to reduce the OptiPrep media concentration and collected by adding 1 mL of Gradient Dilution Buffer. Lysosome purity was evaluated by Western blotting using antibodies against the lysosomal marker LAMP2. Samples were stored at −80°C in lysosomal buffer (2% CHAPS [3‐[(3‐cholamidopropyl)dimethylammonio]‐1‐propanesulfonate] in 1× Tris Buffered Saline [TBS]) for subsequent analysis.

#### 2.1.4 Preparation of lysosome‐enriched fractions for proteomic analysis

Sample lysis, reduction, alkylation, and enzymatic digestion were performed before peptide purification. The protein concentration of lysosome‐enriched fractions from transgenic and control mouse brain samples was measured using the Bradford Protein Assay (Pierce, Thermo Fisher Scientific, Catalog No.: 23200), and 12 µg of protein were used for each replicate. To improve protein separation, 8 M urea and 5 mM Dithiothreitol (DTT) were added, followed by incubation at 37°C, in a thermomixer (750 rpm) for 30 min. To alkylate free cysteines, iodoacetamide (IAA) was added to a final concentration of 20 mM followed by vortexing and sample incubation at room temperature in the dark for 20 min. Proteins were then precipitated by the addition of methanol/chloroform/18 MΩ water (4:1:3), vigorous vortexing, and centrifugation at 14,000 rpm for 1 min. The liquid phases were discarded and the protein pellets were washed once with methanol and centrifuged at 14,000 rpm for 1 min, followed by removal of methanol. Protein pellets were then air‐dried and solubilized in 0.2 M EPPS (4‐(2‐Hydroxyethyl)‐1‐ piperazinepropanesulfonic acid, 4‐(2‐Hydroxyethyl) piperazine‐1‐propanesulfonic acid, N‐(2‐hydroxyethyl)piperazine‐Nʹ‐(3‐propanesulfonic acid)) buffer. Proteins were digested by the addition of trypsin (0.5 µg, Thermo Fisher Scientific, Catalog No.: 90057), rigorous vortexing, and overnight incubation in a thermomixer (750 rpm, ThermoMixer C, Eppendorf SE). The following day, samples were dried to completion in a Speedvac (Thermo Fisher Scientific, IL, USA), and peptides were cleaned up using C18 spin columns (Thermo Fisher Scientific, Catalog No.: 89852) according to the manufacturer's instructions. Briefly, samples were resuspended in 300 µL of 0.1% trifluoroacetic acid (TFA) and eluted in 50% acetonitrile (ACN)/0.1% TFA. Following elution, samples were dried to completion by Speedvac and stored at −80°C.

#### 2.1.5 Proteomic analysis

##### Liquid chromatography with tandem mass spectrometry (LC‐MS/MS)

The extracted peptide samples were individually resuspended in MS sample buffer (2% [ACN] in 0.05% formic acid [FA]) to a concentration of 1 mg/mL, 6 µL of which was injected to be analyzed by LC‐MS/MS. Chromatographic separation was performed using a U3000 UHPLC NanoLC system (Thermo Fisher Scientific, UK). Peptides were resolved by reverse phase chromatography on a 75‐µm C18 Pepmap column (50 cm length) using a three‐step linear gradient of 80% ACN in 0.1% FA. The gradient was delivered to elute the peptides at a flow rate of 250 nL/min over 60 min starting at 5% B (0 to 5 min) and increasing solvent to 40% B (5 to 40 min) prior to a wash step at 99% B (40 to 45 min) followed by an equilibration step at 5% B (45 to 60 min).

The eluate was ionized by electrospray ionization using an Orbitrap Fusion Lumos (Thermo Fisher Scientific, UK) operating under Xcalibur version 4.3. The instrument was first programmed to acquire using an Orbitrap‐Ion Trap method by defining a 3‐s cycle time between a full MS scan and MS/MS fragmentation by collision‐induced dissociation. Orbitrap spectra (FTMS1) were collected at a resolution of 120,000 over a scan range of m/z 375 to 1800 with an automatic gain control (AGC) setting of 4.0 × 10⁵ (100%) with a maximum injection time of 35 ms. Monoisotopic precursor ions were filtered using charge state (+2 to +7) with an intensity threshold set between 5.0 × 10^3^ and 1.0 × 10^2^ ^0^ and a dynamic exclusion window of 35 s and ± 10 ppm. MS2 precursor ions were isolated in the quadrupole set to a mass width filter of 1.6 m/z. Ion trap fragmentation spectra (ITMS2) were collected with an AGC target setting of 1.0 × 10⁴ (100%) with a maximum injection time of 35 ms with CID collision energy set at 35%.

#### 2.1.6 Raw proteomic data processing and analysis

Raw MS data were processed into peak list files using Proteome Discoverer (Thermo Scientific; version 2.5). The raw data file was searched using the Sequest^25^ search algorithm against the Uniprot Mouse Taxonomy database (37,716 entries). Database searching was performed at a stringency of 1% false discovery rate (FDR), including a decoy search. Post‐translational modifications for carbamidomethylation (C, static), oxidation (M, variable), and phosphorylation (S, T, and Y; variable) were included in the database search. Protein/peptide identification, along with peak intensities, were exported as Excel files for subsequent analysis using R. The raw proteome data (intensity values) were log_2_;‐transformed and converted into an expression set object (iBAQ values). Differential expression analysis at the peptide level was conducted using R (R Core Team, 2023. R: A Language and Environment for Statistical Computing. R Foundation for Statistical Computing, Vienna, Austria. https://www.R‐project.org/), RStudio (version: 2024.12.0 + 467) (Posit team, 2024. RStudio: Integrated Development Environment for R. Posit Software, PBC, Boston, MA, USA. URL http://www.posit.co/) and the limma package,^26^, ^27^ which employs empirical Bayes methods to generate accurate variance estimates, even with a small sample size. Lists of differentially expressed proteins were generated following stringent (*p* value: *p*_cutoff ← 0.05, fold change: fc_cutoff ← 1) and lenient (*p* value: *p*_cutoff ← 0.05, fold change: fc_cutoff ← 0.5) criteria (discovery analysis) and used the Cluster Profiler package^28^ to perform Gene Ontology (GO) analysis in R, aiming to identify the biological processes and molecular functions that are impacted in lysosome‐enriched fractions. The complete set of detected proteins, along with the differentially expressed proteins identified via discovery analysis using the limma package, was also cross‐referenced with a curated list of autophagy and endo‐lysosomal pathway‐associated proteins,^29^ with the aim of pinpointing specific autophagy‐lysosomal pathway (ALP) components exhibiting differential expression in Tau35 brains. The MS proteomics data have been deposited to the ProteomeXchange Consortium via the PRIDE^30^ partner repository with the dataset identifier PXD062128 and https://doi.org/10.6019/PXD062128.

### 2.2 Western blots

Protein concentrations were measured using the bicinchoninic acid (BCA) protein assay following the manufacturer's protocol (Pierce BCA Protein Assay Kit, Thermo Fisher Scientific, Catalog No.: 23225). Samples prepared in NuPAGE LDS Sample Buffer (4×) (Thermo Fisher Scientific, Catalog No.: NP0007) were heated at 95°C for 10 min and resolved using Bolt Bis‐Tris Plus Mini Protein Gels (4% to 12%, 1.0 mm, WedgeWell format; Thermo Fisher Scientific, Catalog No.: NW04125BOX or NW04127BOX). Proteins were transferred onto nitrocellulose membranes (Amersham Protran 0.45 NC, Cytiva, Catalog No.: 10600007) and blocked with Intercept (TBS) Blocking Buffer (LI‐COR Biosciences, Catalog No.: 927‐60001). Membranes were incubated with primary antibodies overnight at 4°C, washed with TBS containing 0.02% (v/v) Tween‐20, and treated with fluorophore‐conjugated secondary antibodies for antigen detection. Imaging was performed using the Odyssey imaging system (LI‐COR Biosciences), and ImageStudio Lite software (LI‐COR Biosciences) was used for Western blot quantification.

### 2.3 Human neuroblastoma SH‐SY5Y cell lines

#### 2.3.1 Cell culture

Human neuroblastoma SH‐SY5Y cells were sourced from the American Type Culture Collection (ATCC, passage 16) and cultured in DMEM/F12 (Dulbecco's Modified Eagle Medium/Nutrient Mixture F‐12) GlutaMAX (Thermo Fisher Scientific, Catalog No.: 31331‐028) supplemented with 10% heat‐inactivated serum (Gibco, Catalog No.: 16000044), and 1% penicillin‐streptomycin (Gibco, Catalog No.: 15140922). Cells were cultivated in T75 flasks, maintained at 37°C in a humidified incubator with 5% CO_2_, and kept below ATCC passage + 3 to avoid cell senescence.

#### 2.3.2 Cloning and expression of fusion proteins in SH‐SY5Y cells

The codon‐optimized synthetic gene sequences (Tau35‐HA, Tau35‐mKO_2_, mKO_2_‐Tau35, Avi‐FL tau, FL tau‐eGFP, and eGFP‐FL tau) were generated by GenScript and subsequently cloned into the pLVX‐TetOne‐Puro vector. All constructs were confirmed through restriction enzyme digestion and sequencing. Low passage, undifferentiated SH‐SY5Y cells were transiently transfected with each of the newly generated plasmids using Lipofectamine 3000 reagent (Thermo Fisher Scientific, Catalog No.: L3000015) following the manufacturer's protocol.

#### 2.3.3 Cell transfection, clonal isolation, and stable SH‐SY5Y cell line production

Human neuroblastoma SH‐SY5Y cells were cultured in six‐well plates (1 × 10⁴ cells/well) and maintained until they reached 70% to 80% confluence. At this point, the cells were transfected with the plasmids of interest (pLVX‐TetOne‐Puro—[Tau35‐HA, Tau35‐mKO_2_, mKO_2_‐Tau35, Avi‐FL tau, FL tau‐eGFP, and eGFP‐FL tau]) generated by GenScript or pLVX‐TetOne‐Puro‐GFP (Addgene plasmid no. 171123; Addgene_171123)^31^ using Lipofectamine 3000 reagent (Thermo Fisher Scientific, Catalog No.: L3000015) according to manufacturer's instructions. Approximately 48 h after transfection, puromycin dihydrochloride (2 µg/mL, Thermo Fisher Scientific, Catalog No.: A1113803) was added to the culture medium to initiate selection. The medium was then replaced every 2 to 3 days with fresh selection medium containing 2 µg/mL puromycin for up to 2 weeks to ensure selection. After selection with puromycin, stable cell pools were cultured in the presence of 1 µg/mL doxycycline hyclate (Sigma‐Aldrich, Catalog No.: D5207) for 5 days to induce construct expression. Colony isolation was performed as follows: Colonies of interest were marked on the underside of the culture plate. The tissue culture medium was carefully aspirated, and the plate was washed with Dulbecco's PBS (DPBS – no calcium, no magnesium, Thermo Fisher Scientific, Catalog No.: D5207). Sterile cloning discs (SP Bel‐Art, Catalog No.: F37847‐0001) were dipped in trypsin solution (Trypsin 0.25% EDTA, Thermo Fisher Scientific, Catalog No.: 25200072) and placed onto the marked colonies. The plate was incubated for 3 to 10 min to ensure complete trypsinization of the colonies. Meanwhile, a 24‐well plate containing 1 mL of growth medium supplemented with puromycin (1.5 µg/mL) and doxycycline (1 µg/mL) was prepared for colony transfer. After trypsinization, the cloning discs were removed and transferred to the prepared 24‐well plate. The cells adhered to the discs and maintained in individual wells. Once the cells reached near confluence, they were trypsinized and passaged into larger culture vessels for further expansion and characterization. The cloning discs were left in the 24‐well plate for subsequent monitoring. To assess colony quality, one well of a six‐well plate was used for routine passaging, while a second well of a 24‐well plate, containing a 13‐mm coverslip, was used for colony screening. The coverslips were fixed after 1 to 2 days to evaluate colony morphology and assess whether the colonies should be retained or subjected to further subcloning to achieve >95% homogeneity.

For those colonies of interest, cells were passaged from the six‐well plate to at least three separate dishes: one for cryopreservation as passage 0, one for continued culture (if the cell line was already clonal), and one for subcloning (if necessary). Subcloning was performed by seeding the cells at clonal density. Once a desired clone or subclone was identified, a reduced concentration of puromycin (1 µg/mL) was used for routine maintenance. Following selection, clones were cultured in the presence of 1 µg/mL doxycycline and 1 µg/mL puromycin for 5 days, and expression was analyzed by Western blotting to evaluate expression levels and confirm that fusion proteins were full length and not degraded when overexpressed. In brief, cells were lysed in RIPA Lysis and Extraction buffer (89900, Thermo Fisher Scientific) supplemented with protease (cOmplete, EDTA‐free, Merck Millipore, Catalog No.: 11873580001) and phosphatase (PhosSTOP, Sigma‐Aldrich, Catalog No.: 4906845001) inhibitors. Protein levels were assessed by the BCA protein assay, according to the manufacturer's instructions (Pierce BCA Protein Assay Kit, Thermo Fisher Scientific, Catalog No.: 23227). Lysates were boiled in NuPAGE LDS Sample Buffer (4×) and processed as described above.

#### 2.3.4 SH‐SY5Y cell line differentiation

SH‐SY5Y cell lines were re‐cultured, expanded for two to three passages, and then differentiated according to the Shipley et al.^32^ protocol, with minor modifications, including the replacement of retinoic acid with the synthetic retinoid EC23 (Sigma‐Aldrich, Catalog No.: SML2404). Stock solutions of the synthetic retinoid EC23 were prepared by dissolving EC23 in DMSO to a final concentration of 5–10 mM. Aliquots of the stock solutions are stored at −20°C, protected from light. The detailed media formulations are listed in Table 2.

**TABLE 2:** Media recipes for SH‐SY5Y differentiation.

Basic growth media		
Component	For 25 mL	Dilution
DMEM/F‐12	Up to 25 mL	
10% FBS	2.5 mL	
1× Pen/Strep	250 µL	1:100

Differentiation medium no. 1		
Component	For 25 mL	Dilution
DMEM/F‐12	Up to 25 mL	
2.5% FBS	625 µL	
1× Pen/Strep	250 µL	1:100
10 µM EC23	10 µL/5 µL (5 mM stock)/(10 mM stock)	1:500/1:1000

Differentiation medium no. 2		
Component		Dilution
DMEM/F‐12	Up to 25 mL	
1% FBS	250 µL	
1× Pen/Strep	250 µL	1:100
10 µM EC23	10 µL/5 µL (5 mM stock)/(10 mM stock)	1:500/1:1000

Differentiation medium no. 3		
Component		Dilution
Neurobasal	Up to 25 mL	
1× B‐27	500 µL (50× stock)	1:50
20 mM KCL	250 µL (1 M stock)	1:50
1× Pen/Strep	250 µL	1:100
2 mM glutamax	250 µL (100× stock)	1:100
50 ng/mL BDNF	12.5 µL (10 µg/mL stock)	1:200
2 mM dibutyryl cyclic AMP (db‐cAMP)	250 µL (0.2 M stock)	1:500
10 µM EC23	10 /5 µL (5 mM stock)/(10 mM stock)	1:500/1:1000

Abbreviations: BDNF, brain‐derived neurotrophic factor; FBS, fetal bovine serum.

*Add EC23 immediately prior to use.

**Once EC23 is added, keep medium no more than 3 days.

The differentiation process spanned up to 20 days and involved gradual serum starvation followed by the introduction of neurotrophic factors (detailed factor information in Table 2). On day 0, 25,000 to 100,000 cells were plated onto uncoated 35‐mm dishes, or 50,000 to 100,000 cells per well in six‐well plates. For 96‐well plates, 4000 to 5000 cells were seeded per well. These ranges are indicative, with the final cell number determined by the experimental design. Differentiation began on day 1 with the replacement of basic growth medium by differentiation medium no. 1, which was refreshed on days 1, 3, and 5. On day 7, cells were split 1:1 and replated onto uncoated dishes or plates or Ibidi chambers in differentiation medium no. 1. On day 8, differentiation medium no. 2 was introduced and replaced again on day 10. On day 11, cells were split 1:1 and plated onto ECM (MAXGEL ECM Mixture, Merck, Catalog No.: E0282)‐coated dishes, plates, or Ibidi chambers in differentiation medium no. 2. Beginning on day 12, the culture medium was replaced with differentiation medium no. 3 and supplemented with 1 µg/mL doxycycline and 1 µg/mL puromycin to induce transgene expression. Fully differentiated SH‐SY5Y cells were utilized on day 14 for Western blot, qPCR, immunocytochemistry, and live cell imaging experiments.

#### 2.3.5 Antibodies and reagents

The following primary antibodies were used in this study: rabbit anti‐tau (Agilent, Catalog No.: A0024, Western blot, 1:5000, RRID: AB_10013724), mouse anti‐GAPDH (6C5) (Santa Cruz Biotechnology, Catalog No.: sc‐32233, Western blot, 1:5000, RRID: AB_627679), rabbit anti‐MCU (D2Z3B) (Cell Signaling Technology, Catalog No.: 14997, Western blot, 1:1000, RRID: AB_2721812), goat anti‐CTSB (R and D Systems, Catalog No.: AF953, Western blot, 1:1000, RRID: AB_355738), goat anti‐CTSB (R and D Systems, Catalog No.: AF1029, Western blot, 1:1000, RRID: AB_2087094), goat anti‐CTSL (Novus, Catalog No.: AF1515, Western blot, 1:1000, RRID: AB_2665930), mouse anti‐p62/SQSTM1 (2C11) (Novus, Catalog No.: H00008878‐M01, Western blot, 1:2000, RRID: AB_548364), rabbit anti‐LAMP1 (Thermo Fisher Scientific, Catalog No.: PA1‐654A, Western blot, 1:1000, RRID: AB_2134611), rabbit anti‐LAMP2 (Thermo Fisher Scientific, Catalog No.: PA1‐655, Western blot, 1:1000, RRID: AB_2134625), and anti‐ATP6V1E1 (Proteintech, Catalog No.: 15280‐1‐AP, Western blot, 1:1000, RRID: AB_2062545). For Western blots secondary antibodies were purchased from LI‐COR Biosciences and used at 1:10,000 dilution.

The following additional reagents were used in this study: DPBS‐no calcium, no magnesium (Thermo Fisher Scientific, Catalog No.: 14190094), KCL 2 M RNase free (Invitrogen, Catalog No.: 10606365), Neurobasal (without phenol red [Thermo Fisher Scientific, Catalog No.: 12348‐017]), B27 supplement (Gibco, Catalog No.: 17504‐044), dibutyryl‐cAMP (db‐cAMP) (Insight Biotechnology Limited, Catalog No.: sc‐201567A), human BDNF (Cambridge Bioscience, Catalog No.: GFH1‐10), CHAPS (Scientific Laboratory Supplies, Catalog No.: C3023‐1G), DTT (Merck, Catalog No.: 10197777001), 3‐(4‐[2‐hydroxyethyl]‐1‐piperazinyl) propanesulfonic acid (EPPS) (Thermo Fisher Scientific, Catalog No.: A13714.14), ACN (Merck, Catalog No.: 360457), FM (Thermo Fisher Scientific, Catalog No.: 10559570), TFA (Merck, Catalog No.: 8082600026), IAA (Merck, Catalog No.: I1149).

#### 2.3.6 BSA (bovine serum albumin) and DQ (dye‐quenched)‐BSA experiments

Control, Tau35‐HA, and Avi‐FL tau SH‐SY5Y lines were plated on 96‐well plates (Falcon, Corning, Catalog No.: 353219), and on day 14, differentiated cells were treated with 10 µg/mL red DQ‐Red BSA (Thermo Fisher Scientific, Catalog No.: D12051) and 50 µg/mL Alexa‐488 BSA (Thermo Fisher Scientific, Catalog No.: A13100) for 4 h in a humidified incubator. Following the incubation, cells were washed three times with warm culture medium and counterstained with NucBlue Live ReadyProbes Reagent (Invitrogen, Catalog No.: R37605) to visualize, count, and segment nuclei. The cells were then washed with DPBS and fixed in 2% (w/v) paraformaldehyde for 10 min. Imaging was performed using a spinning disk confocal microscope (Opera Phenix HCS System, PerkinElmer). Images were acquired using a 40× water‐immersion objective (NA 1.1) with a binning factor of two. Images were captured sequentially at each wavelength, utilizing a 405‐nm laser for NucBlue, a 488‐nm laser for Alexa‐488 BSA, and a 568‐nm laser for DQ‐Red BSA. To encompass the entire cell depth, 10‐µm z‐stacks were acquired with 0.4‐µm intervals. Data analyses were conducted using PerkinElmer's Harmony HCA software.

#### 2.3.7 Pharmacological treatments to assess autophagy dynamics

Pharmacological modulators were applied to control, Tau35‐HA, and Avi‐FL tau SH‐SY5Y lines in order to assess autophagic dynamics. Cells were treated with 300 nM Bafilomycin A1 (BafA1) (Sigma‐Aldrich, Catalog No.: B1793‐10 µg) for 6 h or 100 mM chloroquine (CQ) (Sigma‐Aldrich, Catalog No.: C6628‐25 g) for 3 h to inhibit autophagy and disrupt endo‐lysosomal acidification. To induce autophagy through mTOR inhibition, cells were exposed to 1 µM Torin 1 (Sigma‐Aldrich, Catalog No.: 475991‐10 mg) for 3 h or 300 nM AZD8055 (provided by AstraZeneca) for 3 h. DMSO‐treated cells served as vehicle controls.

#### 2.3.8 LysoTracker staining and imaging

Control, Tau35‐HA, and Avi‐FL tau SH‐SY5Y lines were plated on eight‐well chamber slides (Ibidi, Catalog No.: 80841) and, on day 14, differentiated cells were treated with 100 nM LysoTracker Deep Red (Thermo Fisher Scientific, Catalog No.: L12492) for 1 h in a humidified incubator, according to the manufacturer's instructions. Following the incubation, cells were washed three times with warm culture medium and counterstained with NucBlue Live Ready Probes Reagent (Invitrogen, Catalog No.: R37605) to visualize, count, and segment nuclei. Warm imaging medium (Live Cell Imaging Solution, Invitrogen, Catalog No.: 12363603) was added and cells were returned to the incubator for 10 min before imaging. Live cell imaging microscopy was performed in imaging medium at 37°C with humidified CO_2_ (Okolab incubator with CO_2_ control) using the Vt‐iSIM super‐resolution microscope with Hamamatsu Flash 4.0 sCMOS camera. Images were acquired using a 100× silicone‐immersion objective (NA 1.35). Prior to image acquisition the lens was calibrated as previously described^33^ to minimize uneven background and non‐specific labeling, ensuring an optimal signal‐to‐background (SBR) ratio. Staining with LysoTracker‐647 allowed clear visualization of lysosome morphology in live‐cell SIM images, with minimal fluorescence background. To assist with nuclear detection and segmentation, cells were counterstained with NucBlue reagent. For LysoTracker spot detection, intensity and motility parameter analysis, 100 frames were acquired per imaging session (10 s), at a fast acquisition mode, at a rate of 100 frames per second (fps), utilizing a 640‐nm laser for LysoTracker Deep Red. Following video acquisition, an overview image was captured sequentially at each wavelength, utilizing a 640‐nm laser for LysoTracker Deep Red and a 405‐nm laser for NucBlue and brightfield. Acquisition was controlled and data stored using NIS‐Elements version 5.2 (Nikon). Data were analyzed using NIS‐Elements Advanced Research software (Nikon, RRID:SCR_014329), ImageJ,^34^ and the Python programming language (Python Software Foundation, https://www.python.org/, Van Rossum, G. & Drake Jr, F. L., 1995) reference manual (Centrum voor Wiskunde en Informatica Amsterdam). The analysis protocol began by analyzing video files acquired at the 647‐nm wavelength with NIS Elements. The DAPI and brightfield channels from corresponding overview images were transferred to these video files to provide additional context. Subsequently, the far‐red channel (647 nm) was selected for processing, followed by preprocessing using median (radius = 2) and rolling ball (radius = 5) filters to reduce noise. The bright spot detection (diameter = 0.6 µm) algorithm (NIS‐Elements AR 6.02.01) was used to detect bright spots, which were used to generate binary objects for further analysis. Regions of interest (ROIs) corresponding to individual cells were delineated based on the brightfield channel. Quantitative measurements included total object (spot) count, number of spots per cell, total area, mean intensity of objects (spots), total cell area, and motility parameters such as distance traveled, straightness, mean velocity, and mean square displacement. The exported .csv files were subsequently uploaded to Python for advanced statistical analysis and visualization. This included the generation of raincloud plots to illustrate the distribution of quantitative data and rose plots to represent directional motility patterns of lysosomes. This comprehensive approach enabled detailed analysis of lysosomal behavior across all cell lines.

### 2.4 Cathepsin activity assays

Cathepsin B and cathepsin D activity was measured using commercial fluorometric kits (Abcam, cathepsin B: Catalog No.: ab65300 and cathepsin D: Catalog No.: ab65302). Tissue or cell samples were homogenized in lysosome enrichment buffer (according to the manufacturer's instructions; Thermo Fisher Scientific, Catalog No.: 89839) or the respective lysis buffer, incubated on ice for 10 to 30 min, and centrifuged at 15,000 × g for 10 min. Protein concentrations were determined by BCA assay (Pierce BCA Kit, Thermo Fisher Scientific, Catalog No.: 23225). For each assay, 100 µg protein was diluted in 50 µL of the supplied cathepsin B or cathepsin D lysis buffer and loaded into black, clear‐bottom 96‐well plates (Revvity PhenoPlate, Catalog No.: 6055300). Negative controls included the cathepsin B inhibitor provided in the ab65300 kit and Pepstatin A (Apollo Scientific, Catalog No.: BIMI2205) for cathepsin D. Substrates were Ac‐RR‐AFC for cathepsin B and GKPILFFRLK(Dnp)‐D‐R‐NH_2_‐MCA for cathepsin D. Plates were incubated at 37°C in the dark for 120 min, and fluorescence was recorded on a ClarioStar plate reader (BMG LABTECH) at excitation/emission 400/505 nm (cathepsin B) or 328/460 nm (cathepsin D). Activity was calculated as blank‐corrected fluorescence normalized to incubation time and protein amount, expressed as relative fluorescence units per minute per milligram (RFU/min/mg). To control for non‐specific activity, an inhibited control was included per preparation. Each sample was measured in duplicate, and biological replicates were used as the experimental unit.

### 2.5 Quantification and statistical analysis

For all datasets the statistical significance was assessed as follows: For two group comparisons, two‐tailed unpaired Student's *t* tests were used to estimate statistical significance between means; for three group comparisons, the ordinary one‐way ANOVA test was used to estimate statistical significance between means of parametric datasets, and the Kruskal‐Wallis test was used to estimate statistical significance between means of not normally distributed datasets. Where appropriate, post hoc pairwise comparisons were conducted using Dunn's test with Bonferroni correction. Dataset normality was tested with either the Shapiro‐Wilk test (for small *n* numbers) or the Kolmogorov‐Smirnov test (for large *n* numbers). For drug treatment analyses, comparisons were performed using two‐way ANOVA, with group (three levels) and drug treatment (five levels) as independent factors. These analyses assessed main effects and potential interactions between group and treatment. Statistical analyses were performed using Prism (GraphPad, version 10) and R (dplyr, ggplot, tidyr, stats, and limma) or Python (pandas, seaborn, scipy, scikit, and scikit‐posthocs) packages. Sample number, number of experiments and statistical information are stated in the corresponding figure legends. In figures, asterisks denote statistical significance as follows: *p < 0.05, **p < 0.01, ***p < 0.001, ****p < 0.0001. Error bars represent the standard error of the mean (SEM).

## 3 RESULTS

### 3.1 Reduced LAMP2 expression and altered balance of active CTSB (decreased) and CTSL (increased) in the brains of Tau35 mice

**FIGURE 1:**
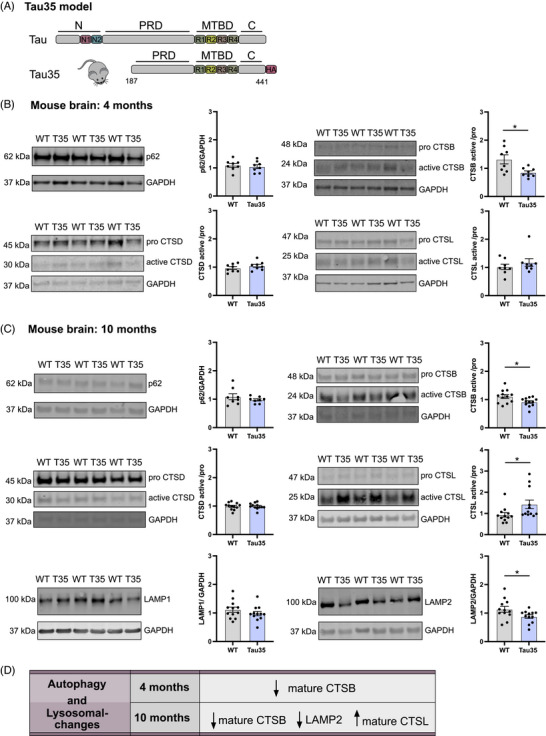
Reduced LAMP2 expression and imbalanced active cathepsins B/L in Tau35 mouse brains. (A) Schematic representation of Tau35‐HA construct that is expressed in Tau35 mice, in comparison to full‐length human tau (441 amino acids). (B and C) Western blots of total brain homogenates from WT and Tau35 mice aged 4 (B) and 10 (C) months, respectively, were probed with antibodies to p62, CTSB, CTSD, CTSL, LAMP1, LAMP2, and GAPDH. Quantification of the blots is shown in the graphs as mean ± SEM, *n* = 8–12 brains per group. Student's *t* test, ^*^ *p* < 0.05. (D) Table summarizing changes in key lysosomal and autophagy markers (p62, LAMP1, LAMP2, CTSB, CTSD, and CTSL) at early (4 M) and advanced (10 months) disease stages in Tau35 mice. Panel (C) represents the carboxy‐terminal domain; CTSB, cathepsin B; CTSD, cathepsin D; CTSL, cathepsin L; GAPDH, glyceraldehyde 3‐phosphate dehydrogenase; LAMP1, lysosomal‐associated membrane protein 1; LAMP2, lysosomal‐associated membrane protein 2; N, amino‐terminal domain; N1, N2, two amino‐terminal inserts, p62/SQSTM1, sequestosome‐1; PRD, proline‐rich domain; R1‐R4, four microtubule‐binding domain repeats; SEM, standard error of the mean; WT, wild type.

Tau35 mice, illustrated in Figure 1A with a schematic comparing the expressed protein to full‐length human tau, serve as a model for primary tauopathies.^19^ To investigate the impact of Tau35 expression on alterations in the ALP during disease progression, we euthanized mice at early (4 months; WT, *n* = 8; Tau35, *n* = 8) and advanced (10 months; WT, *n* = 8 to 12; Tau35, *n* = 8 to 12) pathological stages. The brains of these mice, which exhibited progressive tauopathy characterized by elevated tau phosphorylation, synaptic alterations,^20^ accumulation of abnormal tau species, cognitive and motor impairments, and reduced lifespan,^19^ were analyzed for the expression of selected lysosomal markers.

Western blot analysis of whole‐brain tissue revealed a significant reduction in the expression of mature cathepsin B (CTSB) at early disease stages (4 months) (Figure 1B), while no significant changes were observed in other tested cathepsins, including their mature and immature forms, or in other key autophagy and lysosomal markers (Figures 1B and S1A). At advanced disease stages (10 months), the reduction in mature CTSB expression persisted, accompanied by a decrease in the lysosomal marker LAMP2 and a significant increase in the expression of mature cathepsin L (CTSL). No significant changes were observed in the expression of cathepsin D (CTSD), LAMP1, p62, or the premature forms of any tested cathepsins (Figure 1C, Figure S1B).

These findings indicate that in Tau35 mice, early‐stage pathology is associated with a selective reduction in mature CTSB expression, while other markers remain unchanged. At advanced stages, reductions in both LAMP2 and mature CTSB are observed, alongside an increase in mature CTSL (Figure 1D), potentially reflecting a compensatory mechanism to mitigate the effects of CTSB deficiency and maintain tissue integrity.^35^

### 3.2 Early Tau35 pathology alters endo‐lysosomal pathways involved in mitochondria and energy/metabolism dynamics

The endo‐lysosomal system is essential for executing metabolic tasks, including the uptake, intracellular trafficking, processing, appropriation, degradation, and disposal of molecules. In the context of tauopathies and other neurodegenerative diseases, it orchestrates the internalization, trafficking, and clearance of aggregated proteins.^2^, ^36^ Given its critical role in maintaining cellular homeostasis, the endo‐lysosomal system is proposed to be a key regulator of neurodegeneration progression.

**FIGURE 2:**
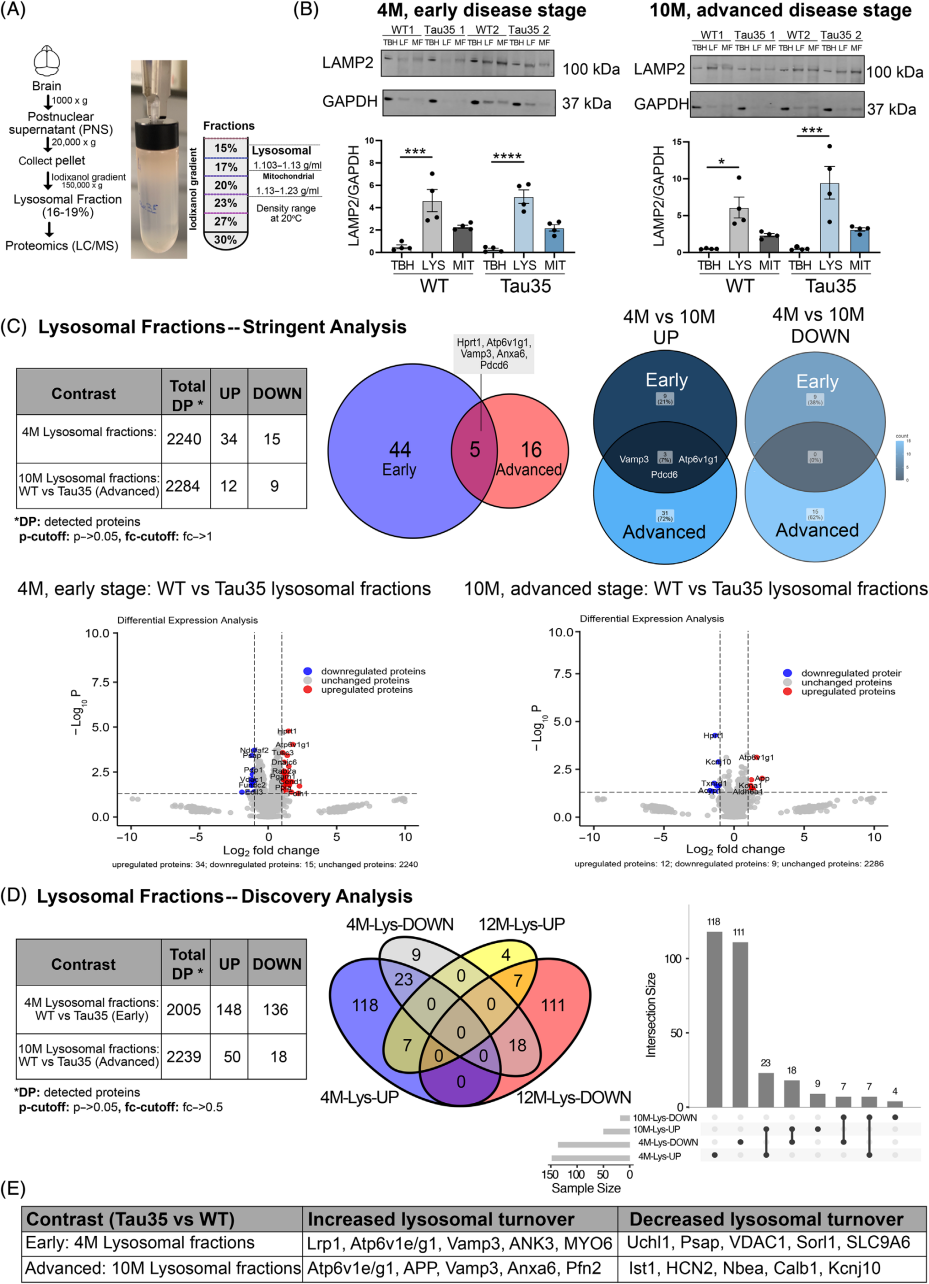
Lysosomal proteome alterations in Tau35 mouse brains during disease progression. (A) Schematic diagram of applied workflow for subcellular fractionation of lysosomes from mouse brain. Representative images of discontinuous iodixanol gradient showing enriched fractions from WT and Tau35 mouse brain samples. The positions of the lysosomal and mitochondrial fractions (MFs) within the gradient are marked, with the lysosome‐enriched fraction selected for subsequent experiments. (B) Western blots of total brain homogenates (TBHs), lysosomal fractions (LFs), and MFs from WT and Tau35 mice aged 4 and 10 months, respectively, were probed with antibodies to LAMP2 and GAPDH. Western blotting analysis of mouse brain extracts and the different subcellular fractions from WT and Tau35 brain reveal the enrichment of the lysosomal marker protein LAMP2 in LFs. Quantification of the blots is shown in the graphs as mean ± SEM, *n* = 4 brains per group. Ordinary one‐way ANOVA, *p < 0.05, ***p < 0.001, ****p < 0.0001. (C) The limma package in R was used to analyze differentially expressed proteins and produce volcano plots (stringent analysis: *p*‐cutoff: *p* → 0.05, fc‐cutoff: fc → 1) from early (4 months) and advanced (10 months) sample cohorts. Table summarizing the differentially expressed proteins between early and advanced cohorts of WT and Tau35 mice. Venn diagrams from the two cohorts illustrate distinct protein alterations specific to each disease stage. Five proteins were significantly altered in both cohorts (Vamp3, Hprt1, Atp6v1g1, Anxa6, and Pdcd6), three of which (Vamp3, Atp6v1g1, and Pdcd6) exhibit upregulated lysosomal processing in Tau35 samples. Volcano plots show log‐fold change (Log_2_FC) on the *x*‐axis, and −log_10_ transformed *p* values, adjusted for multiple testing using the Benjamini–Hochberg procedure on the *y*‐axis. (D) The limma package in R was used to analyze differentially expressed proteins (discovery analysis: *p*‐cutoff: *p* → 0.05, fc‐cutoff: fc → 0.5) from early and advanced sample cohorts. Table summarizing differentially expressed proteins between two cohorts, early and advanced, of WT and transgenic Tau35 mice. The upset plot is an interactive Venn diagram that shows the overlap of the four datasets (4 M‐Lys‐UP, 4 M‐Lys‐DOWN, 10 M‐Lys‐UP, and 10 M‐Lys‐DOWN) by arranging them as bar charts of their frequencies. The heights of the vertical bars correspond to intersection size – the number of differentially expressed proteins that are shared among the corresponding datasets. For example, the first vertical gray bar shows that the “4 M‐Lys‐UP” dataset contains 118 unique differentially expressed proteins, while the fifth vertical gray bar shows that the “10 M‐Lys‐UP” dataset only contains nine unique differentially expressed proteins. The third bar shows that “4 M‐Lys‐UP” and “10 M‐Lys‐UP” share 23 differentially expressed proteins, while the lack of an all‐datasets bar shows that there are zero differentially expressed genes that are shared across all four datasets. (E) Table summarizing key proteins that are up‐ or downregulated in early and advanced cohorts. ANK3, ankyrin‐G; ANXA6, Annexin A6; Anxa6, annexin A6; APP, amyloid precursor protein; Atp6v1g1, Vacuolar ATPase H^+^ transporting V1 subunit G1; Calb1, calbindin 1; GAPDH, glyceraldehyde 3‐phosphate dehydrogenase; HCN2, hyperpolarization‐activated cyclic nucleotide‐gated channel 2; HPRT1, Hypoxanthine Phosphoribosyltransferase 1; Ist1, IST1 factor associated with ESCRT‐III; LAMP2, lysosomal‐associated membrane protein 2; Lrp1, low‐density lipoprotein receptor‐related protein 1; Myo6, myosin VI; Nbea, neurobeactin; Ndufaf2, NADH: ubiquinone oxidoreductase complex assembly factor 2; PDCD6, programmed cell death 6; Pfn2, profilin 2; Psap, prosaponin; SEM, standard error of the mean; Sorl1, sortilin‐related receptor 1; Uchl1, ubiquitin C‐terminal hydrolase L1; Vamp3, vesicle associated membrane protein 3; Vdac1, voltage‐dependent anion channel 1; WT, wild type.

To assess the effect of Tau35 overexpression on the lysosomal proteome in the brain during disease progression, lysosome‐enriched fractions were isolated from the brains of WT and Tau35 mice at early (4 months) and advanced (10 months) stages of tau pathology. This was achieved through differential and subsequent discontinuous iodixanol gradient centrifugation. The process is outlined in a flowchart, accompanied by representative images of the iodixanol gradient, showing enriched subcellular fractions (lysosomal [LF] and mitochondrial [MF]) from both WT and Tau35 mice (Figure 2A and Figure S2A). Although recent LysoIP methods offer greater purity and specificity, particularly for cell‐type‐specific studies,^37^ we opted for density gradient centrifugation to obtain a higher lysosome yield. We assessed specificity by comparing detected proteins with curated lysosomal protein lists and performing Western blot analysis of fractions using specific lysosomal markers. Western blotting analysis of brain lysates and different fractions obtained showed highest enrichment of lysosomes in the LF characterized by the lysosomal membrane protein LAMP2 (Figure 2B) and highest enrichment of mitochondria in the MF characterized by the mitochondrial transmembrane protein MCU (Figure S2B), indicating sufficient separation between the organelles. The lysosomal enrichment and yield of fractions were comparable between WT and Tau35 preparations (Figure 2B and Figure S2B). Specifically, we observed 10‐fold and 10‐ to 20‐fold enrichment in the WT/Tau35 LFs in the early and advanced disease stages, respectively (Figure 2B). To further assess specificity, detected proteins were cross‐referenced with a curated list of autophagy and endo‐lysosomal proteins.^29^, ^38^, ^39^, ^40^ Lysosome‐related proteins accounted for 24% of total proteins (in both 4‐ and 10‐month groups), consistent with previous reports^41^, ^42^ using similar fraction isolation methods.

We initially conducted a rigorous (*p* value cutoff [*p*‐cutoff]: *p *→ 0.05, fold change cutoff [fc‐cutoff]: fc → 1) analysis of the proteomic dataset obtained through LC‐MS/MS using the limma package in R. This analysis identified five proteins that were differentially expressed in both the early and advanced disease stage cohorts (early cohort: 34 up, 15 down; advanced cohort: 12 up, nine down) (File S1), with three of these proteins (Vamp3, Atp6v1g1, and Pdcd6) showing upregulated lysosomal processing in Tau35 samples across both stages (Figure 2C).

We then relaxed the analysis parameters and conducted a discovery (*p*‐cutoff: *p *→ 0.05, fc‐cutoff: fc → 0.05) analysis to identify intracellular mechanisms and pathways influenced by Tau35 overexpression. This approach revealed 55 proteins that were differentially expressed in both the early and advanced disease stage cohorts (early cohort: 148 up, 136 down; advanced cohort: 50 up, 18 down) (Figure 2C, D, Figure S2C, and File S1).

Differentially expressed proteins identified using the limma package were cross‐referenced with a curated list of autophagy and endo‐lysosomal pathway‐associated proteins^29^ to pinpoint ALP components showing altered expression in Tau35 brains. Notably, several proteins involved in autophagy and endo‐lysosomal processes indicated altered lysosomal processing in Tau35 samples across both stages (early cohort: 31 altered, 13 up, 18 down; advanced cohort: seven altered, six up, one down). Many of them showed upregulated lysosomal processing in Tau35 samples across both stages (Figure S2D), alongside key modulators of tau (e.g., LRP1) and intracellular organelle acidification (e.g., components of the v‐ATPase) (Figure 2E). GO analysis of overrepresented terms in both cohorts revealed associations with mitochondria, energy/metabolism dynamics, and neuronal cellular homeostasis (Figure S2E,F), underscoring the interconnectedness of intracellular processes and the critical role of the endo‐lysosomal system in maintaining cellular homeostasis during disease progression.

### 3.3 Excessive endocytic activity disrupts autophagic flux and lysosomal proteolytic function in human neuroblastoma SH‐SY5Y tau models

To explore the impact of disease‐associated tau cleavage on proteolysis and endocytosis, we assessed the proteolytic capacity of differentiated SH‐SY5Y cells overexpressing N‐terminally truncated Tau35, compared to WT control cells and cells overexpressing full‐length tau (FL‐tau).

For this purpose, we generated and characterized six novel tauopathy cell lines, each stably expressing either Tau35 or FL‐tau fused to epitope tags or fluorescent reporters (construct maps and Western blots are provided in File S2A and B), along with a control SH‐SY5Y line stably expressing enhanced green fluorescent protein (eGFP) (File S2C). Western blotting was employed to analyze protein expression and confirm that the fusion proteins were full length and not degraded upon overexpression (File 2B). In this study, we focused on two of these newly generated SH‐SY5Y tauopathy lines: one expressing Tau35‐HA (referred to as Tau35) and one expressing Avi‐FL tau (referred to as FL‐tau) (File S2C).

We then differentiated the cells, as differentiated SH‐SY5Y cells exhibit a polarized morphology similar to neurons (protocol schematic, Figure 3A). Overexpression of Tau35 and FL‐tau led to a significant increase in p62 levels (Figure S3B), a marker of autophagic flux, while eGFP overexpression alone did not induce any changes in p62 levels (File S2D). This suggests that the observed p62 increase was specific to tau overexpression, making the Tau35‐ and FL‐tau‐overexpressing lines suitable models for studying alterations in autophagy and endo‐lysosomal processes.

FIGURE 3Endocytic dysregulation and defective proteolysis in SH‐SY5Y tau models. (A) Schematic illustrating the three differentiated SH‐SY5Y cell lines; control SH‐SY5Y cells, cells overexpressing (OE) Tau35‐HA (referred to as Tau35), and cells overexpressing (OE) Avi‐FL tau (referred to as FL‐tau). Diagrammatic representation of SH‐SY5Y differentiation protocol detailing timeline of media changes and various supplements administered. (B) Representative images of control, Tau35, and FL‐tau differentiated cells at DIV14 after 4 h incubation with both DQ‐Red BSA and Alexa‐488 BSA probes and counterstained with NucBlue reagent. Scale bar: 10 µm. (C) Representative schematic illustrating normal, defective, and altered proteolysis and endocytosis. Quantifications showing the mean DQ‐Red BSA/BSA‐488 ratio, the mean DQ‐Red BSA fluorescent intensity, and the mean BSA‐488 fluorescent intensity in control, Tau35, and FL‐tau differentiated cells. Quantification of blots is shown in graphs as mean ± SEM, *n* = 3 independent experiments. (D) Basal and treatment‐induced changes in p62 and LC3‐II/I levels in control, Tau35, and full‐length tau SH‐SY5Y lines. Schematic overview of three differentiated SH‐SY5Y cell lines and associated pharmacological treatment conditions used to assess changes in autophagy dynamics. Bafylomycin A1 and chloroquine were used to inhibit autophagy and block endo‐lysosomal acidification, while Torin 1 and AZD8055 were used to induce autophagy through mTOR inhibition. Western blots of total cell lysates from SH‐SY5Y, Tau35‐OE, and FL tau‐OE lines were probed with antibodies to p62, LC3, and GAPDH. Quantification of the blots is shown in the graphs as mean ± SEM, *n* = 5 independent experiments. Two‐way ANOVA, ^*^ *p* < 0.05, ^**^ *p* < 0.01, ^***^ *p* < 0.001; SEM, standard error of the mean. (E) Schematic representation of endo‐lysosome regulation and drug effects in the course of autophagy. (1) Induction of lysosome production driven by TFEB activation following inhibition of mTORC1 signaling. Autophagy initiation by the ULK1 complex, (2) phagophore formation, (3) membrane elongation, (4) autophagosome‐lysosome fusion/Lysosomal acidification, (5) endosomal acidification, (6) autolysosome formation, and (7) degradation. Drug effects are mapped along the autophagy pathway, highlighting key points of potential dysregulation in Tau35‐OE cells. mTORC1, mechanistic target of rapamycin complex 1; GAPDH, glyceraldehyde 3‐phosphate dehydrogenase; LC3, microtubule‐associated protein light chain 3; p62/SQSTM1, sequestosome‐1; SEM, standard error of the mean; TFEB, transcription factor EB; ULK1, UNC51‐like kinase 1.

**Figure:**
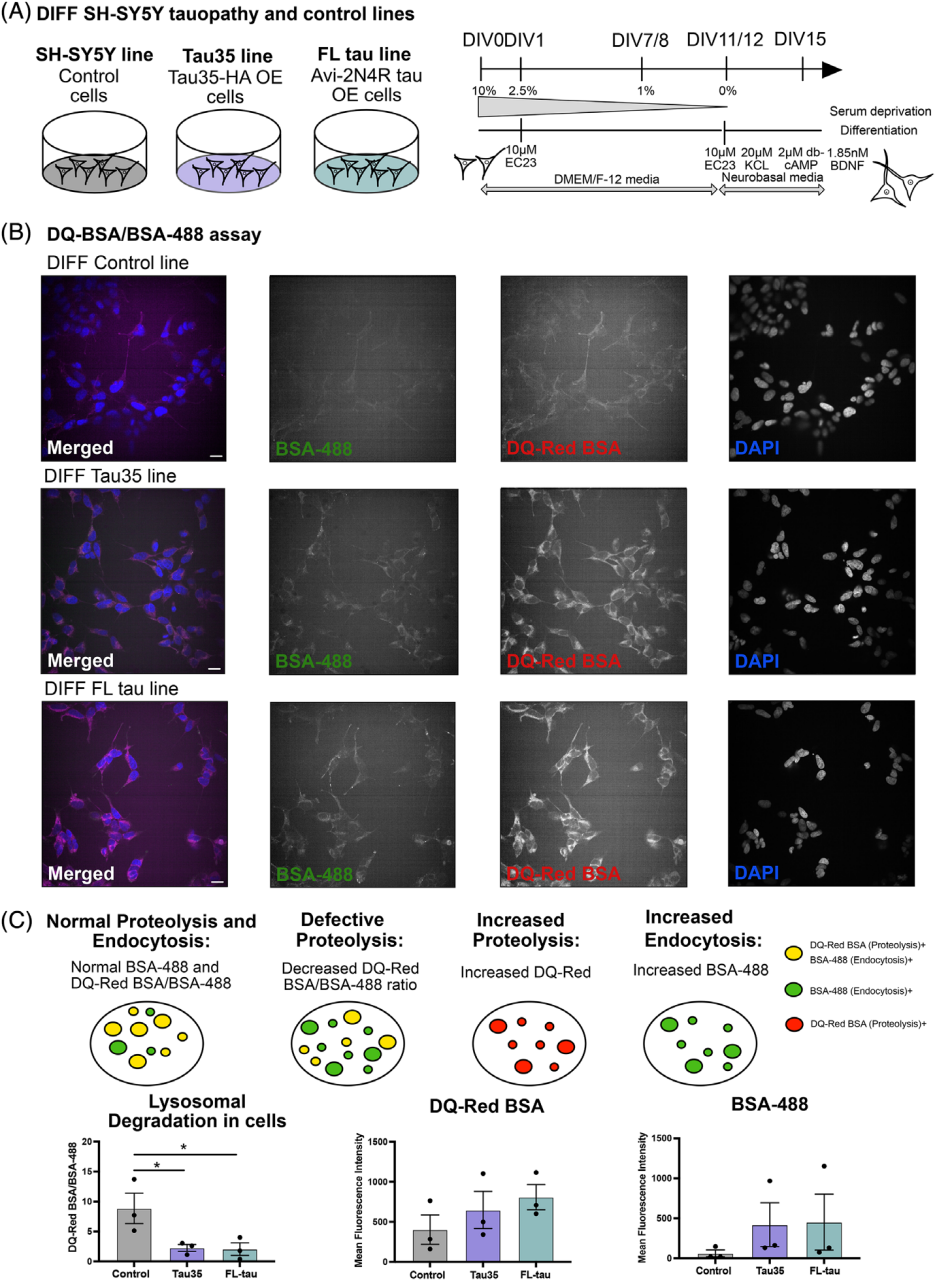

**Figure:**
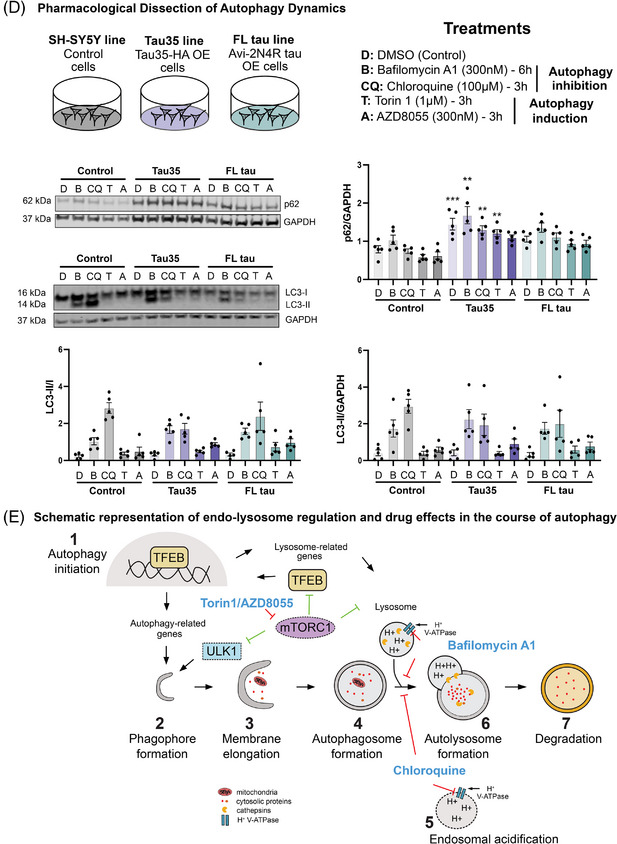

We treated the cells with both DQ‐Red BSA and Alexa‐488 BSA for 4 h to investigate endo‐lysosomal changes and counterstained with NucBlue reagent to facilitate the detection and segmentation of nuclei (representative images for each cell line are shown in Figure 3B). Measurements of lysosomal proteolytic activity by DQ‐Red BSA fluorescence normalized to BSA‐488 in differentiated SH‐SY5Y cells revealed that Tau35‐ and FL‐tau‐overexpressing cells had significantly reduced activity compared to controls (Figure 3B and C). Although tau‐overexpressing cells showed decreased lysosomal proteolytic activity, as indicated by lower DQ‐Red BSA fluorescence normalized to Alexa‐488‐BSA fluorescence, the substantial reduction was primarily driven by a marked increase in endocytic activity as indicated by Alexa‐488‐BSA fluorescence (Figure 3C). Tau‐overexpressing lines exhibited a sevenfold increase in Alexa‐488‐BSA fluorescence compared to controls, while DQ‐Red BSA fluorescence increased by 1.6‐fold (Tau35) and twofold (FL‐tau). These findings suggest that excessive endocytic activity can negatively impact lysosomal proteolytic function in differentiated SH‐SY5Y cells with neuronal‐like morphology. Neuronal endosomal dysfunction, a characteristic feature of early AD, involves increased activity within the endocytic pathway, driven by elevated expression of Rab GTPases^43^ or enhanced trafficking through early endosomes.^44^ This dysfunction is a critical factor in AD progression and is consistent with our findings of markedly elevated endocytic activity, highlighting its potential contribution to the underlying disease pathology.

To further investigate whether tau overexpression affected autophagy and lysosomal function at the molecular level, we performed Western blot analysis of SH‐SY5Y cell lysates. This revealed a significant increase in the LC3‐II/I ratio and p62 levels in both Tau35 and full‐length tau‐overexpressing SH‐SY5Y cells, indicating altered autophagy‐associated protein expression (Figure S3B). In line with findings from the mouse model, expression of mature cathepsin B (CTSB) was significantly reduced in tau‐overexpressing SH‐SY5Y cells. Interestingly, however, unlike in the mouse model, the immature CTSB form was markedly increased in both Tau35 and full‐length tau‐overexpressing cells. Consistent with mouse findings, Tau35‐overexpressing SH‐SY5Y cells also showed a significant increase in mature cathepsin L (CTSL). The accumulation of immature CTSB, together with elevated mature CTSL, may reflect a compensatory adjustment within SH‐SY5Y cells, potentially aimed at counteracting reductions in mature CTSB. No significant changes were observed in mature cathepsin D (CTSD) expression, although a trend toward increased levels of both the active and pro‐cathepsin forms was observed, particularly in the Tau35‐overexpressing line (Figure S3C). LAMP1 expression was significantly elevated in tau‐overexpressing cells, whereas LAMP2 levels remained unchanged (Figure S3D). These results, together with the impaired proteolytic activity observed in DQ‐BSA assays, suggest an accumulation of autophagic intermediates and potential disruption in lysosomal degradation or autophagosome–lysosome fusion.

We next sought to dissect specific endo‐lysosomal regulation and autophagy dynamics via pharmacological interventions. At baseline, Tau35 cells exhibited an 83% increase in p62 compared to control SH‐SY5Y cells, while LC3‐II/GAPDH and LC3‐II/I ratios were unchanged, suggesting a higher autophagic cargo load in Tau35 cells under resting conditions (Figure 3D). To further assess autophagic dynamics, cells were treated with pharmacological modulators: BafA1 and CQ to inhibit autophagy and disrupt endo‐lysosomal acidification, and Torin 1 and AZD8055 to induce autophagy via mTOR inhibition (Figure 3D). All cell lines (SH‐SY5Y control, Tau35, and FL‐tau) corresponded to treatment with the expected directional changes in p62: BafA1 and CQ increased p62 levels, while Torin 1 and AZD8055 decreased them. However, p62 levels in Tau35‐overexpressing cells remained consistently higher than in the respective control SH‐SY5Y groups, suggesting reduced efficiency in cargo clearance despite preserved response directionality (Figure 3D). LC3‐II/GAPDH and LC3‐II/I ratios followed a similar pattern in response to BafA1 and Torin 1 across lines, whereas CQ treatment resulted in more pronounced LC3‐II/GAPDH and LC3‐II/I accumulation in controls than in Tau35‐overexpressing cells, indicating a potential difference in autophagosome maturation, flux, or endo‐lysosomal acidification capacity in the Tau35 line (Figure 3D). These interpretations are illustrated in Figure 3E, a schematic representation of endo‐lysosome regulation and drug effects in the course of autophagy, highlighting key points in the pathway where Tau35‐dependent changes may occur.

### 3.4 Altered endo‐lysosomal trafficking dynamics in human neuroblastoma SH‐SY5Y tau models

To further investigate the impact of Tau35 overexpression on endo‐lysosomal motility and trafficking, we performed super‐resolution live‐cell imaging of lysosomes using LysoTracker on differentiated control, Tau35, or FL‐tau SH‐SY5Y cells. Given the excellent cell permeability, high specificity, and low cytotoxicity of LysoTracker Deep Red (647), we utilized this probe for live‐cell iSIM imaging. By applying the probe in iSIM imaging, we were able to record the dynamic processes of lysosome motility and interactions in live cells with a spatial resolution of ∼200 nm (representative images for each cell line are shown in Figure 4A, representative video of live‐cell iSIM imaging in File S3).

FIGURE 4Lysosomal motility dynamics in SH‐SY5Y tau models. (A) Super‐resolution microscopy images of LysoTracker staining in control, Tau35, and FL‐tau differentiated cells at DIV14. Cells were stained with LysoTracker Deep Red (magenta) and counterstained for nuclei with NucBlue reagent (blue). Scale bar: 10 µm. (B–E) Quantification of lysosome motility parameters obtained by analyzing their trajectories. Simple box plots (left) and raincloud plots (right) combining a split‐half violin, raw jittered data points, and a standard visualization of central tendency with boxplot of distance traveled (µm) (B), straightness of measured track (C), mean velocity (µm/s) (D), and mean square displacement (µm2) (E) parameters in all three cell lines. (E) A segmented raincloud plot for mean square displacement (µm^2^) is shown to better illustrate the distribution of values within specific ranges (0 to 200, 400 to 1000, 1200 to 1600) across the different cell lines. (F) Rose plots illustrating the angles of displacement for LysoTracker‐detected lysosome motility in all three cell lines are presented. The bootstrapping technique was used to create random subsets of data (1000 bootstrap replicates for each parameter), derived from the original dataset containing 24,000 to 43,000 data points (spots) per cell line, in order to minimize the impact of large datasets potentially obscuring differences. (B–F) Quantification of the blots is shown in the graphs as mean ± SEM, *n* = 10 to 12 independent experiments; Kruskal–Wallis's test; ^**^ *p* < 0.05, ^**^ *p* < 0.01; SEM, standard error of the mean.

**Figure:**
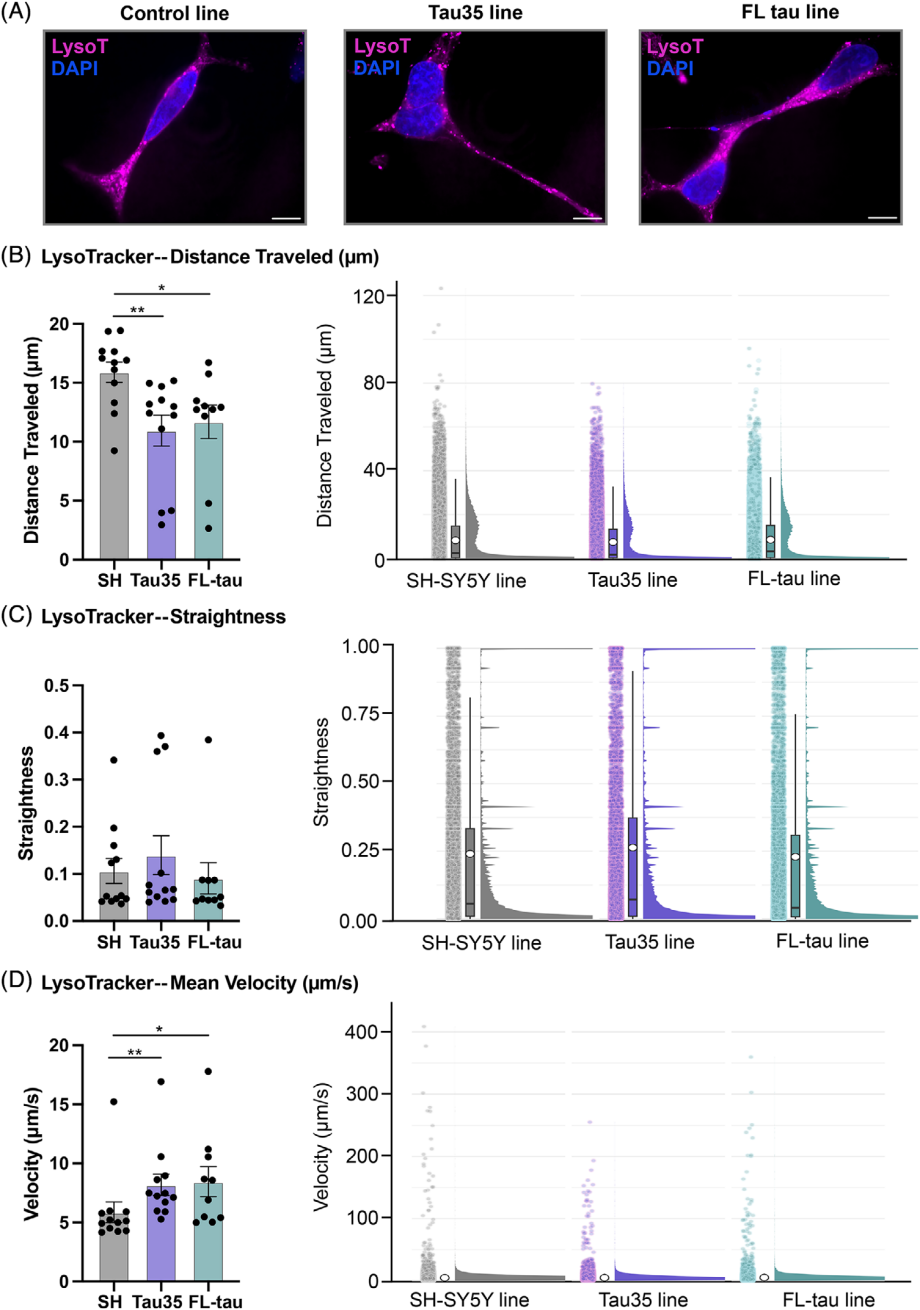

**Figure:**
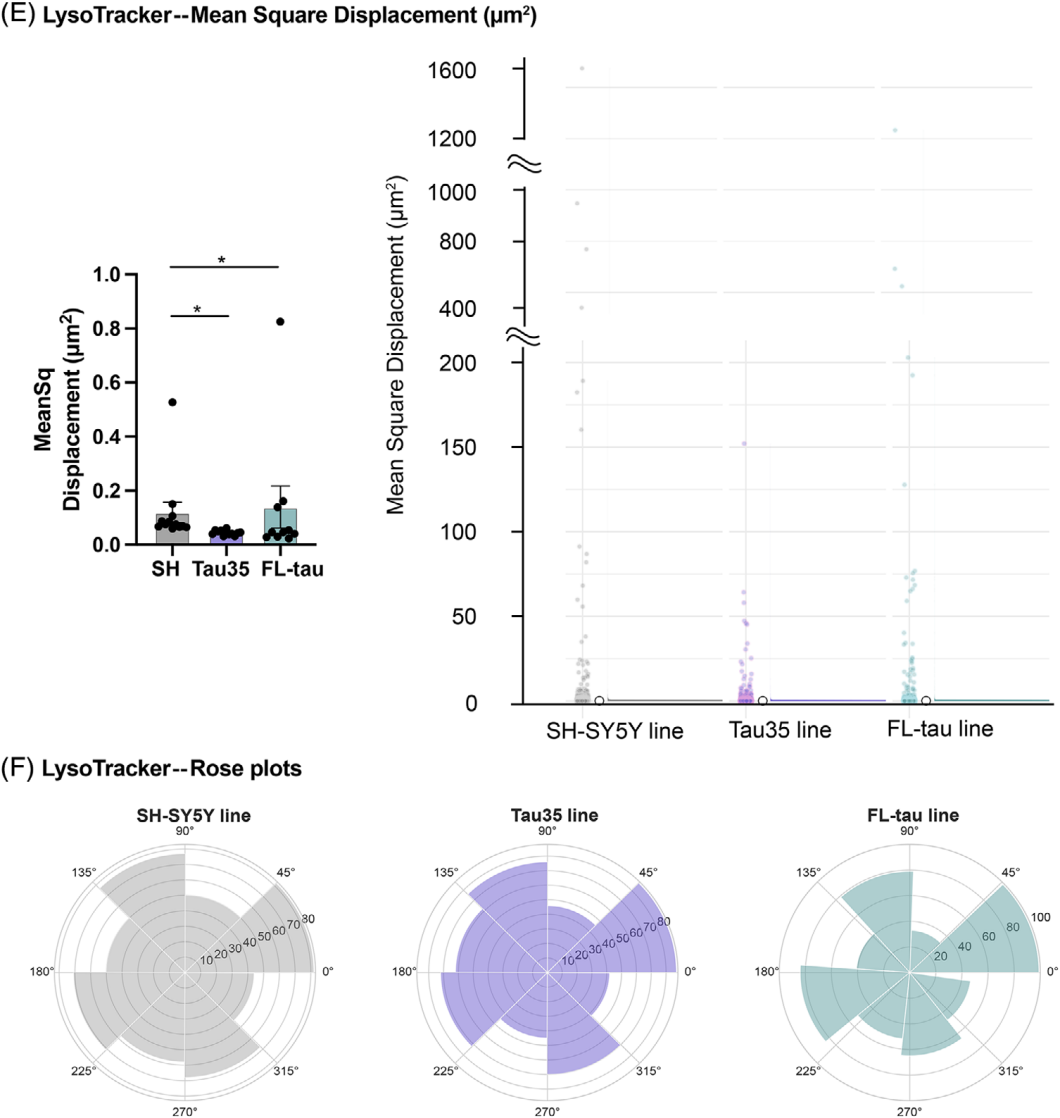

Lysosomes, dynamic organelles with both stationary and mobile pools, play key biological roles, and their dysfunction is linked to diseases such as cancer, autoimmune, and neurodegenerative disorders.^45^ Therefore, understanding changes in lysosome motility can provide insights into how disruptions in lysosomal dynamics contribute to disease progression. Using LysoTracker imaging, we initially examined basic lysosomal parameters. No differences were observed in total cell area, the number of LysoTracker spots per cell or per area, or the mean spot intensity across all cell lines (Figure S4A). We then investigated the trafficking of lysosomes by acquiring images of stained live organelles at 100 fps over 10 ms. Their motility was calculated by the assumption that immobile lysosomes move <0.2 µm in their track displacement. Lysosomes in the Tau35 cell line exhibited a shorter travel distance moving 31% less than those in the control line, while lysosomes in the FL‐tau line traveled 26% less compared to the control (Figure 4B). It is important to note, however, that the highest absolute distance values, exceeding 100 µm, were observed in the control line, as compared to the Tau35 and FL‐tau lines (Figure 4B). We then investigated whether the lysosomes in the tested cell lines exhibited directed motion or a more diffusive pattern. Lysosomes in the control and FL‐tau lines moved in a more organized manner, with the FL‐tau line showing more linear movement compared to the others. In contrast, lysosomes in the Tau35 line displayed a more diffusive motion than those in the other lines (Figure 4C). Additionally, the mean speed of lysosomes was higher in both tau‐overexpressing cell lines compared to control SH‐SY5Y cells. Specifically, lysosomes in the Tau35 and FL‐tau cell lines showed a 40% and 44% increase, respectively, in mean velocity. It is noteworthy, however, that lysosomes in the mobile pools of tau‐overexpressing cells, particularly in the Tau35 line, exhibit reduced maximum speed (250 µm/s) compared to control cells (exceeding 400 µm/s), indicating altered lysosomal motility dynamics (Figure 4D). This observation is further corroborated by the mean square displacement measurements, which show a 62% decrease in mean square displacement for lysosomes in the Tau35 line compared to the control line. Notably, the maximum mean square displacement value for lysosomes in the Tau35 line was only 150 µm^2^, which is substantially lower than the maximum values observed in the control (1593 µm2) and FL‐tau (>1291 µm^2^) lines, highlighting a significant reduction in lysosomal mobility in the Tau35 line (Figure 4E and Figure S4F). Figure 4E shows a segmented raincloud plot for mean square displacement (µm^2^), highlighting value distributions within specific ranges (0 to 200, 400 to 1000, 1200 to 1600) across cell lines, while Figure S4F presents the original raincloud plot, illustrating the full range of values in proportion. These findings may reflect impaired trafficking efficiency or dysregulated lysosomal transport mechanisms in tau‐overexpressing cells, potentially affecting their ability to maintain spatial distribution and engage in local degradation processes.

Next, to examine the directedness of the lysosomes, we constructed cellular histograms (rose plots). Rose plots depicting the displacement angles of LysoTracker‐detected lysosome motility across the three cell lines are presented, illustrating the movement of individual lysosomes (spots) from their initial positions at the start of video acquisition. Lysosomes in the control line appeared to travel farther from their starting positions compared to those in the tau‐overexpressing lines. Lysosomes in the FL‐tau line exhibited the least deviation, with most of their movement restricted to the range of angles between 0° to 45°, 90° to 135°, and 175° to 220° (Figure 4F). These findings may reflect the stochastic shared movement orientation among lysosomes, which reduces independent angle sampling and produces a multimodal distribution of movement angles. The bootstrapping technique, renowned for improving statistical analysis by facilitating robust comparisons across scenarios and distributions while supporting careful hypothesis evaluation,^46^ was employed to generate 1000 random subsets per parameter from datasets containing 24,000 to 43,000 data points per cell line. This approach minimized the risk of large datasets masking subtle differences and validated our findings on lysosome motility (Figure S4B–E).

### 3.5 Functional assessment of cathepsin activity and lysosomal acidification in Tau35 models

Having observed significant alterations in the expression of key cathepsins in both the mouse and SH‐SY5Y models, we next investigated whether these changes were reflected at the functional level by assessing cathepsin activity using commercial fluorometric assays for cathepsin B and cathepsin D. Cathepsins are a family of lysosomal proteases with critical roles in maintaining neuronal homeostasis and influencing processes such as neuroinflammation, synaptic remodeling, and neurodegeneration. Among them, cathepsins B, D, and L have been reported to exert the strongest impact on the progression of AD and related tauopathies.^47^

**FIGURE 5:**
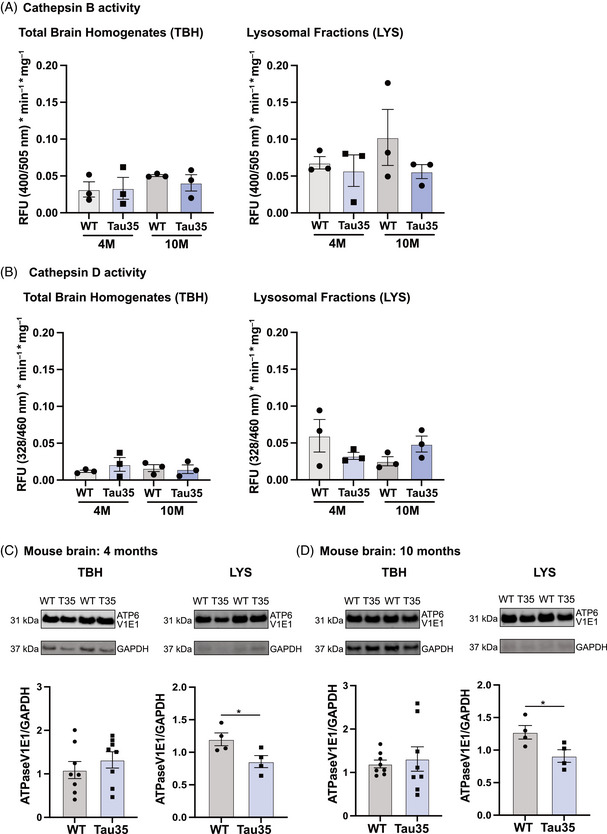
Assessment of cathepsin B and D activity and regulation of v‐ATPase in Tau35 mouse samples. Cathepsin B and D activity was measured in brain homogenates and lysosomal fractions (LFs) using commercial fluorometric assays. Activity is presented as blank corrected fluorescence, normalized to incubation time and total protein content. (A) Quantification of cathepsin B activity in total brain homogenates (TBHs) and lysosomal fractions (LYS) from early (4 months) and advanced (10 months) disease stages, showed a non‐significant trend toward reduction in Tau35 samples, most pronounced in LFs at 10 months. (B) Quantification of cathepsin D activity in TBH and LYS from both disease stages showed that cathepsin D activity remained largely unchanged. (C) Lysosomal acidification was assessed via ATPase expression. Western blots of TBH and LYS samples from WT and Tau35 mice aged 4 and 10 months, respectively, were probed with antibodies to ATP6V1E1 and glyceraldehyde 3‐phosphate dehydrogenase (GAPDH) and showed a significant reduction of ATP6V1E1 expression in Tau35 mouse lysosomal fractions. Quantification of the blots is shown in the graphs as mean ± SEM, *n* = 4 to 8 brains per group. Student's *t* test, *p < 0.05; SEM, standard error of the mean.

To test whether reduced levels of active CTSB in Tau35 mouse brains and Tau35‐overexpressing cells translated into altered enzymatic activity, we measured cathepsin B activity in brain homogenates and lysosomal fractions from early (4 months) and advanced (10 months) disease stages, as well as in lysates from differentiated SH‐SY5Y cells. In mouse brain samples, we observed a trend toward reduced cathepsin B activity in Tau35 compared to WT, most notably in lysosomal fractions at 10 months (45.3% reduction), although this did not reach statistical significance (Figure 5A). Cathepsin D activity, measured in parallel as a control, showed minor non‐significant fluctuations, consistent with unaltered protein levels (Figure 5B,C), In contrast, SH‐SY5Y cells displayed a significant increase in cathepsin B activity in Tau35 and FL‐tau overexpressing lines (Figure S5A), despite Western blotting indicating a reduced active‐to‐pro‐cathepsin ratio (Figure S3B). This likely reflects the overall increase in both mature and immature cathepsin B levels detected by immunoblotting. Similarly, cathepsin D activity showed a non‐significant upward trend in tau‐overexpressing cells, paralleling protein expression changes (Figures S3C and S5B). To investigate whether lysosomal acidification could contribute to these discrepancies, we assessed ATP6V1E1, a v‐ATPase subunit identified as differentially expressed in our proteomics data (Figure 2E). In Tau35 mouse lysosomal fractions, but not total brain homogenates, ATP6V1E1 levels were significantly reduced at both disease stages, while tau‐overexpressing SH‐SY5Y cells showed a modest, non‐significant increase (Figure 5C and Figure S5C), suggesting lysosome‐specific deregulation of ATP6V1E1 in mouse brain and possibly differential regulation of lysosomal pH homeostasis between models.

## 4 DISCUSSION

Endo‐lysosomal dysfunction is increasingly being recognized as a key contributor to neurodegenerative disease pathogenesis.^36^ In AD, early defects in autophagy and lysosomal clearance promote Aβ and tau accumulation, paralleling lysosomal pathologies.^6^, ^10^ Normally, tau stabilizes microtubules and regulates axonal transport, but mutations or post‐translational modifications trigger its detachment and aggregation.^48^ Impaired autophagosome‐lysosome fusion, disrupted acidification, or altered protease activity, impede tau degradation,^2^ while pathogenic tau reciprocally disrupts endo‐lysosomal trafficking and Rab GTPase regulation,^49^ creating a self‐reinforcing cycle that accelerates neurodegeneration.

Tau truncation contributes to disease via fragment‐specific mechanisms.^15^, ^16^ Overexpression of the C‐terminal Tau35 fragment drives tau phosphorylation, synaptic alterations, behavioral deficits, and impaired proteasome, lysosome, and autophagy function.^19^, ^20^, ^21^, ^22^, ^23^ In Tau35 mice, disruptions include altered kinase activity,^17^, ^18^ compromised synaptic processes,^20^ and defective lysosomal degradation. Late‐stage animals accumulate p62 and LC3I/II and show reduced active cathepsin D,^19^ underscoring Tau35's role in progressive neuronal dysfunction.

Tau accumulation thus reflects disrupted protein homeostasis and impaired endo‐lysosomal and autophagic clearance.^2^ Growing genetic and molecular evidence^6^, ^9^, ^10^, ^12^, ^50^ indicates that these dysfunctions extend beyond aggregation‐prone protein degradation, impacting broader aspects of tauopathy pathophysiology, such as synaptic function, intracellular trafficking, and neuronal survival.^2^, ^3^, ^49^ Key autophagic‐endolysosomal network (AELN) proteins, including LAMP1/2, Rab GTPases, legumain, GBA/GLB1, and cathepsins,^9^, ^10^, ^11^, ^12^, ^35^, ^50^, ^51^ are consistently altered in disease models and patient samples. Cathepsins, as the most abundant lysosomal proteases, directly degrade tau, and their loss or dysregulation promotes both pathogenic tau buildup^52^ and overall cellular destabilization. These observations underscore the central role of lysosomal proteostasis in maintaining neuronal health and highlight cathepsins^53^ and other AELN components as critical therapeutic targets for intervention in tauopathies.^54^

Our findings show that early‐stage (4 months) Tau35 mouse brains exhibit reduced mature CTSB, a pattern that persists in advanced stages (10 months), alongside an increase in mature CTSL expression. This shift in cathepsin balance may impair tau clearance, exacerbate tau pathology, and contribute to disease progression.^35^ Unlike earlier studies in the Tau35 models,^19^, ^23^ we did not observe changes in mature CTSD expression at the examined timepoints, potentially underscoring the delicate balance among cathepsins during disease progression. In advanced stages (10 months), the CTSB/CTSL imbalance coincides with reduced LAMP2, aligning with prior observations in the Tau35‐overexpressing CHO cell model.^23^ The decline in LAMP2 likely reflects compromised lysosomal function, as previously described in mice.^55^

AELN regulate tau clearance, including its degradation, release, and uptake by neurons and glial cells, ensuring proper tau homeostasis.^54^ Evidence suggests that dysfunction in these pathways may coincide with or precede tau pathology.^49^ To assess Tau35 overexpression effects, lysosome‐enriched brain fractions from WT and Tau35 mice at early (4 months) and advanced (10 months) tau pathology stages were analyzed using discontinuous iodixanol gradient centrifugation and proteomic analyses.

Our stringent proteomic analysis of Tau35 lysosome‐enriched fractions identified several differentially expressed proteins, which can be grouped into four key categories with roles in AELN processes and tau processing.

1. *pH maintenance and ionic homeostasis*: Proteins ATP6V1E1, ATP6V1G1, and SLC9A6 were dysregulated. ATP6V1E1 and ATP6V1G1, subunits of v‐ATPase, are essential for lysosomal acidification and autophagic flux maintenance, with dysfunction linked to impaired autophagy and tau aggregation. Proteomic studies showed that v‐ATPase H^+^ Transporting V1 Subunit E1 (ATP6V1E1), which regulates organelle acidification, is altered during tau pathology progression.^56^, ^57^ SLC9A6, which controls endosomal pH and volume, also impacts tau aggregation. SLC9A6 loss‐of‐function mutations disrupt vesicular targeting and are linked to tau inclusions, implicating it in tauopathies.^58^
2. *Tau regulation*: Proteins LRP1, SORL1, FKBP1A, and UCHL1 were altered. SORL1, a receptor involved in protein trafficking, plays a role in the trafficking and seeding of pathogenic tau.^59^ LRP1, often downregulated in tauopathies, regulates tau clearance and endo‐lysosomal trafficking.^60^ FKBP1A modulates tau phosphorylation and calcium homeostasis,^61^ while UCHL1, vital for proteostasis, contributes to tau aggregation and oxidative stress when downregulated.^62^
3. *Intracellular trafficking*: Proteins Myo6, LRP1, and VAMP3 were implicated. Myo6, which plays a role in vesicle transport and lysosomal degradation,^63^, ^64^ has been shown to co‐localize with fibrillary tau protein.^65^ VAMP3 dysregulation impairs vesicle transport, autophagy, and consequently tau clearance. In late‐onset AD, PICALM dysregulation disrupts the interaction and endocytosis of SNARE proteins, including VAMP3, further affecting tau removal.^66^ Additionally, LRP1 dysfunction is recognized as a contributor to increased tau accumulation.^60^
4. *Cytoskeletal dynamics*: ANK3 and PFN2, critical regulators of neuronal structure, were also impacted. ANK3, essential for microtubule stability and neuronal integrity, interacts with tau in Drosophila, causing lifespan and memory deficits.^67^ Similarly, PFN2, which regulates actin dynamics and intracellular trafficking, also interacts with and modulates tau.^68^

To explore Tau35‐associated pathways, we relaxed parameters for a discovery analysis, comparing differentially expressed proteins against a curated list of autophagy and endo‐lysosomal pathway‐associated proteins.^29^ This confirmed disruptions in ALP components at both early and advanced stages of disease. Consistent with our stringent analysis, key categories such as endo‐lysosomal ionic homeostasis, endo‐lysosomal trafficking, and cellular homeostasis were highlighted. The discovery analysis identified additional pathways, such as PI3K‐related processes, heat shock protein regulation, and Rab‐dependent autophagosome formation. Notably, recent research demonstrated that Rab5 overactivation, independent of APP‐βCTF, can mimic key Alzheimer's features, including synaptic deficits and tau hyperphosphorylation.^69^ GO analysis further highlighted mitochondria, energy/metabolism, and neuronal homeostasis terms, underscoring the interconnected nature of these processes and the essential role of the endo‐lysosomal system in maintaining cellular balance during disease progression.

We next assessed proteolysis and endocytosis in differentiated SH‐SY5Y cells overexpressing Tau35 or full‐length tau. Tau overexpression increased p62 and LC3‐II/I ratio levels, reduced CTSB, elevated CTSL and LAMP1, and impaired DQ‐BSA proteolysis, indicating accumulation of autophagic intermediates and endo‐lysosomal dysfunction. Pharmacological modulation with BafA1, CQ, Torin 1, and AZD8055 showed that upstream autophagy signaling remained largely preserved. However, Tau35 cells exhibited persistently elevated p62 and attenuated CQ‐induced LC3‐II/I accumulation, suggesting defects in autophagosome maturation, flux, or lysosomal acidification. Together, these findings indicate that Tau35 overexpression specifically burdens downstream degradation steps, compromising the ability of cells to efficiently process autophagic cargo. A schematic overview of the proposed disruptions is shown in Figure 3E.

Successful autophagic degradation of protein aggregates depends on coordinated endosomal sorting and maturation.^70^ Additionally, autophagosome biogenesis shares regulatory mechanisms with endosomal compartments and may rely on recycling endosomes for membrane sources, with key regulators of endosomal recycling acting at the intersection of these pathways.^70^, ^71^, ^72^ Together, these findings highlight the interconnected nature of autophagic and endo‐lysosomal trafficking, suggesting they cannot be considered separate, independent processes.^73^ In support of this, we observed reduced proteolytic capacity in tau‐overexpressing cells, driven by disproportionate endocytosis (sevenfold increase in BSA‐488 signal vs 1.6‐fold increase in DQ‐BSA signal). Such excessive endocytosis, also reported in early AD,^43^, ^44^ could impair lysosomal function, disrupt cellular homeostasis, and promote pathology.^49^, ^66^

Lysosomes exist in stationary perinuclear and mobile peripheral pools. Proper lysosomal movement underpins autophagy, protein degradation, and organelle turnover,^45^ and its disruption drives toxic protein accumulation, results in organelle stress, and contributes to neurodegeneration, cancer, and autoimmune conditions.^74^ Therefore, understanding motility changes may clarify disease progression and reveal therapeutic strategies to restore lysosomal function and maintain cellular balance.^75^

To assess truncated tau effects on endo‐lysosomal dynamics, we used live‐cell LysoTracker imaging in SH‐SY5Y cells expressing Tau35, FL‐tau, or controls. Basic lysosomal parameters were unchanged, but Tau35 cells showed markedly slower lysosomal movement, with reduced travel distance and displacement. By contrast, control and FL‐tau cells displayed more directed motility. These findings suggest that truncated tau disrupts lysosomal motility, potentially impairing function and contributing to tauopathy‐related cellular dysfunction.

To explore whether these disruptions in lysosomal dynamics were accompanied by functional consequences, we next assessed cathepsin B and D activity in both mouse and SH‐SY5Y models using commercial fluorometric assays. Overall, cathepsin B activity showed a trend toward a reduction in Tau35 mouse brains but was significantly increased in tau‐overexpressing SH‐SY5Y cells, while cathepsin D activity remained largely unchanged in both systems. Reduced brain activity may reflect impaired lysosomal acidification via ATP6V1E1 loss, whereas SH‐SY5Y cells showed compensatory cathepsin upregulation despite altered maturation. These differences may underscore model‐specific adaptations in lysosomal homeostasis, with SH‐SY5Y cells relying more strongly on compensatory mechanisms to preserve cathepsin function. The observed discrepancy between mouse and cell models may also reflect additional factors, including lower levels of endogenous cathepsin inhibitors, such as cystatins, in SH‐SY5Y cells and the contribution of glial cells in the brain environment.

Overall, our study highlights that Tau35 overexpression disrupts multiple aspects of endo‐lysosomal function, including lysosomal acidification, protease activity, autophagic flux, and organelle motility. Altered cathepsin activity, together with ATP6V1E1 dysregulation, indicates impaired lysosomal function as an early driver of neuronal pathology. The observed alterations in AELN components in Tau35 models align with previous findings in neurodegenerative diseases,^49^, ^53^ mirror aspects of lysosomal storage disorders,^13^ and further support endo‐lysosomal dysfunction as a convergent pathogenic mechanism in neurodegeneration. While model‐specific differences and the complexity of lysosomal regulation warrant caution in extrapolation, our results provide a framework for future studies and highlight endo‐lysosomal processes as promising therapeutic targets in tauopathies.

## AUTHOR CONTRIBUTIONS

Despoina Goniotaki, Deepak P. Srivastava, and Diane P. Hanger led the project. Despoina Goniotaki, Deepak P. Srivastava, Diane P. Hanger, and Graham Fraser designed and supervised the research. Despoina Goniotaki, Maximilian Hausherr, Steven Lynham, Ayushin Ale, and George Chennell performed the experiments and contributed to data acquisition. Despoina Goniotaki, Maximilian Hausherr, Steven Lynham, Ayushin Ale, Stefania Marcotti, and Deepak P. Srivastava contributed to data analysis and interpretation. Despoina Goniotaki wrote the paper. Maximilian Hausherr, Steven Lynham, George Chennell, Stefania Marcotti, Katrin Marcus, Wendy Noble, George Chennell, and Deepak P. Srivastava reviewed the paper. All authors read the manuscript.

## CONFLICT OF INTEREST STATEMENT

The authors declare no competing interests. Graham Fraser is an employee of AstraZeneca plc. Author disclosures are available in the supporting information.

## CONSENT STATEMENT

No human samples were used in this study, and therefore, informed consent and ethical approval were not necessary. All experimental procedures involving animal samples complied with the 1986 Animals (Scientific Procedures) Act and were approved by the local ethical review committee.

## Supporting information




Supporting Information

Supporting Information

Supporting Information

Supporting Information

Supporting Information

Supporting Information

Supporting Information

Supporting Information

Supporting Information

Supporting Information

## Data Availability

The raw mass spectrometry proteomics data have been deposited at the ProteomeXchange Consortium with the dataset identifier PXD062128 https://doi.org/10.6019/PXD062128. A full list of differentially expressed proteins of lysosome‐enriched mouse brain fractions identified with the R package limma can be found in File S1. All other datasets used and/or analyzed during the current study and material are available from the corresponding authors on reasonable request.

## References

[1] Gotz J , Halliday G , Nisbet RM . Molecular pathogenesis of the tauopathies. Annu Rev Pathol. 2019;14:239–261. doi:10.1146/annurev‐pathmechdis‐012418‐012936 30355155 10.1146/annurev-pathmechdis-012418-012936

[2] Lee MJ , Lee JH , Rubinsztein DC , Tau degradation: the ubiquitin‐proteasome system versus the autophagy‐lysosome system. Prog Neurobiol. 2013;105:49–59. doi:10.1016/j.pneurobio.2013.03.001 23528736 10.1016/j.pneurobio.2013.03.001

[3] Malik BR , Maddison DC , Smith GA , Peters OM . Autophagic and endo‐lysosomal dysfunction in neurodegenerative disease. Mol Brain. 2019;12(1):100. doi:10.1186/s13041‐019‐0504‐x 31783880 10.1186/s13041-019-0504-x PMC6884906

[4] Alcalay RN , Dinur T , Quinn T , et al. Comparison of Parkinson risk in Ashkenazi Jewish patients with Gaucher disease and GBA heterozygotes. JAMA Neurol. 2014;71(6):752–757. doi:10.1001/jamaneurol.2014.313 24756352 10.1001/jamaneurol.2014.313 PMC4082726

[5] Smith KR , Damiano J , Franceschetti S , et al. Strikingly different clinicopathological phenotypes determined by progranulin‐mutation dosage. Am J Hum Genet. 2012;90(6):1102–1107. doi:10.1016/j.ajhg.2012.04.021 22608501 10.1016/j.ajhg.2012.04.021 PMC3370276

[6] van Weering JRT , Scheper W . Endolysosome and autolysosome dysfunction in Alzheimer's disease: where intracellular and extracellular meet. CNS Drugs. 2019;33(7):639–648. doi:10.1007/s40263‐019‐00643‐1 31165364 10.1007/s40263-019-00643-1 PMC6647502

[7] Papassotiropoulos A , Bagli M , Kurz A , et al. A genetic variation of cathepsin D is a major risk factor for Alzheimer's disease. Ann Neurol. 2000;47(3):399–403. 10716266

[8] Sierksma A , Lu A , Mancuso R , et al. Novel Alzheimer risk genes determine the microglia response to amyloid‐beta but not to tau pathology. EMBO Mol Med. 2020;12(3):e10606. doi:10.15252/emmm.201910606 31951107 10.15252/emmm.201910606 PMC7059012

[9] Bellenguez C , Kucukali F , Jansen IE , et al. New insights into the genetic etiology of Alzheimer's disease and related dementias. Nat Genet. 2022;54(4):412–436. doi:10.1038/s41588‐022‐01024‐z 35379992 10.1038/s41588-022-01024-z PMC9005347

[10] Yan M , Zheng T . Role of the endolysosomal pathway and exosome release in tau propagation. Neurochem Int. 2021;145:104988. doi:10.1016/j.neuint.2021.104988 33582164 10.1016/j.neuint.2021.104988

[11] Sampognaro PJ , Arya S , Knudsen GM , et al. Mutations in alpha‐synuclein, TDP‐43 and tau prolong protein half‐life through diminished degradation by lysosomal proteases. Mol Neurodegener. 2023;18(1):29. doi:10.1186/s13024‐023‐00621‐8 37131250 10.1186/s13024-023-00621-8 PMC10155372

[12] Suire CN , Leissring MA . Cathepsin D: a candidate link between amyloid beta‐protein and tauopathy in Alzheimer disease. J Exp Neurol. 2021;2(1):10–15. 33665647 PMC7929084

[13] Ludlaim AM , Waddington SN , McKay TR . Unifying biology of neurodegeneration in lysosomal storage diseases. J Inherit Metab Dis. 2025;48(1):e12833. doi:10.1002/jimd.12833 39822020 10.1002/jimd.12833 PMC11739831

[14] Kametani F , Yoshida M , Matsubara T , et al. Comparison of common and disease‐specific post‐translational modifications of pathological tau associated with a wide range of tauopathies. Front Neurosci. 2020;14:581936. doi:10.3389/fnins.2020.581936 33250706 10.3389/fnins.2020.581936 PMC7672045

[15] Quinn JP , Corbett NJ , Kellett KAB , Hooper NM . Tau proteolysis in the pathogenesis of tauopathies: neurotoxic fragments and novel biomarkers. J Alzheimers Dis. 2018;63(1):13–33. doi:10.3233/JAD‐170959 29630551 10.3233/JAD-170959 PMC5900574

[16] Gu J , Xu W , Jin N , et al. Truncation of tau selectively facilitates its pathological activities. J Biol Chem. 2020;295(40):13812–13828. doi:10.1074/jbc.RA120.012587 32737201 10.1074/jbc.RA120.012587 PMC7535906

[17] Arai T , Ikeda K , Akiyama H , et al. Identification of amino‐terminally cleaved tau fragments that distinguish progressive supranuclear palsy from corticobasal degeneration. Ann Neurol. 2004;55(1):72–79. doi:10.1002/ana.10793 14705114 10.1002/ana.10793

[18] Wray S , Saxton M , Anderton BH , Hanger DP . Direct analysis of tau from PSP brain identifies new phosphorylation sites and a major fragment of N‐terminally cleaved tau containing four microtubule‐binding repeats. J Neurochem. 2008;105(6):2343–2352. doi:10.1111/j.1471‐4159.2008.05321.x 18315566 10.1111/j.1471-4159.2008.05321.x

[19] Bondulich MK , Guo T , Meehan C , et al. Tauopathy induced by low level expression of a human brain‐derived tau fragment in mice is rescued by phenylbutyrate. Brain. 2016;139(Pt 8):2290–2306. doi:10.1093/brain/aww137 27297240 10.1093/brain/aww137 PMC4958900

[20] Goniotaki D , Tamagnini F , Biasetti L , et al. Tau‐mediated synaptic dysfunction is coupled with HCN channelopathy. Alzheimers Dement. 2024;20(8):5629–5646. doi:10.1002/alz.14074 38994745 10.1002/alz.14074 PMC11350046

[21] Guo T , Dakkak D , Rodriguez‐Martin T , Noble W , Hanger DP . A pathogenic tau fragment compromises microtubules, disrupts insulin signaling and induces the unfolded protein response. Acta Neuropathol Commun. 2019;7(1):2. doi:10.1186/s40478‐018‐0651‐9 10.1186/s40478-018-0651-9 PMC6318896 30606258

[22] Tamagnini F , Walsh DA , Brown JT , Bondulich MK , Hanger DP , Randall AD . Hippocampal neurophysiology is modified by a disease‐associated C‐terminal fragment of tau protein. Neurobiol Aging. 2017;60:44–56. doi:10.1016/j.neurobiolaging.2017.07.005 28917666 10.1016/j.neurobiolaging.2017.07.005 PMC5654728

[23] Pollack SJ , Dakkak D , Guo T , et al. Truncated tau interferes with the autophagy and endolysosomal pathway and results in lipid accumulation. Cell Mol Life Sci. 2024;81(1):304. doi:10.1007/s00018‐024‐05337‐6 39009859 10.1007/s00018-024-05337-6 PMC11335226

[24] Percie du Sert N , Hurst V , Ahluwalia A , et al. The ARRIVE guidelines 2.0: updated guidelines for reporting animal research. BMJ Open Sci. 2020;4(1):e100115. doi:10.1136/bmjos‐2020‐100115 10.1136/bmjos-2020-100115 PMC7610906 34095516

[25] Eng JK , McCormack AL , Yates JR . An approach to correlate tandem mass spectral data of peptides with amino acid sequences in a protein database. J Am Soc Mass Spectrom. 1994;5(11):976–989. doi:10.1016/1044‐0305(94)80016‐2 24226387 10.1016/1044-0305(94)80016-2

[26] Ritchie ME , Phipson B , Wu D , et al. limma powers differential expression analyses for RNA‐sequencing and microarray studies. Nucleic Acids Res. 2015;43(7):e47. doi:10.1093/nar/gkv007 25605792 10.1093/nar/gkv007 PMC4402510

[27] van Ooijen MP , Jong VL , Eijkemans MJC , et al. Identification of differentially expressed peptides in high‐throughput proteomics data. Brief Bioinform. 2018;19(5):971–981. doi:10.1093/bib/bbx031 28369175 10.1093/bib/bbx031

[28] Yu G . Thirteen years of clusterProfiler. Innovation (Camb). 2024;5(6):100722. doi:10.1016/j.xinn.2024.100722 39529960 10.1016/j.xinn.2024.100722 PMC11551487

[29] Elsasser S , Elia, LP , Morimoto, RI , et al. A comprehensive enumeration of the human proteostasis network. 2. Components of the autophagy‐lysosome pathway. bioRxiv. 2023. doi:10.1101/2023.03.22.533675

[30] Perez‐Riverol Y , Bandla C , Kundu DJ , et al. The PRIDE database at 20 years: 2025 update. Nucleic Acids Res. 2025;53(D1):D543–D553. doi:10.1093/nar/gkae1011 39494541 10.1093/nar/gkae1011 PMC11701690

[31] Lukow DA , Sausville EL , Suri P , et al. Chromosomal instability accelerates the evolution of resistance to anti‐cancer therapies. Dev Cell. 2021;56(17):2427–2439 e4. doi:10.1016/j.devcel.2021.07.009 34352222 10.1016/j.devcel.2021.07.009 PMC8933054

[32] Shipley MM , Mangold CA , Szpara ML . Differentiation of the SH‐SY5Y human neuroblastoma cell line. J Vis Exp. 2016;108:53193. doi:10.3791/53193 10.3791/53193 PMC4828168 26967710

[33] Gatford NJF , Deans PJM , Duarte RRR , et al. Neuroligin‐3 and neuroligin‐4X form nanoscopic clusters and regulate growth cone organization and size. Hum Mol Genet. 2022;31(5):674–691. doi:10.1093/hmg/ddab277 34542148 10.1093/hmg/ddab277 PMC8895740

[34] Schneider CA , Rasband WS , Eliceiri KW . NIH Image to ImageJ: 25 years of image analysis. Nat Methods. 2012;9(7):671–675. doi:10.1038/nmeth.2089 22930834 10.1038/nmeth.2089 PMC5554542

[35] Drobny A , Prieto Huarcaya S , Dobert J , et al. The role of lysosomal cathepsins in neurodegeneration: mechanistic insights, diagnostic potential and therapeutic approaches. Biochim Biophys Acta Mol Cell Res. 2022;1869(7):119243. doi:10.1016/j.bbamcr.2022.119243 35217144 10.1016/j.bbamcr.2022.119243

[36] Herman M , Randall GW , Spiegel JL , Maldonado DJ , Simoes S . Endo‐lysosomal dysfunction in neurodegenerative diseases: opinion on current progress and future direction in the use of exosomes as biomarkers. Philos Trans R Soc Lond B Biol Sci. 2024;379(1899):20220387. doi:10.1098/rstb.2022.0387 38368936 10.1098/rstb.2022.0387 PMC10874701

[37] Singh J , Kaade E , Muntel J , et al. Systematic comparison of strategies for the enrichment of lysosomes by data independent acquisition. J Proteome Res. 2020;19(1):371–381. doi:10.1021/acs.jproteome.9b00580 31738065 10.1021/acs.jproteome.9b00580

[38] Winter D . Table 9_lysosomal protein list. figshare. Dataset. 2020;15:46. doi:10.6084/m9.figshare.11347025.v1

[39] Rouillard AD , Gundersen GW , Fernandez NF , et al. The harmonizome: a collection of processed datasets gathered to serve and mine knowledge about genes and proteins. Database (Oxford). 2016;2016:baw100. doi:10.1093/database/baw100 27374120 10.1093/database/baw100 PMC4930834

[40] Diamant I , Clarke DJB , Evangelista JE , Lingam N , Ma'ayan A . Harmonizome 3.0: integrated knowledge about genes and proteins from diverse multi‐omics resources. Nucleic Acids Res. 2025;53(D1):D1016–D1028. doi:10.1093/nar/gkae1080 39565209 10.1093/nar/gkae1080 PMC11701526

[41] Graham JM . Isolation of lysosomes from tissues and cells by differential and density gradient centrifugation. Curr Protoc Cell Biol. 2001;Chapter 3:Unit 3 6. doi:10.1002/0471143030.cb0306s07 10.1002/0471143030.cb0306s07 18228358

[42] Ponnaiyan S , Akter F , Singh J , Winter D . Comprehensive draft of the mouse embryonic fibroblast lysosomal proteome by mass spectrometry based proteomics. Sci Data. 2020;7(1):68. doi:10.1038/s41597‐020‐0399‐5 32103020 10.1038/s41597-020-0399-5 PMC7044164

[43] Ginsberg SD , Alldred MJ , Counts SE , et al. Microarray analysis of hippocampal CA1 neurons implicates early endosomal dysfunction during Alzheimer's disease progression. Biol Psychiatry. 2010;68(10):885–893. doi:10.1016/j.biopsych.2010.05.030 20655510 10.1016/j.biopsych.2010.05.030 PMC2965820

[44] Cataldo AM , Mathews PM , Boiteau AB , et al. Down syndrome fibroblast model of Alzheimer‐related endosome pathology: accelerated endocytosis promotes late endocytic defects. Am J Pathol. 2008;173(2):370–384. doi:10.2353/ajpath.2008.071053 18535180 10.2353/ajpath.2008.071053 PMC2475775

[45] Cabukusta B , Neefjes J . Mechanisms of lysosomal positioning and movement. Traffic. 2018;19(10):761–769. doi:10.1111/tra.12587 29900632 10.1111/tra.12587 PMC6175085

[46] Kostanek J , Karolczak, K. , Kuliczkowski, W. , Watala, C . Bootstrap method as a tool for analyzing data with atypical distributions deviating from parametric assumptions: critique and effectiveness evaluation. Data. 2024;9(8):95. doi:10.3390/data9080095

[47] Lewandowski D , Konieczny M , Rozycka A , et al. Cathepsins in neurological diseases. Int J Mol Sci. 2025;26(16):7886. doi:10.3390/ijms26167886 40869205 10.3390/ijms26167886 PMC12386458

[48] Guo T , Noble W , Hanger DP . Roles of tau protein in health and disease. Acta Neuropathol. 2017;133(5):665–704. doi:10.1007/s00401‐017‐1707‐9 28386764 10.1007/s00401-017-1707-9 PMC5390006

[49] Peric A , Annaert W . Early etiology of Alzheimer's disease: tipping the balance toward autophagy or endosomal dysfunction? Acta Neuropathol. 2015;129(3):363–381. doi:10.1007/s00401‐014‐1379‐7 25556159 10.1007/s00401-014-1379-7 PMC4331606

[50] Krance SH , Wu CY , Chan ACY , et al. Endosomal‐lysosomal and autophagy pathway in Alzheimer's disease: a systematic review and meta‐analysis. J Alzheimers Dis. 2022;88(4):1279–1292. doi:10.3233/JAD‐220360 35754279 10.3233/JAD-220360

[51] Zhang X , Zou L , Tang L , et al. Bridging integrator 1 fragment accelerates tau aggregation and propagation by enhancing clathrin‐mediated endocytosis in mice. PLoS Biol. 2024;22(1):e3002470. doi:10.1371/journal.pbio.3002470 38206965 10.1371/journal.pbio.3002470 PMC10783739

[52] Mancano ASF , Pina JG , Froes BR , Sciani JM . Autophagy‐lysosomal pathway impairment and cathepsin dysregulation in Alzheimer's disease. Front Mol Biosci. 2024;11:1490275. doi:10.3389/fmolb.2024.1490275 39544403 10.3389/fmolb.2024.1490275 PMC11560772

[53] Prieto Huarcaya S , Zunke F . Therapeutic potential of lysosomal cathepsins for neurodegenerative diseases. Neural Regen Res. 2023;18(8):1713–1714. doi:10.4103/1673‐5374.363181 36751788 10.4103/1673-5374.363181 PMC10154497

[54] Jiang S , Bhaskar K . Degradation and transmission of tau by autophagic‐endolysosomal networks and potential therapeutic targets for tauopathy. Front Mol Neurosci. 2020;13:586731. doi:10.3389/fnmol.2020.586731 33177989 10.3389/fnmol.2020.586731 PMC7596180

[55] Caballero B , Bourdenx M , Luengo E , et al. Acetylated tau inhibits chaperone‐mediated autophagy and promotes tau pathology propagation in mice. Nat Commun. 2021;12(1):2238. doi:10.1038/s41467‐021‐22501‐9 33854069 10.1038/s41467-021-22501-9 PMC8047017

[56] Chang SH , Jung IS , Han GY , Kim NH , Kim HJ , Kim CW . Proteomic profiling of brain cortex tissues in a Tau transgenic mouse model of Alzheimer's disease. Biochem Biophys Res Commun. 2013;430(2):670–675. doi:10.1016/j.bbrc.2012.11.093 23211594 10.1016/j.bbrc.2012.11.093

[57] Colacurcio DJ , Nixon RA . Disorders of lysosomal acidification‐The emerging role of v‐ATPase in aging and neurodegenerative disease. Ageing Res Rev. 2016;32:75–88. doi:10.1016/j.arr.2016.05.004 27197071 10.1016/j.arr.2016.05.004 PMC5112157

[58] Garbern JY , Neumann M , Trojanowski JQ , et al. A mutation affecting the sodium/proton exchanger, SLC9A6, causes mental retardation with tau deposition. Brain. 2010;133(Pt 5):1391–1402. doi:10.1093/brain/awq071 20395263 10.1093/brain/awq071 PMC2859154

[59] Cooper JM , Lathuiliere A , Su EJ , et al. SORL1 is a receptor for tau that promotes tau seeding. J Biol Chem. 2024;300(6):107313. doi:10.1016/j.jbc.2024.107313 38657864 10.1016/j.jbc.2024.107313 PMC11145553

[60] Rauch JN , Luna G , Guzman E , et al. LRP1 is a master regulator of tau uptake and spread. Nature. 2020;580(7803):381–385. doi:10.1038/s41586‐020‐2156‐5 32296178 10.1038/s41586-020-2156-5 PMC7687380

[61] Jiang L , Chakraborty P , Zhang L , et al. Chaperoning of specific tau structure by immunophilin FKBP12 regulates the neuronal resilience to extracellular stress. Sci Adv. 2023;9(5):eadd9789. doi:10.1126/sciadv.add9789 36724228 10.1126/sciadv.add9789 PMC9891691

[62] Zhang M , Cai F , Zhang S , Zhang S , Song W . Overexpression of ubiquitin carboxyl‐terminal hydrolase L1 (UCHL1) delays Alzheimer's progression in vivo. Sci Rep. 2014;4:7298. doi:10.1038/srep07298 25466238 10.1038/srep07298 PMC4252905

[63] Tumbarello DA , Waxse BJ , Arden SD , Bright NA , Kendrick‐Jones J , Buss F . Autophagy receptors link myosin VI to autophagosomes to mediate Tom1‐dependent autophagosome maturation and fusion with the lysosome. Nat Cell Biol. 2012;14(10):1024–1035. doi:10.1038/ncb2589 23023224 10.1038/ncb2589 PMC3472162

[64] Aschenbrenner L , Lee T , Hasson T . Myo6 facilitates the translocation of endocytic vesicles from cell peripheries. Mol Biol Cell. 2003;14(7):2728–2743. doi:10.1091/mbc.e02‐11‐0767 12857860 10.1091/mbc.E02-11-0767 PMC165672

[65] Feuillette S , Deramecourt V , Laquerriere A , et al. Filamin‐A and Myosin VI colocalize with fibrillary tau protein in Alzheimer's disease and FTDP‐17 brains. Brain Res. 2010;1345:182–189. doi:10.1016/j.brainres.2010.05.007 20460118 10.1016/j.brainres.2010.05.007

[66] Van Acker ZP , Bretou M , Annaert W . Endo‐lysosomal dysregulations and late‐onset Alzheimer's disease: impact of genetic risk factors. Mol Neurodegener. 2019;14(1):20. doi:10.1186/s13024‐019‐0323‐7 31159836 10.1186/s13024-019-0323-7 PMC6547588

[67] Higham JP , Malik BR , Buhl E , et al. Alzheimer's disease associated genes ankyrin and tau cause shortened lifespan and memory loss in drosophila. Front Cell Neurosci. 2019;13:260. doi:10.3389/fncel.2019.00260 31244615 10.3389/fncel.2019.00260 PMC6581016

[68] Dehmelt L , Halpain S . The MAP2/tau family of microtubule‐associated proteins. Genome Biol. 2005;6(1):204. doi:10.1186/gb‐2004‐6‐1‐204 15642108 10.1186/gb-2004-6-1-204 PMC549057

[69] Pensalfini A , Kim S , Subbanna S , et al. Endosomal dysfunction induced by directly overactivating Rab5 recapitulates prodromal and neurodegenerative features of Alzheimer's disease. Cell Rep. 2020;33(8):108420. doi:10.1016/j.celrep.2020.108420 33238112 10.1016/j.celrep.2020.108420 PMC7714675

[70] Lee JA , Gao FB . Roles of ESCRT in autophagy‐associated neurodegeneration. Autophagy. 2008;4(2):230–232. doi:10.4161/auto.5384 18094607 10.4161/auto.5384

[71] Razi M , Chan EY , Tooze SA . Early endosomes and endosomal coatomer are required for autophagy. J Cell Biol. 2009;185(2):305‐321. doi:10.1083/jcb.200810098 19364919 10.1083/jcb.200810098 PMC2700373

[72] Rusten TE , Stenmark H . How do ESCRT proteins control autophagy? J Cell Sci. 2009;122(Pt 13):2179–2183. doi:10.1242/jcs.050021 19535733 10.1242/jcs.050021

[73] Puri C , Renna M , Bento CF , Moreau K , Rubinsztein DC . Diverse autophagosome membrane sources coalesce in recycling endosomes. Cell. 2013;154(6):1285–1299. doi:10.1016/j.cell.2013.08.044 24034251 10.1016/j.cell.2013.08.044 PMC3791395

[74] Oyarzun JE , Lagos J , Vazquez MC , et al. Lysosome motility and distribution: relevance in health and disease. Biochim Biophys Acta Mol Basis Dis. 2019;1865(6):1076–1087. doi:10.1016/j.bbadis.2019.03.009 30904612 10.1016/j.bbadis.2019.03.009

[75] Udayar V , Chen Y , Sidransky E , Jagasia R . Lysosomal dysfunction in neurodegeneration: emerging concepts and methods. Trends Neurosci. 2022;45(3):184–199. doi:10.1016/j.tins.2021.12.004 35034773 10.1016/j.tins.2021.12.004 PMC8854344

