# Supplementary material for: Proteomic analysis links truncated tau to lysosome motility, autophagy, and endo‐lysosomal dysfunction
Source: Alzheimers Dement. 2025 Dec 15;21(12):e70977. doi: 10.1002/alz.70977 (PMC12706120; doi:10.1002/alz.70977)
Supplement: Supplementary file 1 — Supporting Information [file ALZ-21-e70977-s010.pdf]

A **Mouse brain: 4 mo.**

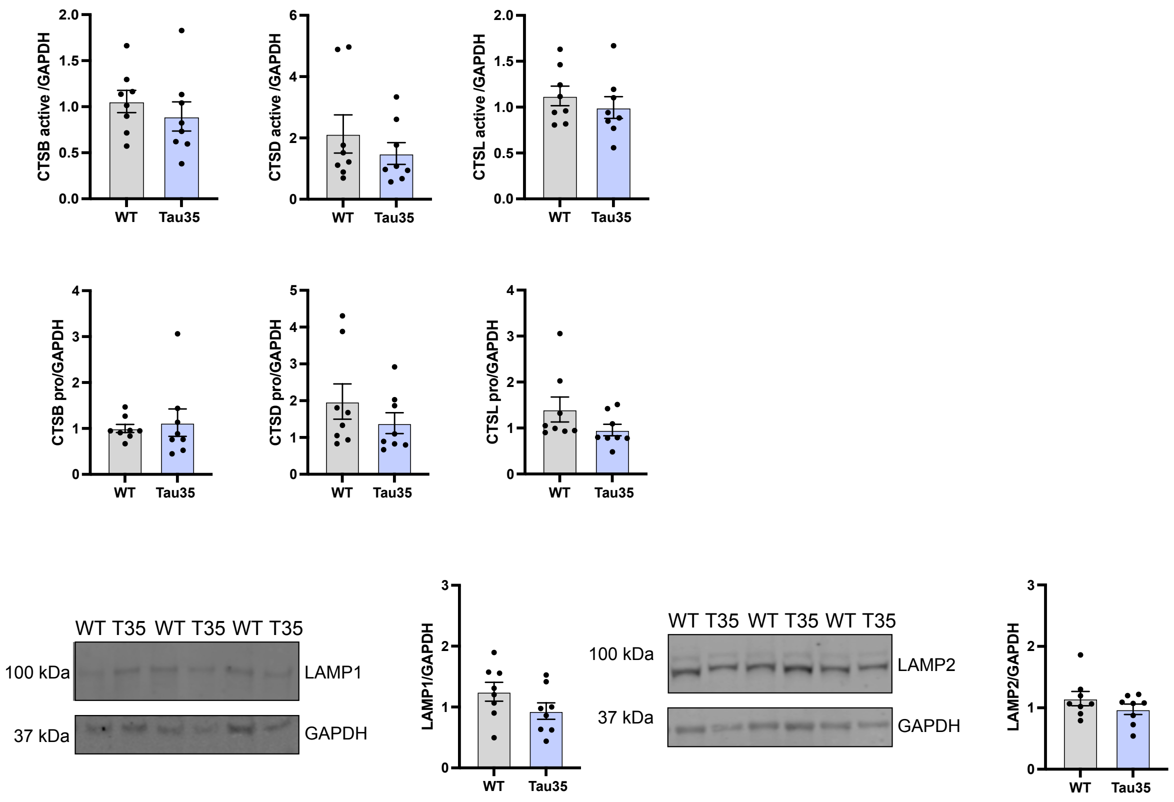

B **Mouse brain: 10 mo.**

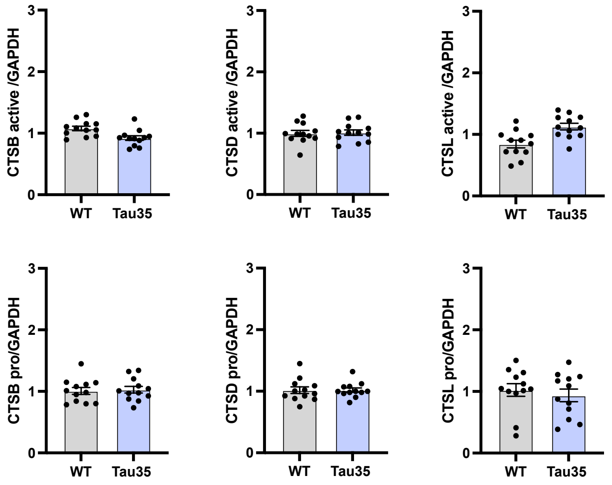

Supplementary Fig. 1: Pro-cathepsin and lysosomal marker expression across disease stages

(A-B) Western blots of total brain homogenates from WT and Tau35 mice aged 4 **(A)** and 10 **(B)** months respectively, were probed with antibodies to CTSB, CTSD, CTSL, LAMP1, LAMP2 and GAPDH. Quantification of the blots is shown in the graphs as mean ± SEM, n = 8-12 brains per group. Student t test. CTSB, Cathepsin B; CTSD, Cathepsin D; CTSL, Cathepsin L; LAMP1, lysosomal-associated membrane protein 1; LAMP2, lysosomal-associated membrane protein 2; GAPDH, glyceraldehyde 3-phosphate dehydrogenase; SEM, standard error of the mean; WT, wild type.
