# Supplementary material for: Proteomic analysis links truncated tau to lysosome motility, autophagy, and endo‐lysosomal dysfunction
Source: Alzheimers Dement. 2025 Dec 15;21(12):e70977. doi: 10.1002/alz.70977 (PMC12706120; doi:10.1002/alz.70977)
Supplement: Supplementary file 2 — Supporting Information [file ALZ-21-e70977-s004.pdf]

A

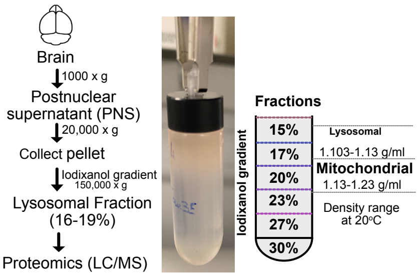

B

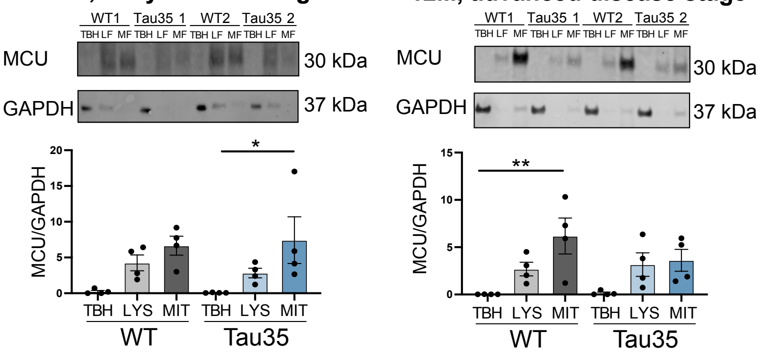

C Lysosomal Fractions - Discovery Analysis

Contrast	Total DP *	UP	DOWN
4M Lysosomal fractions: WT vs Tau35 (Early)	2005	148	136
10M Lysosomal fractions: WT vs Tau35 (Advanced)	2239	50	18

***DP:** detected proteins
p-cutoff: p->0.05, **fc-cutoff:** fc->0.5

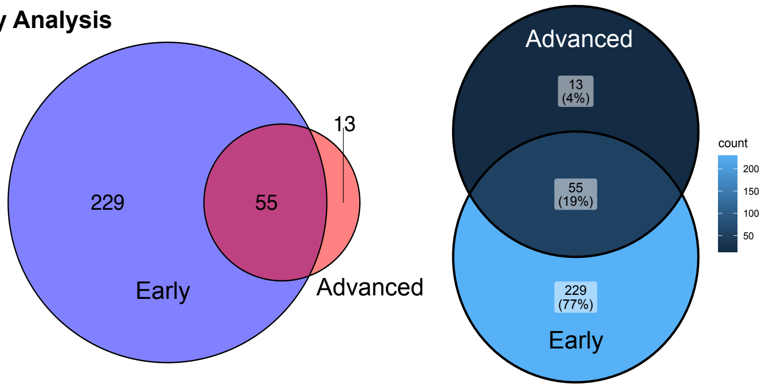

D Early: 4M Lysosomal fractions

Categories		Differentially expressed proteins
All Endo-exosomal genes	9	ehd3, epn2, myo6, rab5a, app, eps15l1, sorl1, rab5b, rab3c
Marking substrates for selective autophagy	6	atp5if1, slc25a5, vdac1, ube2d2, ube2n, nipsnap1
Mitophagy	5	ube2d2, ube2l3, ube2n, rab5a/b
Heat shock proteins	4	hspa12a, hspa4l, hspa9, hspa8
All autophagic genes	4	myo6, rab5a, rab5b, rab4b
Autophagophore membrane composition regulation	4	vamp2, vamp3, sar1a, rab4b
Rab proteins involved in autophagosome formation	3	rab5a, rab5b, rab4b
All PI3K genes	3	pi4ka, sacm1l, pitpna
Lysosome	3	chid1, atp6v1e1, atp6v1g1
Class 3 PI3K complex 1	3	rab5a, rab5b, exoc2

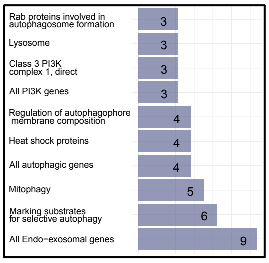

Advanced: 10M Lysosomal fractions

Categories		Differentially expressed proteins
V-type ATPase complex	2	atp6v1e1, atp6v1g1
All Lysosomal genes	2	atp6v1e1, atp6v1g1
Lysosome	2	atp6v1e1, atp6v1g1
mTORC1 pathway	2	atp6v1e1, atp6v1g1
Autophagophore membrane regulation	2	vamp2, vamp3
Regulation of lysosomal environment	2	atp6v1e1, atp6v1g1
Curated lysosomal Biogenesis	1	psap
Heat shock proteins	1	hspa4l
Docking and fusion	1	vamp3
All Endo-Exosomal genes	1	app

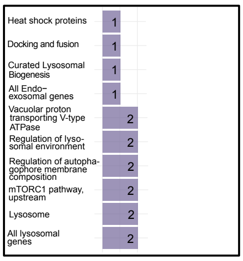

E

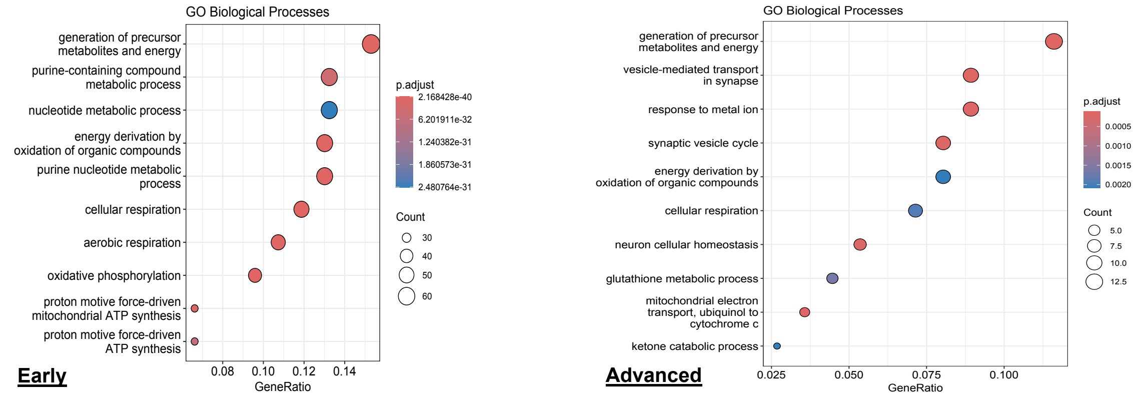

F

Contrast (Tau35 vs WT)	GO analysis top pathways per group	
Early: 4M Lysosomal fractions	GO:0006091 (67): generation of precursor metabolites and energy<br>GO:0015980 (57): energy derivation by oxidation of organic compounds<br>GO:0042776 (29): proton motive force-driven mitochondrial ATP synthesis	GO:0006119 (42): oxidative phosphorylation<br>GO:0045333 (52): cellular respiration<br>GO:0009060 (47): aerobic respiration
Advanced: 10M Lysosomal fractions	GO:0006091 (13): generation of precursor metabolites and energy<br>GO:0099003 (10): vesicle-mediated transport in synapse<br>GO:0006122 (4): mitochondrial electron transport	GO:0099504 (9): synaptic vesicle cycle<br>GO:0070050 (6): neuron cellular homeostasis<br>GO:0010038 (10): response to metal ion

Supplementary Fig. 2: Broad discovery analysis of lysosome protein dynamics in Tau35 mouse brains

(A) Schematic diagram of the applied workflow for subcellular fractionation of lysosomes from mouse brain. Representative images of the discontinuous iodixanol gradient showing enriched fractions from WT and Tau35 mouse brain samples. The positions of lysosomal and mitochondrial fractions on the gradient are indicated. **(B)** Western blots of total brain homogenates (TBH), lysosomal fractions (LF) and mitochondrial fractions (MF) from WT and Tau35 mice aged 4 and 10 months respectively, were probed with antibodies to MCU and GAPDH. Western blotting analysis of mouse brain extracts and the different subcellular fractions from WT and Tau35 brain reveal the enrichment of the mitochondrial marker protein MCU in MF. Quantification of the blots is shown in the graphs as mean ± SEM, n = 4 brains per group. Ordinary one-way ANOVA, $*P < 0.05$, $**P < 0.01$. MCU, mitochondrial calcium uniporter; GAPDH, glyceraldehyde 3-phosphate dehydrogenase; SEM, standard error of the mean; WT, wild type. **(C)** The limma package in R was used to analyse differentially expressed proteins (discovery analysis: p-cutoff: p->0.05, fc-cutoff: fc->0.5) from early and advanced sample cohorts. Table summarizing differentially expressed proteins between two cohorts, early and advanced, of wild-type (WT) and transgenic Tau35 mice. Venn diagrams illustrating protein alterations in lysosomal fractions from transgenic mice at 4 and 10 months. A total of 55 proteins are significantly altered across all datasets. **(D)** Tables and graphs highlight the key autophagy-lysosomal pathway (ALP) categories and proteins identified by comparing the limma-identified differentially expressed list of proteins (discovery analysis) at early and advanced disease stages with a curated list of autophagy and endo-lysosomal pathway-associated proteins. **(E)** Dot plots of the top 10 gene ontology (GO) terms in

the GO enrichment analysis for the early and advanced cohorts. **(F)** Table summarizing top pathways that are altered in early and advanced cohorts, including number of differentially expressed proteins that were detected per pathway.
