# Supplementary material for: Proteomic analysis links truncated tau to lysosome motility, autophagy, and endo‐lysosomal dysfunction
Source: Alzheimers Dement. 2025 Dec 15;21(12):e70977. doi: 10.1002/alz.70977 (PMC12706120; doi:10.1002/alz.70977)
Supplement: Supplementary file 3 — Supporting Information [file ALZ-21-e70977-s007.pdf]

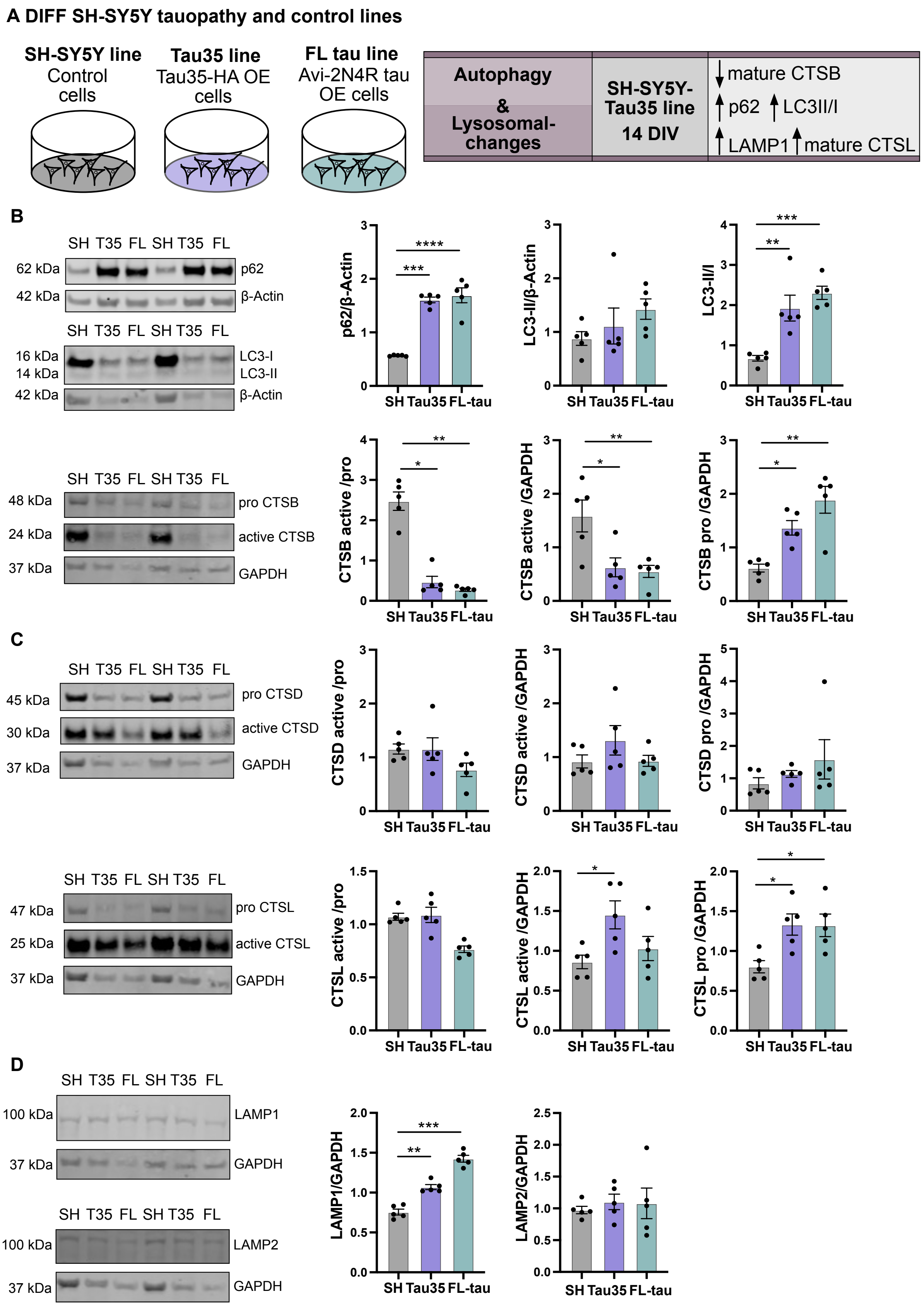
A DIFF SH-SY5Y tauopathy and control lines
SH-SY5Y line
Control cells
Tau35 line
Tau35-HA OE cells
FL tau line
Avi-2N4R tau OE cells
Autophagy & Lysosomal-changes
SH-SY5Y-Tau35 line 14 DIV
mature CTSB
p62
LC3II/I
LAMP1
mature CTSL
B
SH T35 FL SH T35 FL
62 kDa p62
42 kDa β-Actin
16 kDa LC3-I
14 kDa LC3-II
42 kDa β-Actin
48 kDa pro CTSB
24 kDa active CTSB
37 kDa GAPDH
p62/β-Actin
LC3-II/β-Actin
LC3-II/I
CTSB active /pro
CTSB active /GAPDH
CTSB pro /GAPDH
SH Tau35 FL-tau
C
45 kDa pro CTSD
30 kDa active CTSD
37 kDa GAPDH
CTSD active /pro
CTSD active /GAPDH
CTSD pro /GAPDH
47 kDa pro CTSL
25 kDa active CTSL
37 kDa GAPDH
CTSL active /pro
CTSL active /GAPDH
CTSL pro /GAPDH
D
100 kDa LAMP1
37 kDa GAPDH
100 kDa LAMP2
37 kDa GAPDH
LAMP1/GAPDH
LAMP2/GAPDH

Supplementary Fig. 3: Cathepsins and lysosomal marker expression in SH-SY5Y tau models

(A) Schematic illustrating the three differentiated SH-SY5Y cell lines; control SH-SY5Y cells, cells overexpressing (OE) Tau35-HA (referred to as Tau35) and cells overexpressing (OE) Avi-FL tau (referred to as FL-tau). Table summarizing changes in key lysosomal and autophagy markers (p62, LC3, LAMP1, LAMP2, CTSB, CTSD, CTSL) **(B-D)** Western blots of total cell lysates from control and tau-overexpressing SH-SY5Y cell lines were probed with antibodies to p62, LC3, CTSB, CTSD, CTSL, LAMP1, LAMP2, β-Actin and GAPDH. Quantification of the blots is shown in the graphs as mean ± SEM, *n* = 5 independent experiments. Two-way ANOVA, $^{*}P < 0.05$, $^{**}P < 0.01$, $^{***}P < 0.001$, $^{****}P < 0.0001$. p62/SQSTM1, Sequestosome-1; LC3, Microtubule-associated protein light chain 3; CTSB, Cathepsin B; CTSD, Cathepsin D; CTSL, Cathepsin L; LAMP1, lysosomal-associated membrane protein 1; LAMP2, lysosomal-associated membrane protein 2; GAPDH, glyceraldehyde 3-phosphate dehydrogenase; SEM, standard error of the mean.
