# Supplementary material for: Proteomic analysis links truncated tau to lysosome motility, autophagy, and endo‐lysosomal dysfunction
Source: Alzheimers Dement. 2025 Dec 15;21(12):e70977. doi: 10.1002/alz.70977 (PMC12706120; doi:10.1002/alz.70977)
Supplement: Supplementary file 4 — Supporting Information [file ALZ-21-e70977-s002.pdf]

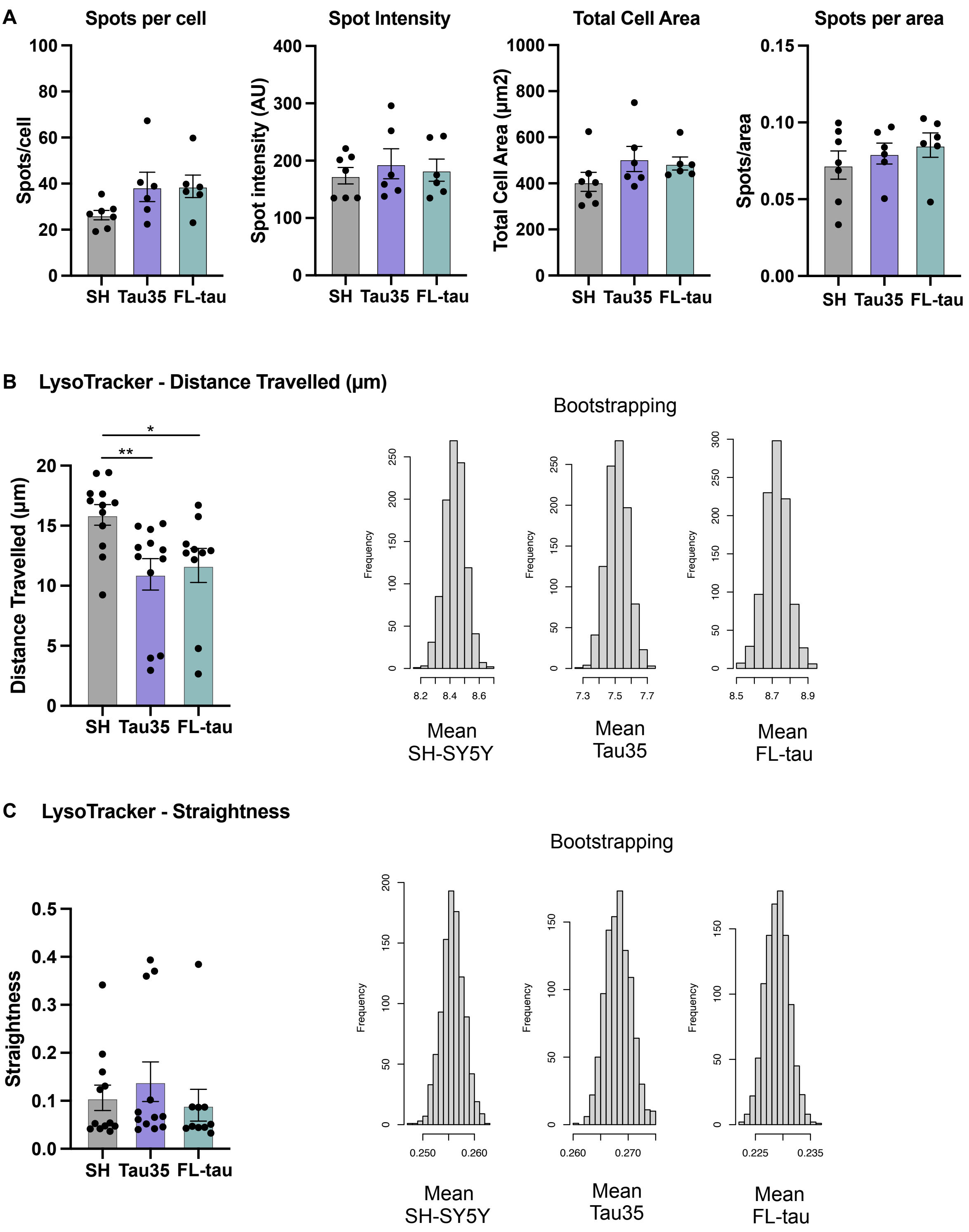

A
Spots per cell
Spots/cell
100
80
60
40
20
0
SH Tau35 FL-tau
Spot Intensity
Spot intensity (AU)
400
300
200
100
0
SH Tau35 FL-tau
Total Cell Area
Total Cell Area (µm2)
1000
800
600
400
200
0
SH Tau35 FL-tau
Spots per area
Spots/area
0.15
0.10
0.05
0.00
SH Tau35 FL-tau
B
LysoTracker - Distance Travelled (µm)
Distance Travelled (µm)
20
15
10
5
0
*
**
SH Tau35 FL-tau
Bootstrapping
Frequency
Mean
SH-SY5Y
Mean
Tau35
Mean
FL-tau
C
LysoTracker - Straightness
Straightness
0.5
0.4
0.3
0.2
0.1
0.0
SH Tau35 FL-tau
Bootstrapping
Frequency
Mean
SH-SY5Y
Mean
Tau35
Mean
FL-tau

D LysoTracker - Mean Velocity (µm/s)

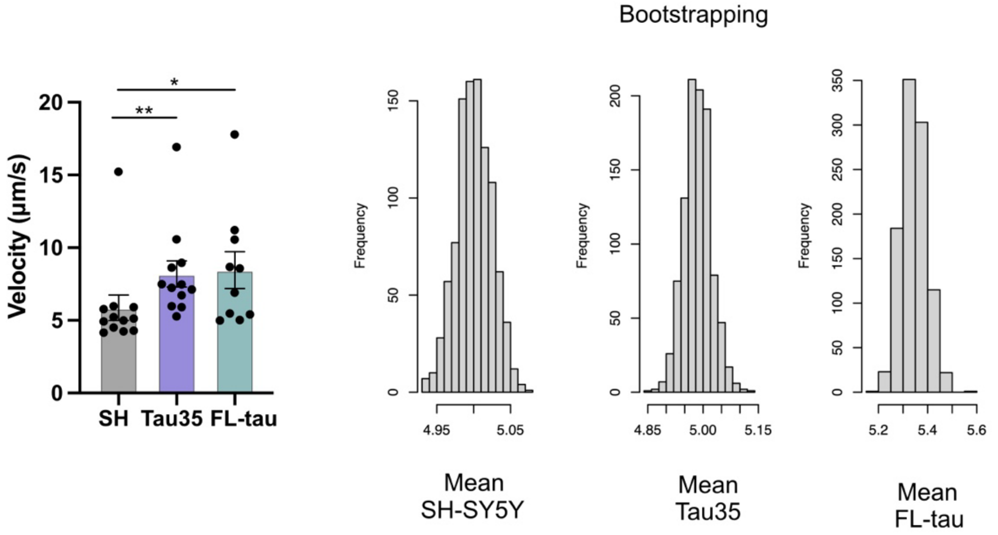

E LysoTracker - Mean Square Displacement (µm²)

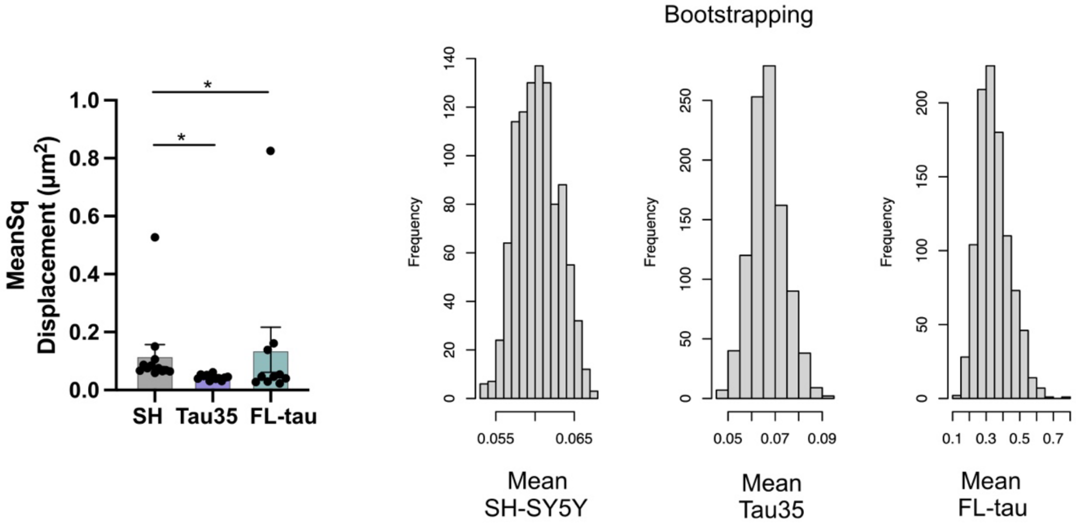

F LysoTracker - Mean Square Displacement (µm²)

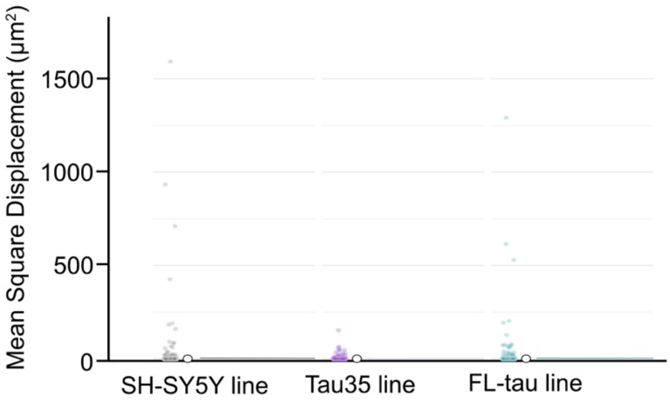

Supplementary Fig. 4: Bootstrapping analysis of lysosomal motility data for sampling distributions and robust comparisons

(A) Quantification of basic lysosome motility parameters, following lysotracker staining. Simple box plots of the number of spots per cell, spot intensity, total cell area and number of spots per area; AU: arbitrary units; Ordinary one-way ANOVA, SEM, standard error of the mean. **(B-E)** Simple box plots (left) and histograms (right) are shown to display the frequency distributions of lysosome motility parameters, Distance travelled (μm) **(B)**, Straightness **(C)**, Mean Velocity (μm/s) **(D)** and Mean Square Displacement (μm^2) **(E)**, for comparison between the three cell lines. The bootstrapping analysis was performed using random subsets of the data (1,000 bootstrap replicates for each parameter), which were derived from the original dataset containing 24,000–43,000 data points (spots) per line. For **B-F**: Quantification of the blots is shown in the graphs as mean ± SEM, n = 6-12 independent experiments; Kruskal-Wallis's test; $^{*}P < 0.05$, $^{**}P < 0.01$. **(F)** The original Raincloud plot for Mean Square Displacement (μm^2) is presented to accurately depict the full range of values in proportion across the different cell lines.
