# Supplementary material for: Proteomic analysis links truncated tau to lysosome motility, autophagy, and endo‐lysosomal dysfunction
Source: Alzheimers Dement. 2025 Dec 15;21(12):e70977. doi: 10.1002/alz.70977 (PMC12706120; doi:10.1002/alz.70977)
Supplement: Supplementary file 5 — Supporting Information [file ALZ-21-e70977-s006.pdf]

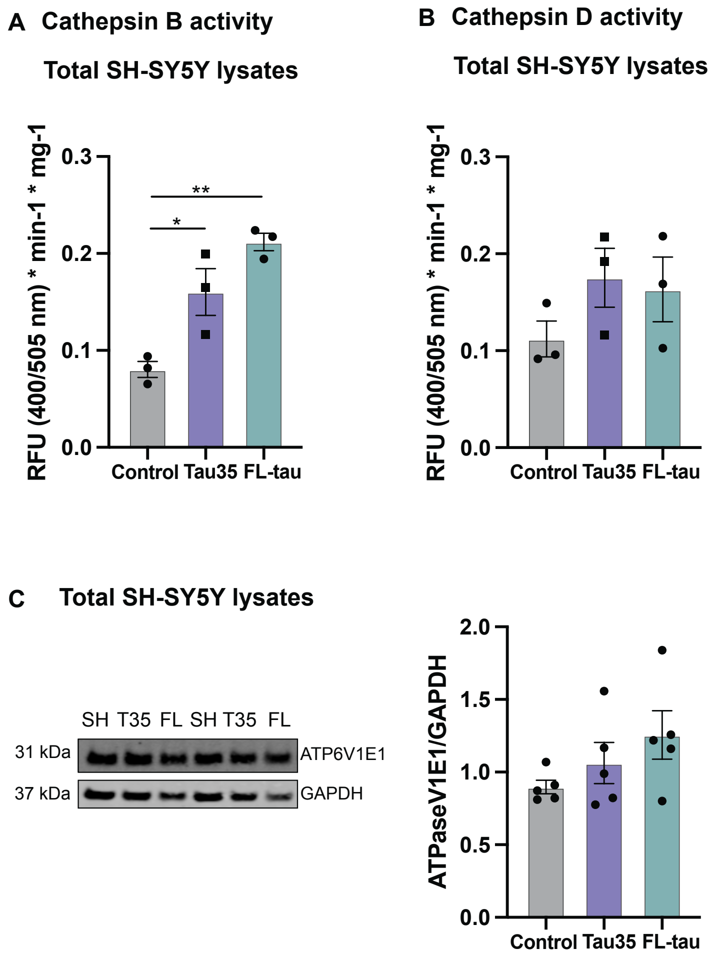

Supplementary Fig. 5: Assessment of cathepsin B and D activity and regulation of v-ATPase in SH-SY5Y tau models

Cathepsin B and D activity was measured in control, Tau35, and FL-tau differentiated SH-SY5Y cell lysates using commercial fluorometric assays. Activity is presented as blank corrected fluorescence, normalized to incubation time and total protein content. **(A)** Quantification of cathepsin B activity in SH-SY5Y cell lysates, shows a significant increase in cathepsin B activity in Tau35- and FL-tau-overexpressing lines. **(B)** Quantification of cathepsin D activity in SH-SY5Y cell lysates, shows a non-significant trend toward increased cathepsin D activity in Tau35- and FL-tau-overexpressing lines. **(C)** Lysosomal acidification was assessed via ATPase expression. Western blots

of SH-SY5Y cell lysates, were probed with antibodies to ATP6V1E1 and GAPDH and showed a modest increase in tau-overexpressing SH-SY5Y cells. Quantification of the blots is shown in the graphs as mean ± SEM, $n = 3$-5 samples per group; SEM, standard error of the mean. One-way ANOVA.
