# Supplementary material for: Proteomic analysis links truncated tau to lysosome motility, autophagy, and endo‐lysosomal dysfunction
Source: Alzheimers Dement. 2025 Dec 15;21(12):e70977. doi: 10.1002/alz.70977 (PMC12706120; doi:10.1002/alz.70977)
Supplement: Supplementary file 6 — Supporting Information [file ALZ-21-e70977-s008.pdf]

Supplemental file 1

Differential Expression Analysis of Lysosome-Enriched Brain Fractions – Differentially Expressed Proteins

Discovery Analysis (4M protein list)

Proteins	logFC	AveExpr	t	P.Value	adj.P.Val	B	Category
**Hprt1**	1.48265475	23.9755517	10.7238496	1.65097822452125E-05	0.02445837	3.40909547	upregulated proteins
**Glo1**	0.88921776	24.5264764	10.3034024	2.13703548473205E-05	0.02445837	3.20826276	upregulated proteins
**Atp6v1g1**	1.80128703	24.167866	8.18456405	9.21583025353879E-05	0.05202668	1.98868801	upregulated proteins
**S100a1**	0.81660577	26.8149709	8.01810459	0.000104754	0.05202668	1.87575835	upregulated proteins
**Kcnj10**	0.57032684	32.395901	7.34756403	0.000179646	0.05202668	1.3910004	upregulated proteins
**Ndufaf2**	-1.0069756	24.2984689	-7.2778964	0.000190431	0.05202668	1.33774588	downregulated proteins
**Rida**	0.76643256	25.68442	7.23929894	0.000196721	0.05202668	1.30799701	upregulated proteins
**Ppp1ca**	0.58788606	33.5578061	7.20083179	0.000203224	0.05202668	1.2781735	upregulated proteins
**Fabp3**	0.75358984	28.0613895	7.19309444	0.000204561	0.05202668	1.27215354	upregulated proteins
**Lrch2**	0.61208152	24.6448789	6.93293718	0.000255879	0.05408401	1.06553337	upregulated proteins
**Tusc3**	1.06537683	24.5783636	6.91507054	0.000259906	0.05408401	1.05103871	upregulated proteins
**Gsta4**	-0.6867246	19.9832786	-6.5846584	0.000348927	0.06210586	0.77568209	downregulated proteins
**Psap**	-1.1820599	24.9800101	-6.5077867	0.000374282	0.06210586	0.70958522	downregulated proteins
**Ank3**	1.39574022	24.0841377	6.491685	0.000379852	0.06210586	0.69564094	upregulated proteins
**Arf6**	0.67016129	26.3132387	6.37488627	0.000423171	0.06342825	0.59344817	upregulated proteins
**Sacm1l**	0.74457391	21.3244015	6.27735452	0.000463635	0.06342825	0.50669043	upregulated proteins
**Aco1**	0.6340852	21.851129	6.26047697	0.00047107	0.06342825	0.49154427	upregulated proteins
**App**	0.61887613	25.1380994	6.13420733	0.000531154	0.06754514	0.37696742	upregulated proteins
**Dclk1**	-0.8340945	25.1093881	-6.0055858	0.000601368	0.07244906	0.25793787	downregulated proteins
**Chp1**	0.54454911	28.6907657	5.77302955	0.000756447	0.07550888	0.0366386	upregulated proteins
**Gsr**	-0.5753545	31.0372655	-5.7598019	0.000766533	0.07550888	0.0238114	downregulated proteins
**Got1**	-0.8774215	27.573672	-5.7436705	0.000779037	0.07550888	0.00813298	downregulated proteins
**Cds1**	0.53500724	22.7326039	5.70218735	0.000812258	0.07550888	-0.0323644	upregulated proteins
**Ppp2r5c**	0.71957055	26.1589525	5.63901063	0.000865947	0.07550888	-0.094538	upregulated proteins
**Rab5a**	-0.6320557	23.4053561	-5.6179465	0.000884724	0.07550888	-0.1154022	downregulated proteins
**Dnajc6**	1.17516633	23.1239242	5.61138158	0.000890668	0.07550888	-0.1219185	upregulated proteins
**Tpt1**	-0.5410861	28.1665027	-5.5046935	0.00099383	0.07769799	-0.2287422	downregulated proteins
**Timm10**	0.94194164	22.7471944	5.48561804	0.001013651	0.07769799	-0.2480265	upregulated proteins
**Cbarp**	0.87567699	26.2610788	5.48118338	0.001018322	0.07769799	-0.2525177	upregulated proteins
**Ist1**	-0.6975562	29.6554428	-5.382166	0.001129157	0.08337551	-0.3535956	downregulated proteins
**Uqcrh**	0.5037877	25.412371	5.32711556	0.001196574	0.08444618	-0.4104553	upregulated proteins
**Cct2**	0.71745459	26.1063589	5.31077598	0.001217442	0.08444618	-0.4274238	upregulated proteins

**Rab18**	-0.6651339	26.0305222	-5.1683887	0.001417524	0.08725505	-0.5770853	downregulated proteins
**Calb2**	-0.7080722	27.191278	-5.1390527	0.001463161	0.08725505	-0.6083227	downregulated proteins
**Vamp2**	-0.6873281	27.5600146	-5.1311363	0.001475755	0.08725505	-0.6167758	downregulated proteins
**Pdcd6**	1.50635791	24.0803382	5.10102782	0.001524772	0.08725505	-0.6490181	upregulated proteins
**Ncald**	0.77014992	25.0371729	4.99797915	0.001706791	0.09085686	-0.7604805	upregulated proteins
**Sez6l2**	0.5880655	22.1403674	4.97292817	0.001754617	0.09127995	-0.7878378	upregulated proteins
**Lgi3**	-0.8686743	29.0507037	-4.9292856	0.001841542	0.09264566	-0.8357432	downregulated proteins
**Uso1**	0.63090223	25.393019	4.8582819	0.001993396	0.09264566	-0.9143499	upregulated proteins
**Akr1b1**	-0.8370796	28.9094793	-4.8480056	0.002016506	0.09264566	-0.9257953	downregulated proteins
**Plec**	0.91209025	24.4801612	4.84772142	0.002017149	0.09264566	-0.926112	upregulated proteins
**Gstm1**	-0.5869978	24.2442124	-4.8370675	0.002041432	0.09264566	-0.9379969	downregulated proteins
**Kcna1**	-0.6697445	27.2864501	-4.82721	0.002064189	0.09264566	-0.94901	downregulated proteins
**Hcn2**	-0.8912277	27.5315521	-4.7218579	0.00232584	0.09933821	-1.0677159	downregulated proteins
**Pdp1**	-1.0725089	24.2240072	-4.70291	0.002376702	0.09933821	-1.0892608	downregulated proteins
**Lgi2**	-0.5710203	22.216167	-4.69799	0.00239011	0.09933821	-1.0948649	downregulated proteins
**Atp5if1**	-0.6182418	22.3684778	-4.6683257	0.002472751	0.09933821	-1.1287391	downregulated proteins
**Arpc4**	0.82177925	24.1548782	4.65840636	0.002501089	0.09933821	-1.1400989	upregulated proteins
**Ndrg4**	-0.7023022	28.7830597	-4.6301474	0.002583814	0.09933821	-1.1725512	downregulated proteins
**Smpd3**	-0.6360064	31.0260403	-4.6284417	0.002588904	0.09933821	-1.1745143	downregulated proteins
**Uchl1**	-1.0415454	24.4911962	-4.6106582	0.002642633	0.09933821	-1.19501	downregulated proteins
**Ube2l3**	-0.9670504	21.9990242	-4.6091382	0.002647283	0.09933821	-1.1967643	downregulated proteins
**Rab2a**	1.17285903	24.7912644	4.56938782	0.002772152	0.10057711	-1.242778	upregulated proteins
**Krt73**	0.76924148	24.2245667	4.54815929	0.002841502	0.10057711	-1.2674595	upregulated proteins
**Tkt**	-0.5966042	29.2760678	-4.5351016	0.002885112	0.10057711	-1.2826785	downregulated proteins
**Psma6**	-0.6724336	27.9566507	-4.4862483	0.003054971	0.10057711	-1.3398708	downregulated proteins
**Vamp3**	1.37668281	23.6340417	4.46510189	0.003131903	0.10057711	-1.3647505	upregulated proteins
**DSCC1**	0.58750931	25.421489	4.4532578	0.003175928	0.10057711	-1.3787184	upregulated proteins
**Pfn1**	0.55850415	19.6636186	4.43432297	0.003247737	0.10057711	-1.4010971	upregulated proteins
**Vat1**	0.79243311	24.7966748	4.41318603	0.003330026	0.10057711	-1.4261492	upregulated proteins
**Ldhb**	-0.6517663	24.1816152	-4.4025669	0.003372236	0.10057711	-1.4387635	downregulated proteins
**Pde1b**	0.67824973	24.6630713	4.39352121	0.003408658	0.10057711	-1.4495237	upregulated proteins
**Nrxn2**	-0.7602648	24.5542851	-4.3930344	0.00341063	0.10057711	-1.4501032	downregulated proteins
**Ca8**	0.72145164	21.5420139	4.39072499	0.003420004	0.10057711	-1.4528526	upregulated proteins
**Arpc2**	0.78633532	22.3066835	4.38894051	0.003427267	0.10057711	-1.4549778	upregulated proteins
**Ppp2r1a**	-0.7669361	28.8571869	-4.3592343	0.003550711	0.10265279	-1.4904332	downregulated proteins
**Anxa5**	1.08617293	22.2627323	4.34122379	0.00362794	0.10265279	-1.5120012	upregulated proteins
**Atp5me**	0.74422764	20.9637672	4.3401653	0.003632536	0.10265279	-1.5132705	upregulated proteins
**Slc39a12**	0.6039702	29.4809369	4.31288058	0.003753259	0.10325773	-1.5460526	upregulated proteins
**Slc38a3**	1.09626222	23.8244998	4.29395118	0.003839613	0.10325773	-1.568869	upregulated proteins
**Kcnab2**	-0.6836487	23.4307202	-4.2879963	0.003867231	0.10325773	-1.576059	downregulated proteins
**Marcksl1**	-0.9715984	22.6324844	-4.2853668	0.003879495	0.10325773	-1.5792358	downregulated proteins
**Atp6v1e1**	0.6790977	26.0085288	4.26967857	0.003953566	0.10401969	-1.5982132	upregulated proteins
**Ddt**	-0.9920749	20.0750888	-4.2264689	0.004165766	0.10416692	-1.6506943	downregulated proteins

**Hpcal4**	-0.6108881	25.4525551	-4.2178842	0.004209402	0.10416692	-1.6611579	downregulated proteins
**Pex5l**	-0.9850309	22.0339253	-4.2053528	0.004274007	0.10416692	-1.6764541	downregulated proteins
**Hspa4l**	-0.6134817	26.4501494	-4.2041687	0.004280168	0.10416692	-1.6779009	downregulated proteins
**Anxa6**	-1.1408992	21.4818466	-4.2027765	0.004287423	0.10416692	-1.6796021	downregulated proteins
**Pfn2**	0.50220858	26.9547177	4.16636098	0.004482114	0.10416692	-1.7242156	upregulated proteins
**Mpp3**	-0.8555979	25.5038185	-4.1521823	0.004560533	0.10416692	-1.7416457	downregulated proteins
**Nectin1**	-0.6644981	29.2179201	-4.1379985	0.004640495	0.10416692	-1.7591154	downregulated proteins
**Acyp1**	-0.5927846	26.166695	-4.1361893	0.004650805	0.10416692	-1.7613461	downregulated proteins
**Praf2**	0.53764647	28.251948	4.1332966	0.004667342	0.10416692	-1.7649139	upregulated proteins
**Napa**	1.04867358	20.730417	4.11547639	0.004770652	0.10416692	-1.7869234	upregulated proteins
**Cyc1**	1.0178708	24.1474367	4.11519766	0.004772287	0.10416692	-1.7872681	upregulated proteins
**Zfyve28**	0.74000975	25.8301674	4.09037906	0.00492044	0.10416692	-1.8180097	upregulated proteins
**Nbea**	-0.5915865	29.3197876	-4.089531	0.004925591	0.10416692	-1.819062	downregulated proteins
**Gpi**	1.31587344	22.6933896	4.0695012	0.005048988	0.10416692	-1.8439487	upregulated proteins
**Gnai2**	-0.8903206	27.7041308	-4.0669587	0.005064893	0.10416692	-1.8471124	downregulated proteins
**Ndufab1**	0.55996893	26.0546015	4.05923495	0.005113548	0.10416692	-1.8567299	upregulated proteins
**Calb1**	-0.8471172	21.2520204	-4.0477105	0.005187104	0.10416692	-1.8710982	downregulated proteins
**Trpv2**	-0.6214032	27.8360097	-4.039793	0.005238311	0.10416692	-1.880982	downregulated proteins
**Pgam1**	1.08165902	22.7614465	4.0074309	0.005453463	0.10528471	-1.9214882	upregulated proteins
**Lmbrd2**	-0.6127329	27.4905644	-3.9934858	0.005549147	0.10528471	-1.9389953	downregulated proteins
**Slc12a2**	0.89747707	21.6402576	3.98979226	0.005574799	0.10528471	-1.9436377	upregulated proteins
**Oxct1**	-0.7798032	27.7823803	-3.9771965	0.005663257	0.10528471	-1.9594856	downregulated proteins
**Apmap**	-0.6167554	29.3098344	-3.9737739	0.005687558	0.10528471	-1.9637964	downregulated proteins
**Ugp2**	-0.6948793	27.2145094	-3.9628211	0.005766099	0.10528471	-1.9776043	downregulated proteins
**Capza2**	0.66116126	24.7420976	3.95079636	0.005853697	0.10528471	-1.9927859	upregulated proteins
**Ephb3**	0.84795328	21.367996	3.91493612	0.006123712	0.10650789	-2.0381994	upregulated proteins
**Ndufs4**	0.8322696	23.5237935	3.91229232	0.006144153	0.10650789	-2.0415557	upregulated proteins
**Uqcrfs1**	-0.8270405	24.2077068	-3.9058951	0.006193922	0.10650789	-2.0496816	downregulated proteins
**Idh1**	-0.7314133	29.9971087	-3.8953807	0.006276685	0.10650789	-2.0630518	downregulated proteins
**Lactb**	0.51628396	27.9357334	3.86971296	0.006483854	0.10895989	-2.0957652	upregulated proteins
**Rab5b**	-0.6670558	28.2768898	-3.8492285	0.006654559	0.10895989	-2.1219481	downregulated proteins
**Nrcam**	-0.6324692	28.6558368	-3.8491203	0.006655473	0.10895989	-2.1220865	downregulated proteins
**Hmox2**	0.59480531	25.4293949	3.84808717	0.006664213	0.10895989	-2.1234089	upregulated proteins
**Adsl**	-0.542735	29.7295738	-3.8394761	0.006737551	0.10904645	-2.1344368	downregulated proteins
**Ckb**	0.65780871	24.4612015	3.82998718	0.006819389	0.10904645	-2.1466028	upregulated proteins
**Osbpl1a**	0.67245746	25.6545822	3.81595151	0.00694244	0.10904645	-2.1646244	upregulated proteins
**Lrpap1**	-0.8139047	25.5137265	-3.8156604	0.006945018	0.10904645	-2.1649985	downregulated proteins
**Mdh1**	-0.5423317	29.8590478	-3.8091452	0.007002983	0.10904645	-2.1733747	downregulated proteins
**Rad23b**	0.61479195	24.7224829	3.79502926	0.007130394	0.11028021	-2.191546	upregulated proteins
**Svip**	0.52522811	30.2649903	3.77144744	0.007348928	0.11085336	-2.2219723	upregulated proteins
**Ehd3**	-0.5550309	29.4685505	-3.7597014	0.007460499	0.11085336	-2.2371601	downregulated proteins
**Gdi1**	-0.5326956	24.4144877	-3.7540465	0.007514871	0.11085336	-2.2444797	downregulated proteins
**Nnt**	-0.6867434	27.1578973	-3.7508042	0.00754624	0.11085336	-2.2486786	downregulated proteins

**Mcam**	0.51788917	26.5066716	3.72781865	0.007772766	0.11085336	-2.2784932	upregulated proteins
**Vdac1**	-1.2054104	21.5370307	-3.7136009	0.007916591	0.11085336	-2.2969761	downregulated proteins
**Tppp3**	-0.5749736	27.9498256	-3.712333	0.007929557	0.11085336	-2.2986258	downregulated proteins
**Atp5pf**	1.14875843	22.3920259	3.71091035	0.007944133	0.11085336	-2.3004772	upregulated proteins
**Itfg1**	0.54199775	28.7035595	3.70731415	0.007981109	0.11085336	-2.3051587	upregulated proteins
**Rev1**	-0.9232934	21.5288733	-3.6998518	0.008058437	0.11085336	-2.3148793	downregulated proteins
**Endod1**	-0.5442246	28.6002902	-3.6988988	0.008068372	0.11085336	-2.3161214	downregulated proteins
**Aqp4**	-0.5370287	25.166431	-3.694211	0.008117431	0.11085336	-2.3222328	downregulated proteins
**Adam11**	-0.6310452	27.2345267	-3.6863859	0.008200051	0.11085336	-2.332442	downregulated proteins
**Mag**	-0.565855	26.5231436	-3.6824876	0.008241552	0.11085336	-2.3375314	downregulated proteins
**Prps1**	1.22983171	23.53826	3.66594781	0.008420194	0.11158678	-2.359151	upregulated proteins
**Lamp5**	0.75548814	24.6255888	3.65559387	0.008534168	0.11158678	-2.372706	upregulated proteins
**Sar1a**	-0.5372479	29.3352754	-3.6536683	0.008555549	0.11158678	-2.3752287	downregulated proteins
**Rap1gds1**	-0.6081903	29.4923423	-3.6513587	0.00858127	0.11158678	-2.3782553	downregulated proteins
**Psmd6**	0.52350875	28.7148571	3.64633826	0.008637474	0.11158678	-2.384837	upregulated proteins
**Cadps**	0.65589016	26.2093298	3.64344587	0.008670035	0.11158678	-2.3886306	upregulated proteins
**Cab39**	0.98373341	23.2495091	3.64279804	0.008677347	0.11158678	-2.3894804	upregulated proteins
**Kcnc3**	-0.6219098	30.5292328	-3.629337	0.008830796	0.11229829	-2.4071535	downregulated proteins
**Etfdh**	-0.7561109	26.2368074	-3.6205116	0.008933001	0.11253002	-2.4187552	downregulated proteins
**Npepps**	-0.6412135	27.6906142	-3.6007706	0.009166307	0.11403086	-2.4447486	downregulated proteins
**Ndufb8**	0.50002273	25.9400215	3.5885873	0.009313594	0.11501283	-2.4608197	upregulated proteins
**Pura**	1.1258595	19.6750244	3.58481754	0.009359687	0.11501283	-2.4657968	upregulated proteins
**Agpat3**	-0.8381432	23.8775397	-3.5818634	0.00939598	0.11501283	-2.4696986	downregulated proteins
**Rufy3**	-0.5752557	26.0731899	-3.5674555	0.009575195	0.11658309	-2.4887468	downregulated proteins
**Psat1**	0.67843887	24.9429422	3.55538918	0.009728141	0.11702908	-2.5047228	upregulated proteins
**Gdi2**	-1.0035695	23.4059239	-3.5431468	0.009886034	0.11702908	-2.5209537	downregulated proteins
**Tnc**	0.57535477	24.2182701	3.54203501	0.00990051	0.11702908	-2.5224288	upregulated proteins
**Cntnap2**	-0.6569151	27.8897705	-3.5374534	0.009960409	0.11702908	-2.5285095	downregulated proteins
**Emc1**	0.66214944	22.9813817	3.52968085	0.010062925	0.11752059	-2.538832	upregulated proteins
**Itgav**	1.26924843	22.9278385	3.51006348	0.010326796	0.11915364	-2.5649244	upregulated proteins
**Ppp1r7**	-0.5019489	23.8874674	-3.498412	0.010487063	0.11915364	-2.5804479	downregulated proteins
**Ndufb9**	1.48077869	21.5462391	3.48933349	0.010613807	0.11915364	-2.5925568	upregulated proteins
**Psma4**	0.56260426	28.1689796	3.48748302	0.010639844	0.11915364	-2.5950264	upregulated proteins
**Cend1**	1.65828206	23.8296867	3.47833023	0.010769651	0.11915364	-2.6072487	upregulated proteins
**Nipsnap1**	-1.221358	24.1899699	-3.4755886	0.010808867	0.11915364	-2.6109121	downregulated proteins
**Them4**	1.76207438	22.7022354	3.45811928	0.011062405	0.11981767	-2.6342797	upregulated proteins
**Prrt1**	0.69989154	23.9577341	3.45576003	0.011097137	0.11981767	-2.6374388	upregulated proteins
**Arhgdia**	0.57572522	27.4169954	3.41108582	0.011777569	0.12310034	-2.6974054	upregulated proteins
**Eps15l1**	-0.6286692	26.1301163	-3.394264	0.012045361	0.12475942	-2.7200568	downregulated proteins
**Ube2n**	0.78144195	21.347346	3.37787142	0.012312659	0.1263842	-2.7421672	upregulated proteins
**Eif5a**	-0.624961	28.3427823	-3.3478554	0.01281885	0.12983339	-2.7827466	downregulated proteins
**Lrp1**	1.11769815	20.9665289	3.30491789	0.013582361	0.13517402	-2.8410016	upregulated proteins
**Me1**	-0.6134638	28.1877574	-3.2926845	0.013808762	0.13683228	-2.857643	downregulated proteins

**Atp1b3**	-0.5675099	28.6316218	-3.2655033	0.014326473	0.14014229	-2.8946862	downregulated proteins
**Tpi1**	-0.5835155	29.3117358	-3.2503304	0.014624517	0.14062518	-2.9154047	downregulated proteins
**Psmb1**	-0.5447699	29.599437	-3.249125	0.014648479	0.14062518	-2.9170518	downregulated proteins
**Kpnb1**	0.57249032	26.1796638	3.24668492	0.014697114	0.14062518	-2.9203867	upregulated proteins
**Creld1**	-0.5340874	27.1868876	-3.24617	0.0147074	0.14062518	-2.9210906	downregulated proteins
**Itgb1**	1.47949672	21.6000895	3.24446543	0.014741504	0.14062518	-2.9234208	upregulated proteins
**Etfb**	0.82379363	23.7273361	3.24431843	0.014744449	0.14062518	-2.9236218	upregulated proteins
**Sh3bgrl3**	0.93620302	24.6087334	3.22916611	0.015051437	0.14195782	-2.9443518	upregulated proteins
**Alb**	-0.6140064	26.0484596	-3.2267613	0.015100786	0.14195782	-2.9476444	downregulated proteins
**Aars1**	-0.6226111	22.5020521	-3.2209403	0.015220964	0.14195782	-2.9556173	downregulated proteins
**Timm9**	-0.634639	29.9644668	-3.2194776	0.015251323	0.14195782	-2.9576214	downregulated proteins
**Trpc3**	-0.6117487	28.7523035	-3.2011395	0.015637506	0.14310023	-2.9827687	downregulated proteins
**Pea15**	-0.626578	27.5902955	-3.194766	0.015774174	0.14310023	-2.9915182	downregulated proteins
**Ywhaq**	0.52120322	24.9068393	3.19136847	0.015847551	0.14310023	-2.9961843	upregulated proteins
**Basp1**	0.65609816	23.8599123	3.18218301	0.01604777	0.14310023	-3.0088061	upregulated proteins
**Slc8a1**	0.50059375	24.7711454	3.18024837	0.016090285	0.14310023	-3.0114658	upregulated proteins
**Fundc2**	-1.1604113	26.2147083	-3.1624145	0.016487931	0.14424435	-3.0360037	downregulated proteins
**Csnk2a1**	-0.5342361	27.7472747	-3.1612311	0.01651469	0.14424435	-3.0376334	downregulated proteins
**Mtfp1**	-1.0375294	21.7447425	-3.1611011	0.016517631	0.14424435	-3.0378124	downregulated proteins
**Zmpste24**	-0.815762	24.3286043	-3.1395436	0.017013374	0.14500449	-3.0675258	downregulated proteins
**Crym**	0.51546647	29.9293483	3.13924902	0.017020257	0.14500449	-3.0679322	upregulated proteins
**Rexo2**	-0.6209731	22.3833367	-3.1383741	0.017040719	0.14500449	-3.0691393	downregulated proteins
**Myo6**	1.54710877	23.2549639	3.13472681	0.017126305	0.14519301	-3.0741721	upregulated proteins
**Bcap31**	0.69422854	22.8798697	3.12967407	0.017245632	0.14566513	-3.0811469	upregulated proteins
**Nsdhl**	-0.733907	25.5454747	-3.1265598	0.017319621	0.14568756	-3.0854471	downregulated proteins
**Gnas**	-0.5043987	29.2696707	-3.1193506	0.017492209	0.14568756	-3.095406	downregulated proteins
**Prrt2**	-0.5878173	21.40244	-3.1156685	0.017581067	0.14580819	-3.1004948	downregulated proteins
**Slc9a6**	-1.201937	25.8919655	-3.108631	0.017752244	0.14623364	-3.1102248	downregulated proteins
**Uggt1**	0.62803202	23.9495455	3.10274365	0.017896815	0.14623364	-3.1183688	upregulated proteins
**Mtatp8**	-0.9011461	24.4371739	-3.1010227	0.017939314	0.14623364	-3.1207502	downregulated proteins
**Uqcrc2**	-0.5980995	30.1955702	-3.0940423	0.018112799	0.14623364	-3.1304124	downregulated proteins
**Mtcl3**	0.59080738	22.815713	3.09249404	0.018151519	0.14623364	-3.1325562	upregulated proteins
**Camkv**	1.12941544	23.8445987	3.08245981	0.018404627	0.14647242	-3.1464563	upregulated proteins
**Lonp1**	-0.5494978	25.6090922	-3.0818093	0.018421166	0.14647242	-3.1473578	downregulated proteins
**Hspa9**	-0.8152296	26.5408346	-3.0815001	0.018429033	0.14647242	-3.1477863	downregulated proteins
**Dcps**	1.18926942	20.9786022	3.07603147	0.018568758	0.14707228	-3.1553669	upregulated proteins
**Eno2**	-0.8562782	20.9843183	-3.0628791	0.018909463	0.14925435	-3.1736116	downregulated proteins
**Hycc2**	2.28644549	21.7644682	3.05168618	0.019204665	0.15012594	-3.1891523	upregulated proteins
**Dlg2**	-0.5825207	27.1814717	-3.0463519	0.019347077	0.15063082	-3.1965631	downregulated proteins
**Pgrmc2**	0.59799296	25.1581211	3.03928208	0.019537565	0.15098281	-3.2063896	upregulated proteins
**Ndufs2**	-0.6205498	28.1900791	-3.0386904	0.019553598	0.15098281	-3.2072122	downregulated proteins
**Ptpa**	-0.6481831	24.9552537	-3.0373427	0.019590168	0.15098281	-3.2090861	downregulated proteins
**Exoc2**	0.64171435	23.6882598	3.0278588	0.019849589	0.15195889	-3.2222777	upregulated proteins

**Cd9**	0.97201851	22.6936838	3.02211667	0.020008436	0.15215717	-3.230269	upregulated proteins
**Gstk1**	0.50555804	27.1508788	3.00084537	0.020608778	0.1556881	-3.2599006	upregulated proteins
**Taldo1**	-0.5580091	27.7321038	-2.9955168	0.020762153	0.15633081	-3.2673303	downregulated proteins
**Syt2**	-0.528018	29.1861083	-2.9715718	0.021466519	0.15991055	-3.3007502	downregulated proteins
**Timm50**	-0.602443	27.2765111	-2.9526178	0.022042076	0.16163023	-3.3272415	downregulated proteins
**Atcay**	-0.5317845	28.6953242	-2.9468675	0.022219917	0.16197895	-3.335285	downregulated proteins
**Atp2a3**	-0.8222782	23.6624202	-2.9430513	0.022338779	0.16199736	-3.3406245	downregulated proteins
**Ppia**	1.10397588	22.2358438	2.94224483	0.022363986	0.16199736	-3.3417532	upregulated proteins
**Psmc2**	-0.807544	23.670047	-2.9357005	0.022569646	0.16297136	-3.3509135	downregulated proteins
**Psmd8**	0.60577887	24.1507808	2.91348081	0.023283021	0.16654636	-3.3820424	upregulated proteins
**Prdx2**	-0.5166998	27.502797	-2.8955057	0.023877614	0.1692349	-3.4072552	downregulated proteins
**Ktn1**	0.58335807	26.7302483	2.89321458	0.023954549	0.1692349	-3.4104707	upregulated proteins
**Ube2d2**	0.52620264	26.836314	2.86115819	0.025058975	0.17280242	-3.4555044	upregulated proteins
**Pi4ka**	-0.5857924	28.1871088	-2.8561408	0.025236658	0.17347361	-3.4625602	downregulated proteins
**Hspa12a**	0.60911152	26.2284845	2.85400017	0.025312871	0.17347653	-3.465571	upregulated proteins
**Hspd1**	-0.7226156	25.3930766	-2.8130937	0.026816957	0.17771215	-3.5231704	downregulated proteins
**Lingo1**	0.90402743	20.9195797	2.81221582	0.026850251	0.17771215	-3.5244077	upregulated proteins
**Epn2**	0.74457709	23.9717406	2.80920124	0.026964915	0.17787519	-3.5286572	upregulated proteins
**Grm5**	-0.6603754	23.9283484	-2.8055206	0.027105615	0.17828952	-3.5338465	downregulated proteins
**Pitpna**	0.67302889	19.5153978	2.79992106	0.027321156	0.17919234	-3.5417429	upregulated proteins
**Cnot11**	-0.5331357	23.3749328	-2.7777707	0.028191658	0.18211057	-3.572999	downregulated proteins
**Rab4b**	-0.6509078	24.7650478	-2.7761152	0.028257879	0.18211057	-3.5753362	downregulated proteins
**Uba1**	0.66662987	23.3312121	2.77449107	0.028323007	0.18211057	-3.5776293	upregulated proteins
**Itm2b**	-13.552999	15.7889577	-2.7692724	0.02853335	0.18294912	-3.5849988	downregulated proteins
**Nefh**	0.53598474	26.4302971	2.76504161	0.028705081	0.18353612	-3.5909744	upregulated proteins
**Slc44a1**	-0.6809051	27.6130318	-2.7291553	0.030206215	0.18962708	-3.6417018	downregulated proteins
**Chid1**	0.92563411	22.1292308	2.72902414	0.030211853	0.18962708	-3.6418874	upregulated proteins
**Acot2**	0.82313066	21.8567387	2.72866101	0.03022746	0.18962708	-3.6424011	upregulated proteins
**Blmh**	0.70346955	25.1899807	2.72672107	0.030310986	0.18962708	-3.6451454	upregulated proteins
**Gprc5b**	0.70281783	23.6379111	2.72127565	0.03054674	0.18962708	-3.6528496	upregulated proteins
**Plppr4**	-0.6452166	25.6475495	-2.7133468	0.030893462	0.19112199	-3.6640701	downregulated proteins
**Arpc5l**	-0.6261542	23.0863684	-2.7099714	0.031042316	0.19152523	-3.6688478	downregulated proteins
**Cnnm1**	0.78847614	20.8800024	2.69439147	0.031739205	0.19364722	-3.6909071	upregulated proteins
**Hspa8**	0.59842246	25.2489646	2.69369774	0.031770615	0.19364722	-3.6918896	upregulated proteins
**F3**	-0.7117845	29.9368144	-2.6827091	0.032272508	0.19489255	-3.7074552	downregulated proteins
**Glod4**	0.50944474	25.4203672	2.67757604	0.032509797	0.19504426	-3.7147282	upregulated proteins
**Dld**	0.71000353	23.2188931	2.66407499	0.033142679	0.19623884	-3.7338628	upregulated proteins
**Rps6**	0.83669647	24.2679858	2.66163458	0.033258447	0.19623884	-3.7373223	upregulated proteins
**Atp5f1c**	1.00806003	20.0695853	2.63706535	0.034447803	0.2021821	-3.7721638	upregulated proteins
**Ndrg1**	0.51263235	23.9558526	2.62897646	0.034849034	0.20298338	-3.7836393	upregulated proteins
**Myl12b**	1.28526217	23.275955	2.62894994	0.034850357	0.20298338	-3.7836769	upregulated proteins
**Got2**	0.81622217	24.1760475	2.620747	0.035262249	0.20382648	-3.7953163	upregulated proteins
**Lmnb1**	0.56246561	25.9946873	2.60807569	0.035908526	0.20651913	-3.8133	upregulated proteins

**Ndufb3**	0.61628748	26.5857354	2.60207438	0.036218912	0.20706455	-3.821819	upregulated proteins
**Plpbp**	0.66922495	25.7634331	2.60100062	0.036274741	0.20706455	-3.8233433	upregulated proteins
**Slc25a22**	0.64928692	23.2603492	2.5825512	0.037248141	0.2108132	-3.8495387	upregulated proteins
**Cplx1**	0.55990797	23.9439139	2.57888859	0.037444598	0.2108132	-3.85474	upregulated proteins
**Rab3c**	-0.5171515	27.8910175	-2.5709311	0.037875155	0.2112128	-3.8660415	downregulated proteins
**Atp5f1b**	0.73451503	21.6842667	2.56897182	0.037981952	0.2112128	-3.8688243	upregulated proteins
**Fkbp1a**	1.08347151	22.9234956	2.56710997	0.038083731	0.2112128	-3.8714688	upregulated proteins
**Col7a1**	0.58554618	22.7707183	2.56657126	0.038113233	0.2112128	-3.8722339	upregulated proteins
**Mapk3**	0.51712622	24.0307712	2.55635117	0.038677447	0.21182959	-3.8867514	upregulated proteins
**Bcat1**	0.58707692	21.9062371	2.55436463	0.038788122	0.21189979	-3.8895734	upregulated proteins
**Gap43**	-0.6431048	28.2996077	-2.5490999	0.039083028	0.2124313	-3.8970528	downregulated proteins
**Hsd17b11**	-0.7361154	23.0796289	-2.5414198	0.03951741	0.21262684	-3.907964	downregulated proteins
**Ndufv1**	-0.5638663	25.7723351	-2.5373065	0.039752121	0.21262684	-3.9138083	downregulated proteins
**Dnajc3**	0.53283185	23.3387235	2.53471473	0.039900752	0.21262684	-3.9174908	upregulated proteins
**Ndufb5**	0.59925782	23.9148325	2.53205973	0.040053607	0.21262684	-3.9212633	upregulated proteins
**Edil3**	-1.0355072	22.500499	-2.5300768	0.040168168	0.21262684	-3.9240808	downregulated proteins
**Asrgl1**	-0.5613587	26.0653894	-2.5223523	0.040617683	0.21373305	-3.9350569	downregulated proteins
**Grm1**	1.69859489	20.3198975	2.51072232	0.041304344	0.21408511	-3.9515834	upregulated proteins
**Cnp**	0.94456488	21.9666849	2.50821545	0.041453925	0.21408511	-3.9551458	upregulated proteins
**Gcsh**	-0.6052461	26.6434681	-2.5058757	0.041594042	0.21408511	-3.9584708	downregulated proteins
**Sorl1**	-1.9111632	24.8287551	-2.5054627	0.041618826	0.21408511	-3.9590577	downregulated proteins
**Ndufa3**	0.53454154	25.3157738	2.50450528	0.041676337	0.21408511	-3.9604182	upregulated proteins
**Pcmt1**	0.53410462	19.995122	2.50283713	0.041776739	0.21408511	-3.9627889	upregulated proteins
**Tomm22**	-0.7765633	24.7483491	-2.5023365	0.041806922	0.21408511	-3.9635003	downregulated proteins
**Alg2**	-0.7697522	21.9878793	-2.4934944	0.042343709	0.21634989	-3.9760659	downregulated proteins
**Hnrnpm**	14.2504641	17.6634911	2.48776256	0.042695486	0.21653082	-3.9842115	upregulated proteins
**Rps14**	-1.4573526	21.9830646	-2.4674762	0.043964992	0.22119273	-4.0130403	downregulated proteins
**Aldh18a1**	-0.611222	23.5984593	-2.4640936	0.044180438	0.22177417	-4.0178471	downregulated proteins
**Txndc5**	0.70655331	22.678672	2.45572806	0.04471796	0.22344028	-4.0297346	upregulated proteins
**Slc25a5**	0.52802604	23.7499019	2.45381662	0.044841724	0.22344028	-4.0324507	upregulated proteins
**Atp1a3**	0.60921456	26.5622563	2.44604385	0.045348656	0.2241967	-4.0434952	upregulated proteins
**Uqcr10**	0.75066558	23.3251669	2.41799336	0.047227768	0.22758813	-4.0833462	upregulated proteins
**Nfu1**	-0.60874	19.5880912	-2.4049098	0.048131494	0.23028741	-4.1019293	downregulated proteins
**Sparcl1**	-0.6461925	24.302886	-2.3996171	0.048502122	0.23081363	-4.1094457	downregulated proteins
**Folh1**	2.18272715	24.2569966	2.39313472	0.048960051	0.23233046	-4.1186506	upregulated proteins
**Pfkm**	0.62786828	26.6219769	2.39223644	0.049023858	0.23233046	-4.1199261	upregulated proteins
**Rpl27**	-0.7549196	23.1490517	-2.3868273	0.049409888	0.2336761	-4.1276061	downregulated proteins

Discovery Analysis (10M protein list)

Proteins	logFC	AveExpr	t	P.Value	adj.P.Val	B	Category
**Hprt1**	-1.3591324	24.1722662	-8.9238509	5.33737222260029E-05	0.12313318	-0.8561095	downregulated proteins
**Glo1**	0.91313895	21.8790261	6.09250387	0.000551956	0.43340542	-1.47807	upregulated proteins
**Atp6v1g1**	1.60959423	22.3616801	5.7949931	0.000738898	0.43340542	-1.5791092	upregulated proteins
**S100a1**	-0.5542213	23.4467489	-5.7781205	0.000751461	0.43340542	-1.585123	downregulated proteins
**Kcnj10**	-1.1193064	21.5332552	-5.258723	0.001284964	0.5928822	-1.7865892	downregulated proteins
**Ndufaf2**	0.53613059	25.7252552	4.73051395	0.002300385	0.79570878	-2.0278838	upregulated proteins
**Rida**	0.5633735	22.3010775	4.68818246	0.002414374	0.79570878	-2.0489936	upregulated proteins
**Ppp1ca**	0.78565381	23.9099429	4.53657282	0.00287709	0.82968093	-2.126905	upregulated proteins
**Fabp3**	0.88162628	21.5240438	4.37895571	0.003464728	0.84328435	-2.2118457	upregulated proteins
**Tusc3**	0.81559011	25.4148102	4.12456277	0.004713361	0.84328435	-2.3578067	upregulated proteins
**Gsta4**	0.51273865	23.4088259	4.11585821	0.004764078	0.84328435	-2.3630008	upregulated proteins
**Psap**	0.58561107	26.7122038	4.06469839	0.00507456	0.84328435	-2.3938011	upregulated proteins
**Arf6**	0.61333384	23.4197484	3.82702019	0.006839718	0.84328435	-2.5431096	upregulated proteins
**Sacm1l**	-0.6786373	27.3460579	-3.7656408	0.007398092	0.84328435	-2.5833593	downregulated proteins
**App**	2.0042263	22.0021863	3.5859904	0.009338667	0.84328435	-2.7052276	upregulated proteins
**Dclk1**	0.72723989	18.7310656	3.57724352	0.009446346	0.84328435	-2.7113168	upregulated proteins
**Samm50**	0.6878859	24.4888419	3.5244965	0.010124956	0.84328435	-2.748344	upregulated proteins
**Got1**	1.23645398	19.4420564	3.45636374	0.01108087	0.84328435	-2.7969529	upregulated proteins
**Cds1**	-0.9986552	26.0466543	-3.4469656	0.011220224	0.84328435	-2.8037271	downregulated proteins
**Ppp2r5c**	0.90904093	21.6671231	3.43272106	0.011435066	0.84328435	-2.8140264	upregulated proteins
**Tpt1**	0.80749832	19.0451233	3.32889843	0.013141945	0.84328435	-2.8902551	upregulated proteins
**Ist1**	-16.97526	13.990565	-3.2837328	0.013968584	0.84328435	-2.9240517	downregulated proteins
**Uqcrh**	0.52354015	21.8145164	3.26249844	0.01437642	0.84328435	-2.9400734	upregulated proteins
**Sncb**	0.55302926	21.1520685	3.22433412	0.015141968	0.84328435	-2.9690812	upregulated proteins
**Txnrd1**	-1.4130187	21.7315102	-3.1462263	0.01684868	0.84328435	-3.0292932	downregulated proteins
**Calb2**	0.61475863	23.4622562	3.13922606	0.017011412	0.84328435	-3.0347446	upregulated proteins
**Vamp2**	-0.8009784	29.6407305	-3.1157294	0.017570048	0.84328435	-3.053108	downregulated proteins
**Uqcrq**	-0.6282777	25.636655	-3.0833216	0.018372967	0.84328435	-3.0786011	downregulated proteins
**Pdcd6**	15.1191802	12.7887747	3.07661944	0.01854386	0.84328435	-3.083897	upregulated proteins
**Lgi3**	0.86566723	24.9640087	3.02756051	0.019847632	0.84328435	-3.122909	upregulated proteins
**Pvalb**	-0.5485745	21.8407185	-3.0164737	0.020155604	0.84328435	-3.131785	downregulated proteins
**Kcna1**	1.18960055	22.5914118	2.95001137	0.022111748	0.84328435	-3.1854494	upregulated proteins
**Hcn2**	-1.0825403	24.3701039	-2.93652	0.022532931	0.84328435	-3.1964372	downregulated proteins
**Pdp1**	0.76053644	23.4722155	2.92369716	0.022941144	0.84328435	-3.2069096	upregulated proteins
**Atp5if1**	-1.2110442	20.6425329	-2.9021729	0.023644104	0.84328435	-3.224552	downregulated proteins
**Arpc4**	-0.6046128	23.9102639	-2.8901904	0.024045298	0.84328435	-3.2344076	downregulated proteins
**Ube2l3**	0.89605884	24.7569467	2.79416116	0.027532805	0.84328435	-3.3142587	upregulated proteins
**Cndp2**	0.52157892	24.2390238	2.77210898	0.02840669	0.84328435	-3.3328084	upregulated proteins

**Rab2a**	0.58777799	25.6048852	2.75400788	0.029145819	0.84328435	-3.3480923	upregulated proteins
**Krt73**	1.07196738	22.4416605	2.74216732	0.029640235	0.84328435	-3.3581179	upregulated proteins
**Acyp2**	0.7931481	21.8162555	2.72515736	0.03036599	0.84328435	-3.3725587	upregulated proteins
**Vamp3**	1.04556214	24.5937094	2.7088304	0.031080174	0.84328435	-3.3864616	upregulated proteins
**Rpl28**	0.90315087	22.0418269	2.70792166	0.031120439	0.84328435	-3.3872367	upregulated proteins
**Vat1**	0.61808603	20.2796694	2.69887867	0.031524117	0.84328435	-3.3949558	upregulated proteins
**Ldhb**	0.64804367	23.1090864	2.69229948	0.03182126	0.84328435	-3.4005796	upregulated proteins
**Pde1b**	1.32390082	21.3739929	2.68885498	0.031977997	0.84328435	-3.4035265	upregulated proteins
**Nrxn2**	0.69220047	22.9354773	2.68278262	0.032256285	0.84328435	-3.4087259	upregulated proteins
**Slc38a3**	0.67236117	20.7282983	2.64931163	0.03383641	0.84328435	-3.4374834	upregulated proteins
**Kcnab2**	0.63982274	22.1007952	2.64205921	0.034189341	0.84328435	-3.4437361	upregulated proteins
**Marcksl1**	0.87866852	24.8236682	2.6391932	0.034329871	0.84328435	-3.4462091	upregulated proteins
**Atp6v1e1**	1.25709401	25.4407655	2.63403842	0.034584148	0.84328435	-3.4506601	upregulated proteins
**Pex5l**	0.68569177	19.7381547	2.60789377	0.035904453	0.84328435	-3.4732931	upregulated proteins
**Hspa4l**	0.52718493	24.0352174	2.5932763	0.036665455	0.84328435	-3.4859889	upregulated proteins
**Anxa6**	13.0834674	15.8568168	2.58967951	0.036855273	0.84328435	-3.4891174	upregulated proteins
**Pfn2**	1.38395926	22.211542	2.56762367	0.038041856	0.84328435	-3.5083401	upregulated proteins
**Acyp1**	-1.7228525	23.2514449	-2.5158533	0.040985745	0.84328435	-3.5537133	downregulated proteins
**Praf2**	0.64988461	26.3906922	2.51278405	0.041167538	0.84328435	-3.5564142	upregulated proteins
**Nme1**	0.87877659	24.0273759	2.50343339	0.041726543	0.84328435	-3.5646497	upregulated proteins
**Napa**	0.56675199	20.6400703	2.49167497	0.042440671	0.84328435	-3.5750211	upregulated proteins
**Cyc1**	-0.9108774	21.2011109	-2.4824622	0.043009029	0.84328435	-3.583159	downregulated proteins
**Zfyve28**	0.67724851	22.9971235	2.47990186	0.043168375	0.84328435	-3.5854224	upregulated proteins
**Nbea**	-1.545281	23.1938634	-2.4732031	0.043588181	0.84328435	-3.5913481	downregulated proteins
**Ndufab1**	0.50558271	22.26684	2.45546667	0.044720203	0.84328435	-3.6070633	upregulated proteins
**Calb1**	-1.1225659	21.0398619	-2.4534005	0.044854038	0.84328435	-3.6088964	downregulated proteins
**Aldh6a1**	1.003151	23.3906142	2.4355493	0.04602768	0.84328435	-3.6247543	upregulated proteins
**Uqcrb**	0.68695573	23.0320414	2.42284057	0.046882489	0.84328435	-3.6360659	upregulated proteins
**Enoph1**	-0.9657279	28.8423718	-2.4111622	0.04768241	0.84328435	-3.646476	downregulated proteins
**Cntnap1**	0.93791659	23.2589386	2.40852314	0.047865116	0.84328435	-3.6488305	upregulated proteins
