# Supplementary material for: Proteomic analysis links truncated tau to lysosome motility, autophagy, and endo‐lysosomal dysfunction
Source: Alzheimers Dement. 2025 Dec 15;21(12):e70977. doi: 10.1002/alz.70977 (PMC12706120; doi:10.1002/alz.70977)
Supplement: Supplementary file 7 — Supporting Information [file ALZ-21-e70977-s001.pdf]

Supplemental file 2

A SH-SY5Y tauopathy cell line expression plasmid maps

Tau35-HA

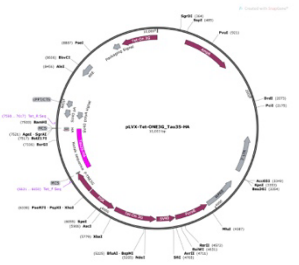

(Tau35 overexpressing)

Avi-FL tau

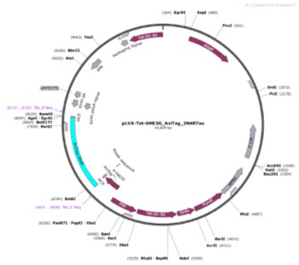

(FL tau overexpressing)

mKO_2-Tau35

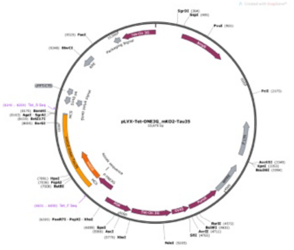

(Tau35 overexpressing)

Tau35-mKO_2

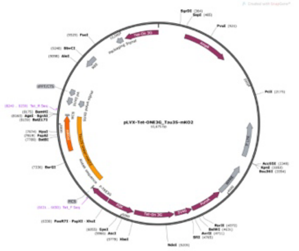

(Tau35 overexpressing)

mEGFP-FL tau

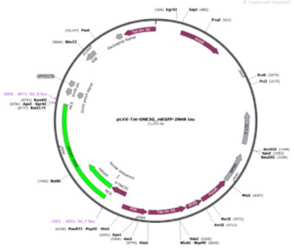

(FL tau overexpressing)

FL tau-mEGFP

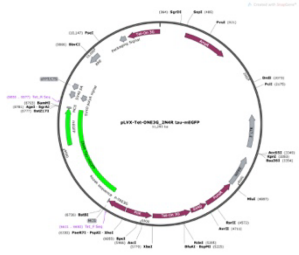

(FL tau overexpressing)

B Protein expression in SH-SY5Y cell lines

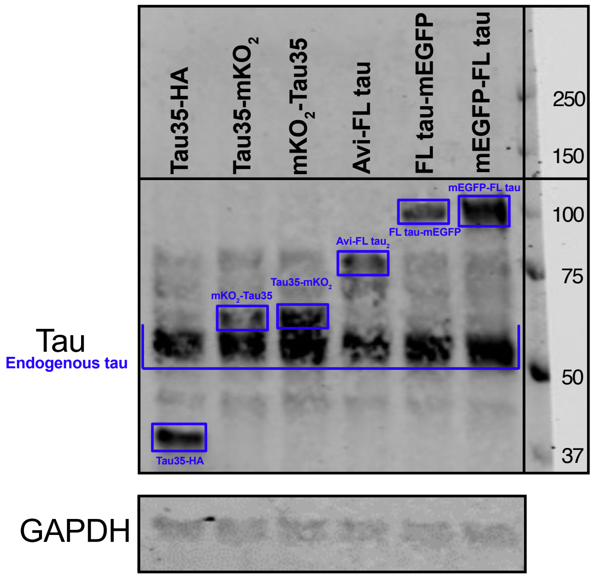

C SH-SY5Y tauopathy and control cell lines

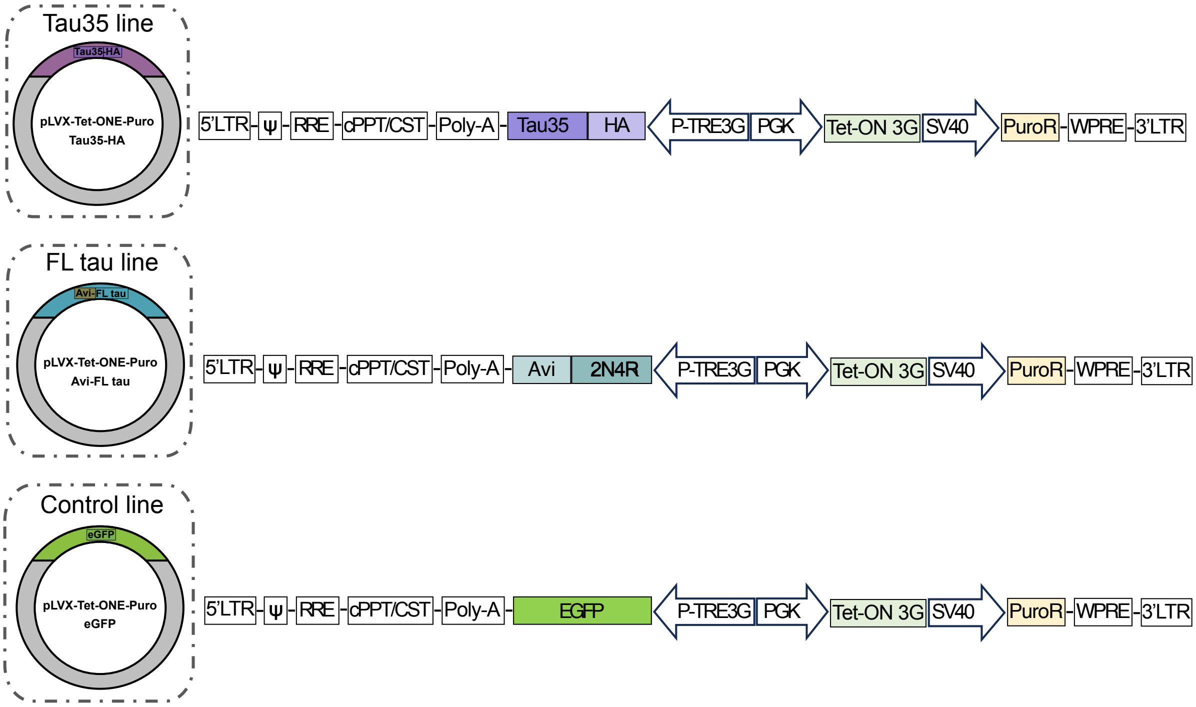

D Characterization of differentiated SH-SY5Y tauopathy and control cell lines

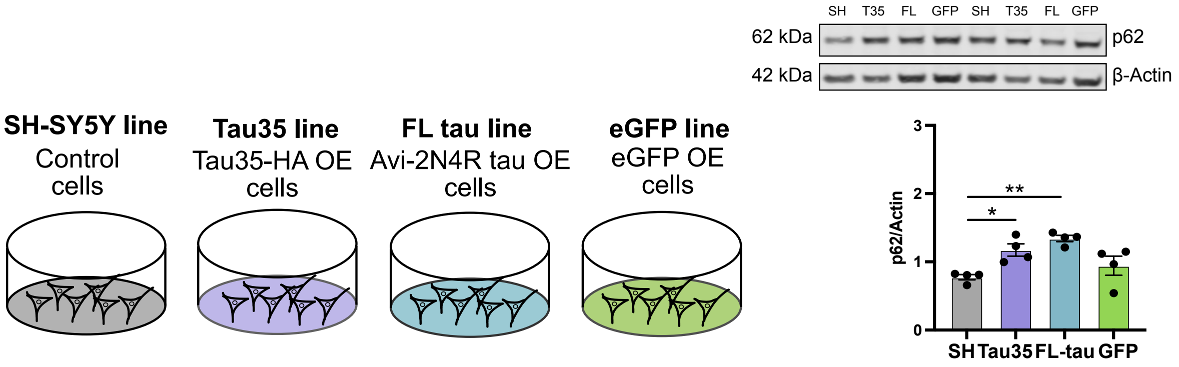

(A) SH-SY5Y tauopathy cell line maps. Expression plasmids for all six novel SH-SY5Y tauopathy cell lines. Generalized maps of plasmids used for the expression of truncated (Tau35) or full length (2N4R) tau. **(B)** Western blot analysis of cell lysates from all stable SH-SY5Y cell lines overexpressing tau was conducted using antibodies specific to tau and GAPDH. The blot revealed both endogenous tau and the overexpressed fusion proteins, which are highlighted. **(C)** Generation of stable cell lines harboring an inducible pLVX-Tet-ONE vector expressing Tau35-HA (Tau35 line), Avi-FL tau (FL-tau line) and eGFP (GFP line); eGFP, enhanced green fluorescent protein . **(D)** Schematic illustrating the four differentiated SH-SY5Y cell lines; control SH-SY5Y cells, cells overexpressing (OE) Tau35-HA (referred to as Tau35) and cells overexpressing (OE) Avi-FL tau (referred to as FL-tau) and eGFP (referred to as GFP). Western blot of total cell lysates from control, Tau35, and FL-tau, GFP differentiated cells, collected at 14 days in vitro (DIV), were probed with antibodies to p62 and GAPDH. Quantification of the blots is shown in the graphs as mean ± SEM, $n = 4$ independent experiments. Ordinary one-way ANOVA, $^{*}P < 0.05$, $^{**}P < 0.01$. p62/SQSTM1, Sequestosome-1; GAPDH, glyceraldehyde 3-phosphate dehydrogenase; SEM, standard error of the mean.
