# Supplementary material for: Proteomic analysis links truncated tau to lysosome motility, autophagy, and endo‐lysosomal dysfunction
Source: Alzheimers Dement. 2025 Dec 15;21(12):e70977. doi: 10.1002/alz.70977 (PMC12706120; doi:10.1002/alz.70977)
Supplement: Supplementary file 8 — Supporting Information [file ALZ-21-e70977-s005.pdf]

Supplemental file 3

Supplementalfile3_Lysotracker_SH-SY5Ycontrolline.avi

Live-cell imaging of differentiated SH-SY5Y control cells stained with LysoTracker Deep Red. Imaging was performed at 100 frames per second using a 640nm laser. Each dot represents an individual lysosome; the trailing lines indicate the trajectory and movement of each lysosome throughout the imaging session, used to assess lysosomal motility parameters.
